# Supplementary material for: Synthesis and Characterization of Novel 2-Acyl-3-trifluoromethylquinoxaline 1,4-Dioxides as Potential Antimicrobial Agents
Source: Pharmaceuticals (Basel). 2022 Jan 27;15(2):155. doi: 10.3390/ph15020155 (PMC8877263; doi:10.3390/ph15020155)
Supplement: Supplementary file 1 [file pharmaceuticals-15-00155-s001.zip › pharmaceuticals-1552569-supplementary.pdf]

Supplementary Materials

Synthesis and characterization of novel 2-acyl-3-trifluoromethylquinoxaline 1,4-dioxides as potential antimicrobial agents

Galina I. Buravchenko[1], Dmitry A. Maslov[2], Md Shah Alam[3,4,5,6], Natalia E. Grammatikova[1], Svetlana G. Frolova[2,7], Aleksey A. Vatlin[2,8], Xirong Tian[3,4,5,6], Ivan V. Ivanov[1,9], Olga B. Bekker[2], Maxim A. Kryakvin[10], Olga A. Dontsova[10,11,12], Valery N. Danilenko[2], Tianyu Zhang[3,4,5,6], Andrey E. Shchekotikhin[1,*]

1 Gause Institute of New Antibiotics, 119021 Moscow, Russia; buravchenkogi@gmail.com (G.I.B.); ngrammatikova@yandex.ru (N.E.G.); ivanvi@yandex.ru (I.V.I.); shchekotikhin@mail.ru (AES)

2 Laboratory of Bacterial Genetics, Vavilov Institute of General Genetics, Russian Academy of Sciences, Moscow, 119333, Russia; maslov_da@vigg.ru (D.A.M.); sveta.frolova.1997@bk.ru (S.G.F.); vatlin_alexey123@mail.ru (A.A.V.); obbekker@mail.ru (O.B.B.); valerid@vigg.ru (V.N.D.)

3 State Key Laboratory of Respiratory Disease, Guangzhou Institutes of Biomedicine and Health, Chinese Academy of Sciences, Guangzhou 510530, China; alam@gibh.ac.cn (M.S.A.); tian_xirong@gibh.ac.cn (X.T.); zhang_tianyu@gibh.ac.cn (T.Z.)

4 China-New Zealand Joint Laboratory on Biomedicine and Health, Guangzhou 510530, China; alam@gibh.ac.cn (Md S.A.); tian_xirong@gibh.ac.cn (X.T.); zhang_tianyu@gibh.ac.cn (T.Z.)

5 Guangdong-Hong Kong-Macao Joint Laboratory of Respiratory Infectious Diseases, Guangzhou Institutes of Biomedicine and Health, Chinese Academy of Sciences, Guangzhou 510530, China; alam@gibh.ac.cn (Md S.A.); tian_xirong@gibh.ac.cn (X.T.); zhang_tianyu@gibh.ac.cn (T.Z.)

6 University of Chinese Academy of Sciences, Beijing 100049, China; alam@gibh.ac.cn (M.S.A.); tian_xirong@gibh.ac.cn (X.T.); zhang_tianyu@gibh.ac.cn (T.Z.)

7 Moscow Institute of Physics and Technology (State University), Dolgoprudny, 141701, Russia; sveta.frolova.1997@bk.ru (S.G.F.)

8 Peoples' Friendship University of Russia (RUDN University), Moscow, 117198, Russia; vatlin_alexey123@mail.ru (A.A.V.)

9 Mendeleyev University of Chemical Technology, 9 Miusskaya Square, Moscow 125190, Russia; ivanvi@yandex.ru (I.V.I.)

10 Chemistry Department, Faculty of Bioengineering and Bioinformatics, Lomonosov Moscow State University, Moscow, Russia; maxim.kryakvin@gmail.com (M.A.K.); olga.a.dontsova@gmail.com (O.A.D.)

11 Center of Life Sciences, Skolkovo Institute of Science and Technology, Skolkovo, 143028, Russia; olga.a.dontsova@gmail.com (O.A.D.)

12 Shemyakin-Ovchinnikov Institute of Bioorganic Chemistry, Moscow, Russia; olga.a.dontsova@gmail.com (O.A.D.)

* Correspondence: shchekotikhin@gause-inst.ru (A.E.S.)

* Corresponding authors.

Andrey E. Shchekotikhin: shchekotikhin@mail.ru

Legends to Figures and Tables

Figure S1-S56, **Table S1**. ^{1}H and ^{13}C NMR spectra of the compounds **6-7a, 7d**, **12-18**. **S3**

Figure S57-S60. Copies of 2D NMR Spectra of compounds **13b** and **13c**. **S32**

Figure S61-S87. Copies of HRMS ESI spectra. **S36**

Figure S88-112. Copies of HPLC spectra. **S63**

Figure S113. Nucleotide sequence of *MSMEG_4883*. **S88**

Table S2. Unique SNPs in quinoxaline-resistant *M. smegmatis* mutants **S89**

Copies of NMR Spectra

Figure S1. Copy of ^1H NMR spectrum of the derivative **6a**.

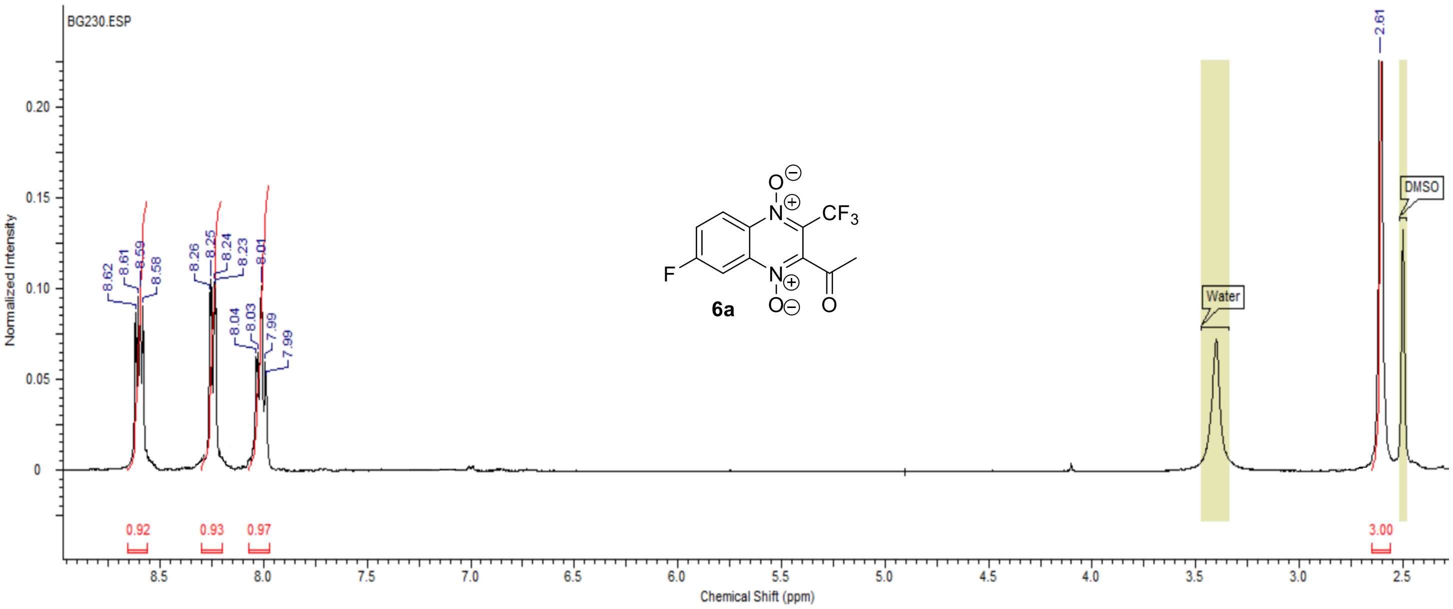

Figure S2. Copy of ^{13}C NMR spectrum of the derivative **6a**.

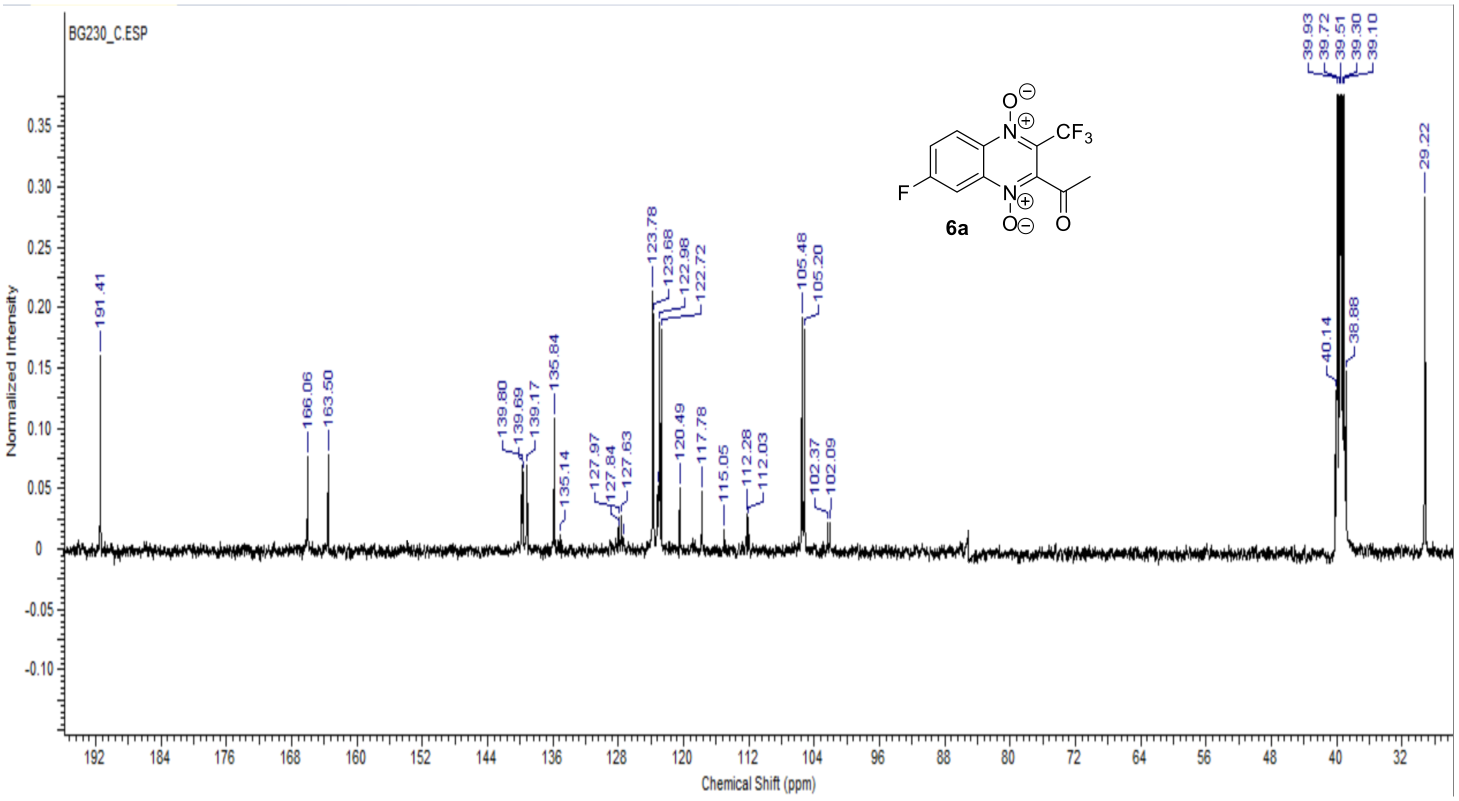

Figure S3. Copy of ^{1}H NMR spectrum of the derivative **7a**.

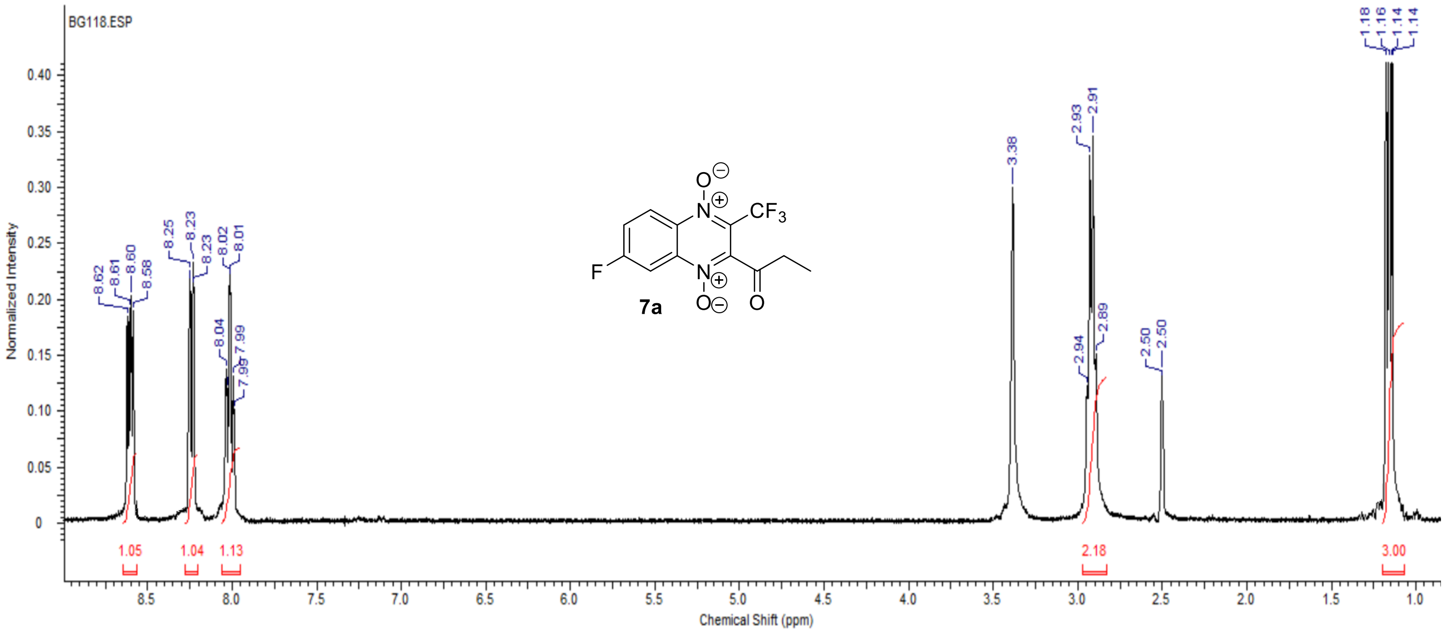

Figure S4. Copy of ^{13}C NMR spectrum of the derivative **7a**.

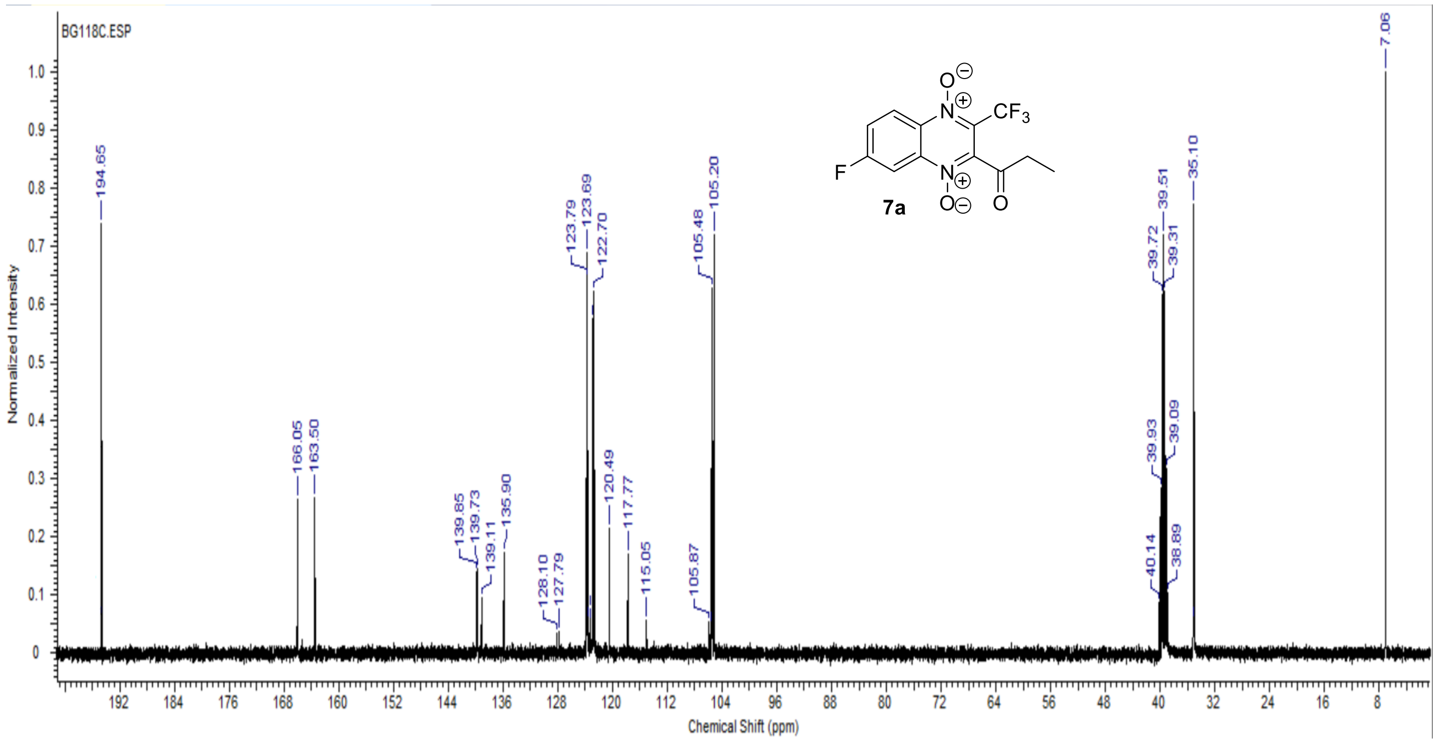

Figure S5. Copy of ^{1}H NMR spectrum of the derivative **12a**.

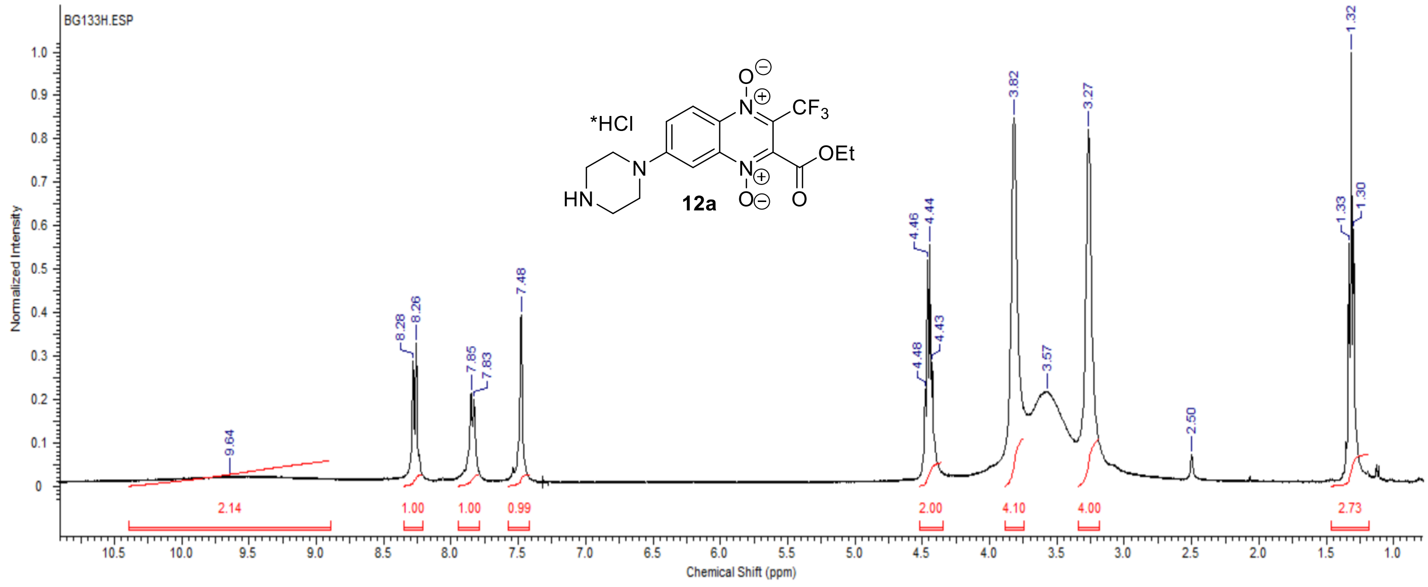

Figure S6. Copy of ^{13}C NMR spectrum of the derivative **12a**.

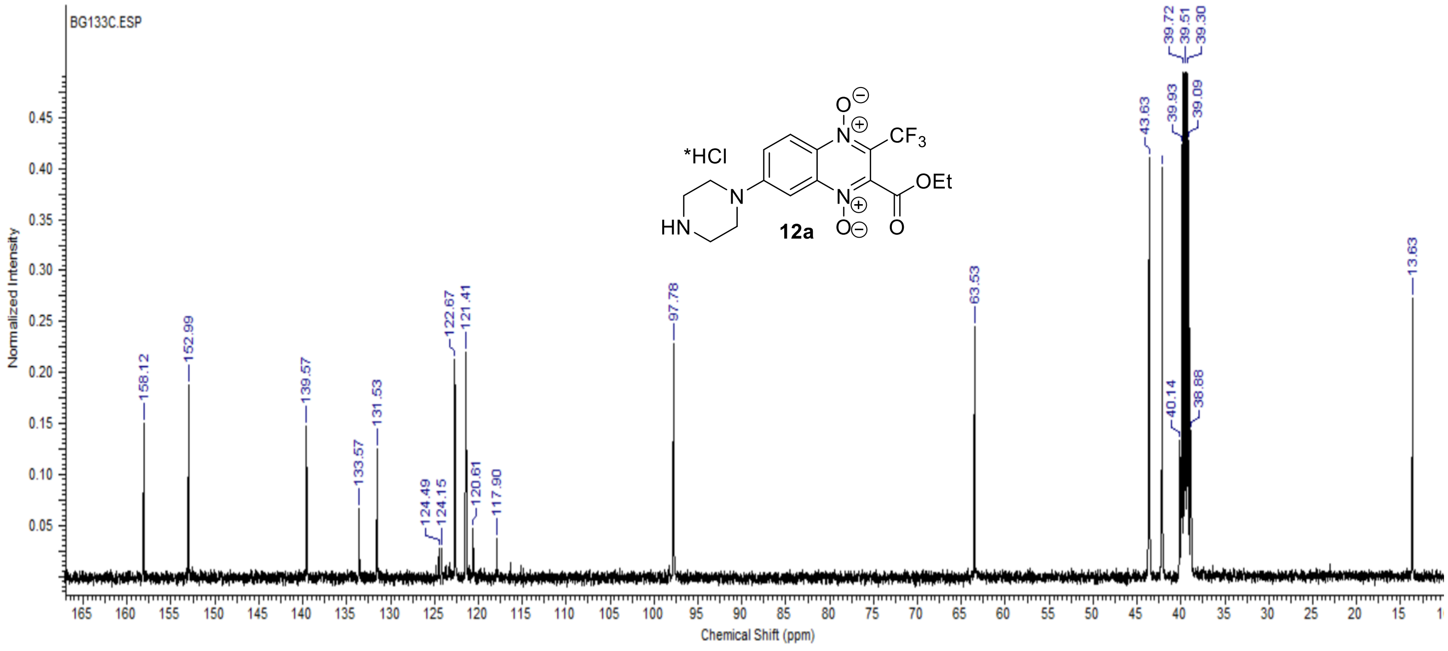

Figure S7. Copy of ^{1}H NMR spectrum of the derivative **12b**.

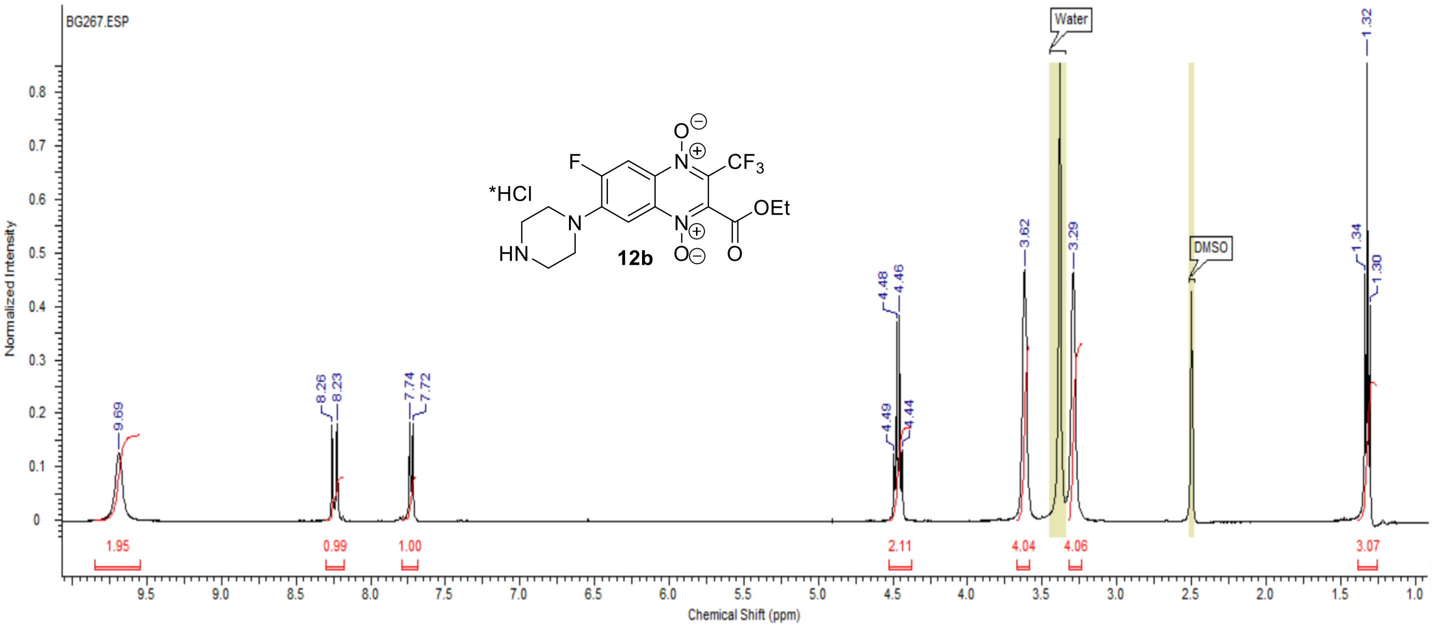

Figure S8. Copy of ^{13}C NMR spectrum of the derivative **12b**.

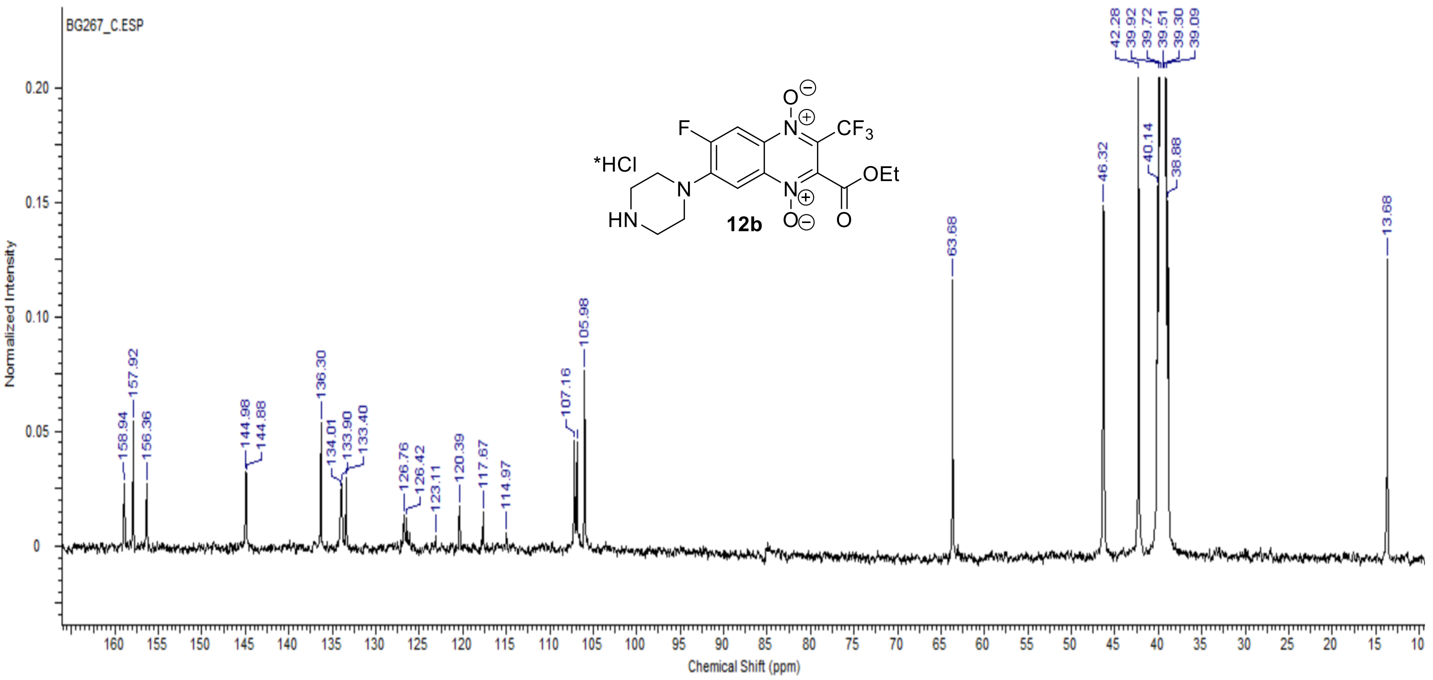

Figure S9. Copy of ^{1}H NMR spectrum of the derivative **12c**.

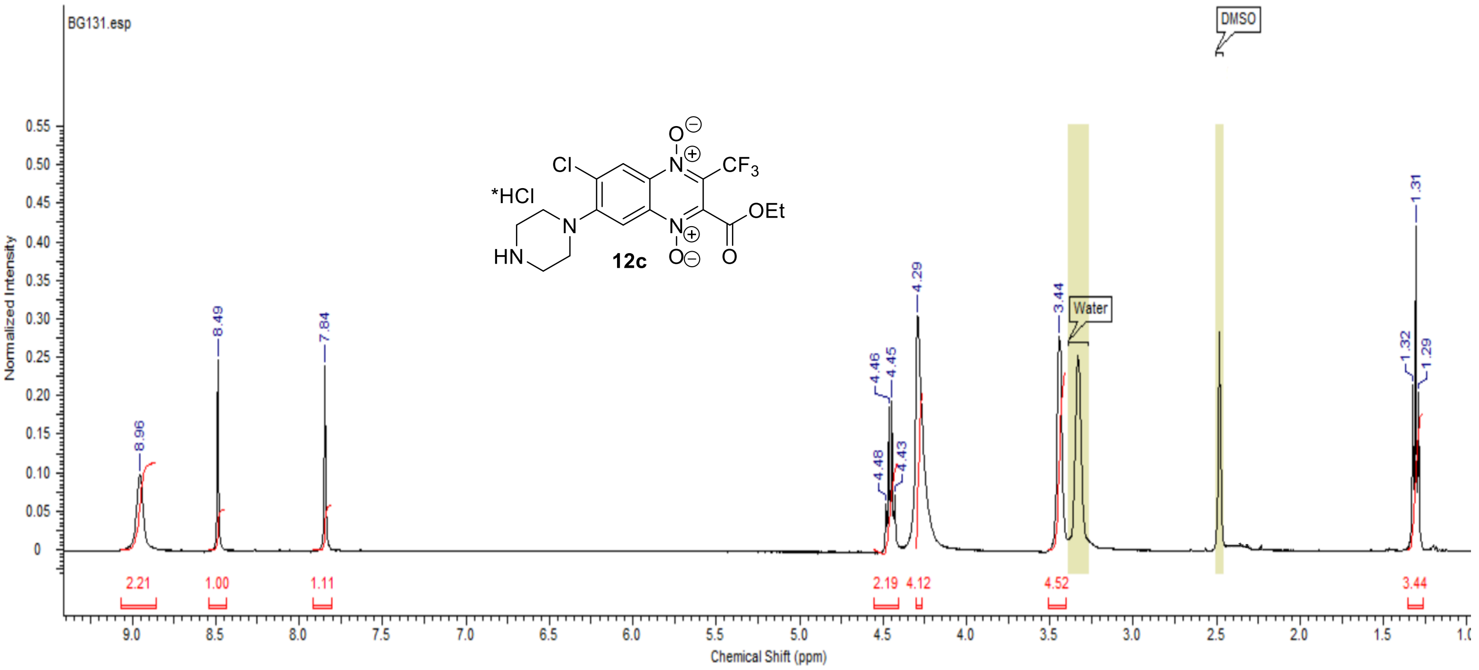

Figure S10. Copy of ^{13}C NMR spectrum of the derivative **12c**.

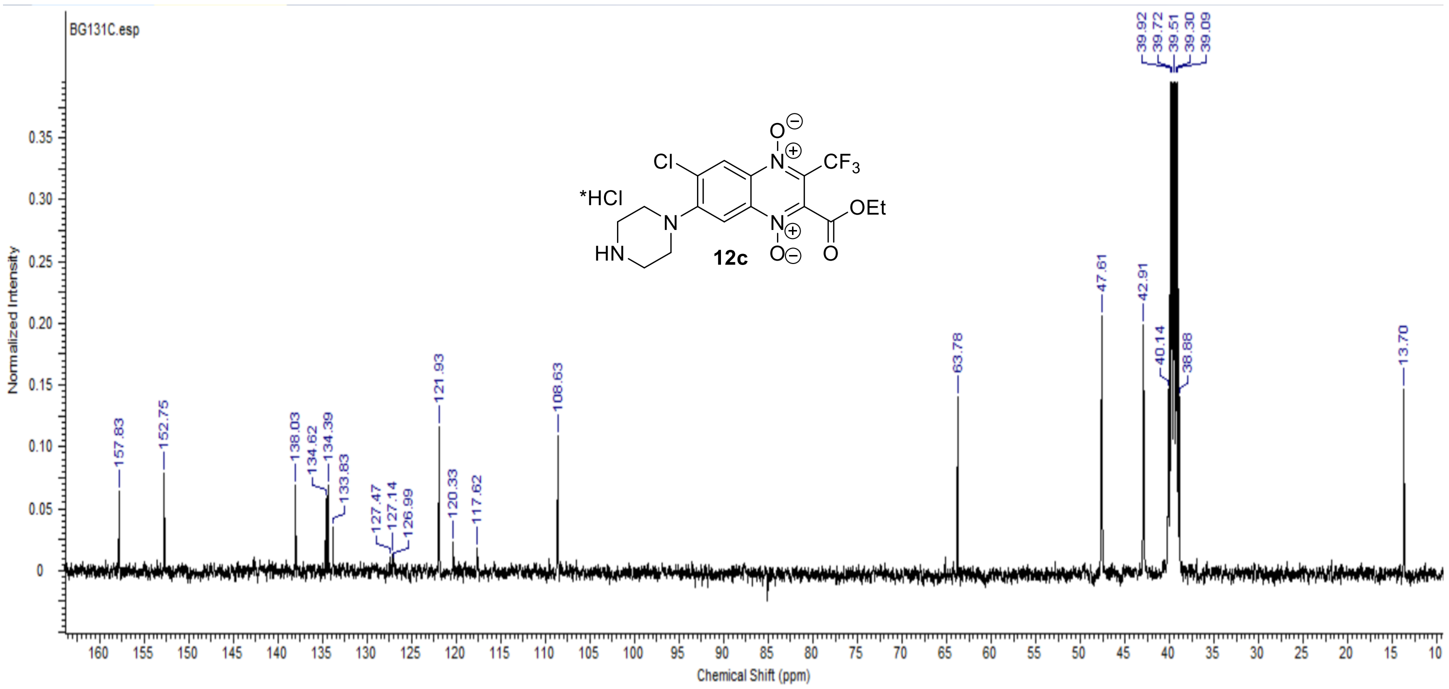

Figure S11. Copy of ^{1}H NMR spectrum of the derivative **13a**.

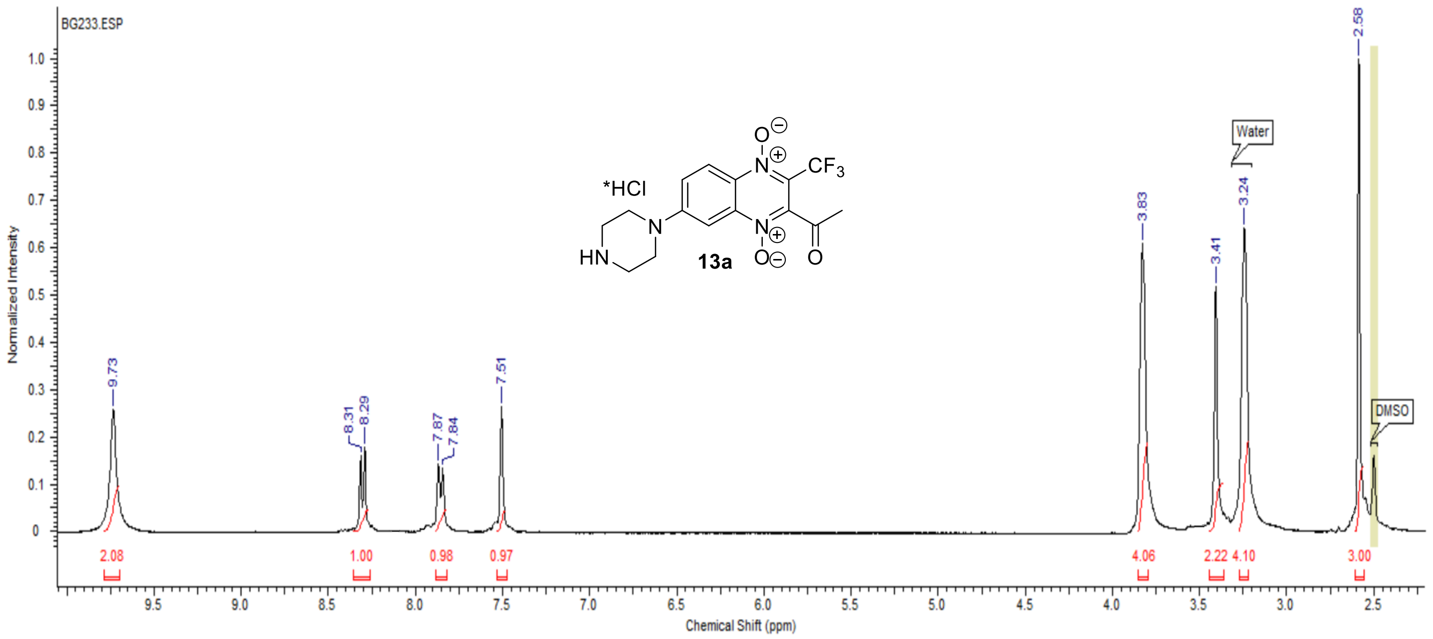

Figure S12. Copy of ^{13}C NMR spectrum of the derivative **13a**.

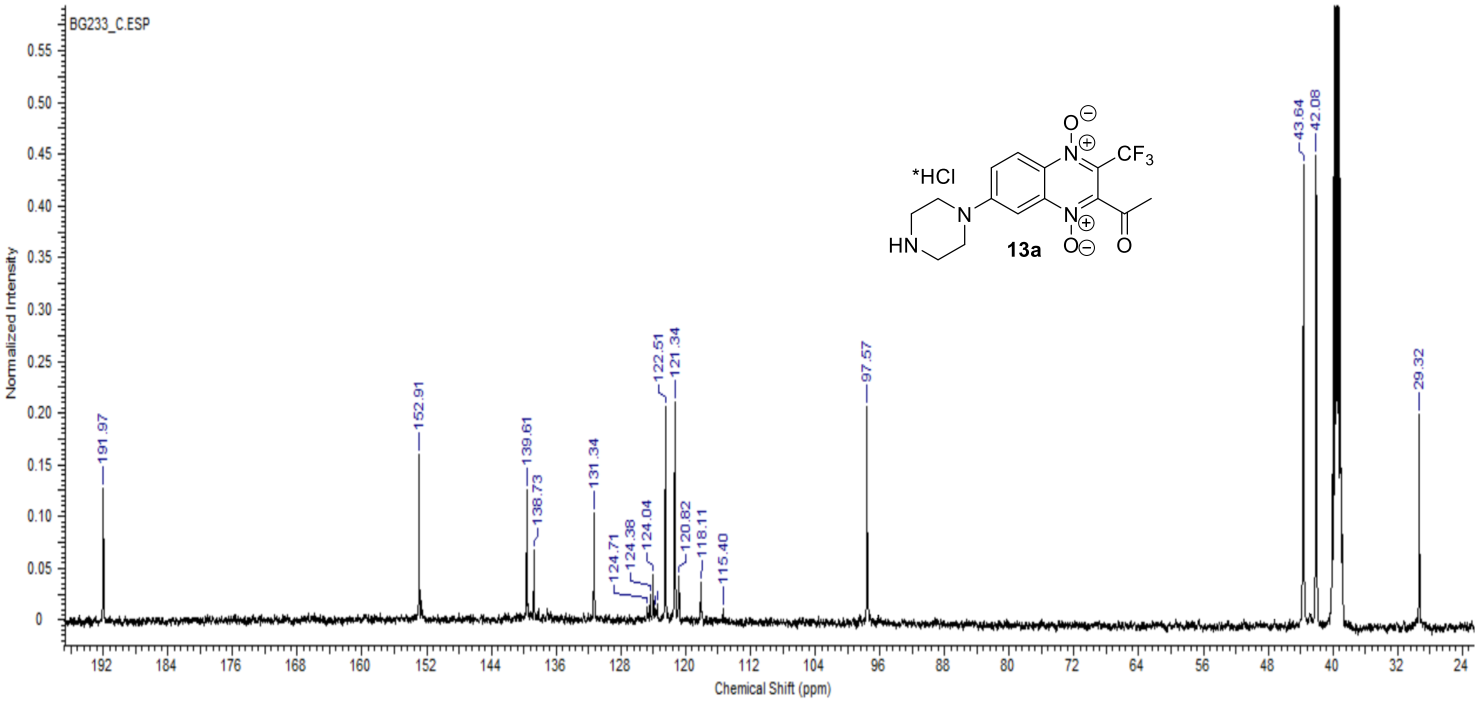

Figure S13. Copy of ^{1}H NMR spectrum of the derivative **13b**.

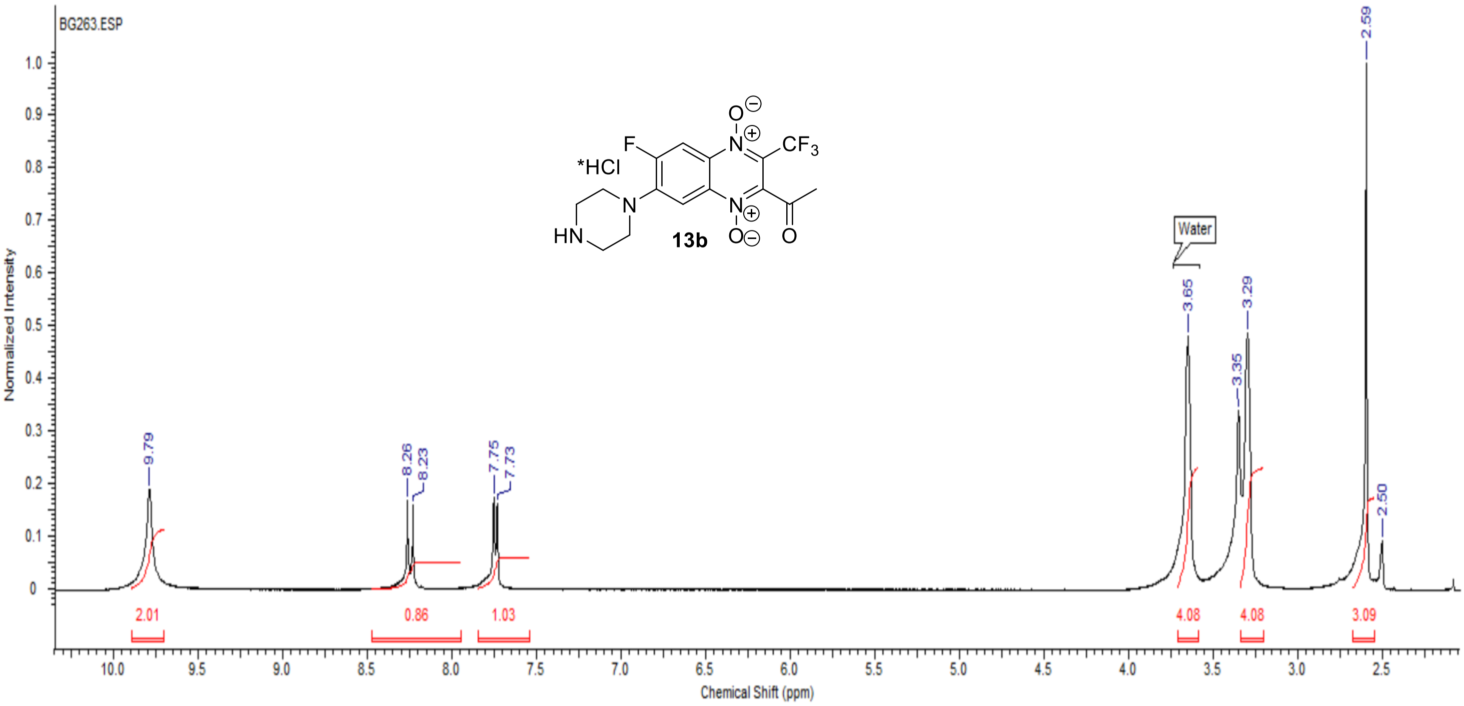

Figure S14. Copy of ^{13}C NMR spectrum of the derivative **13b**.

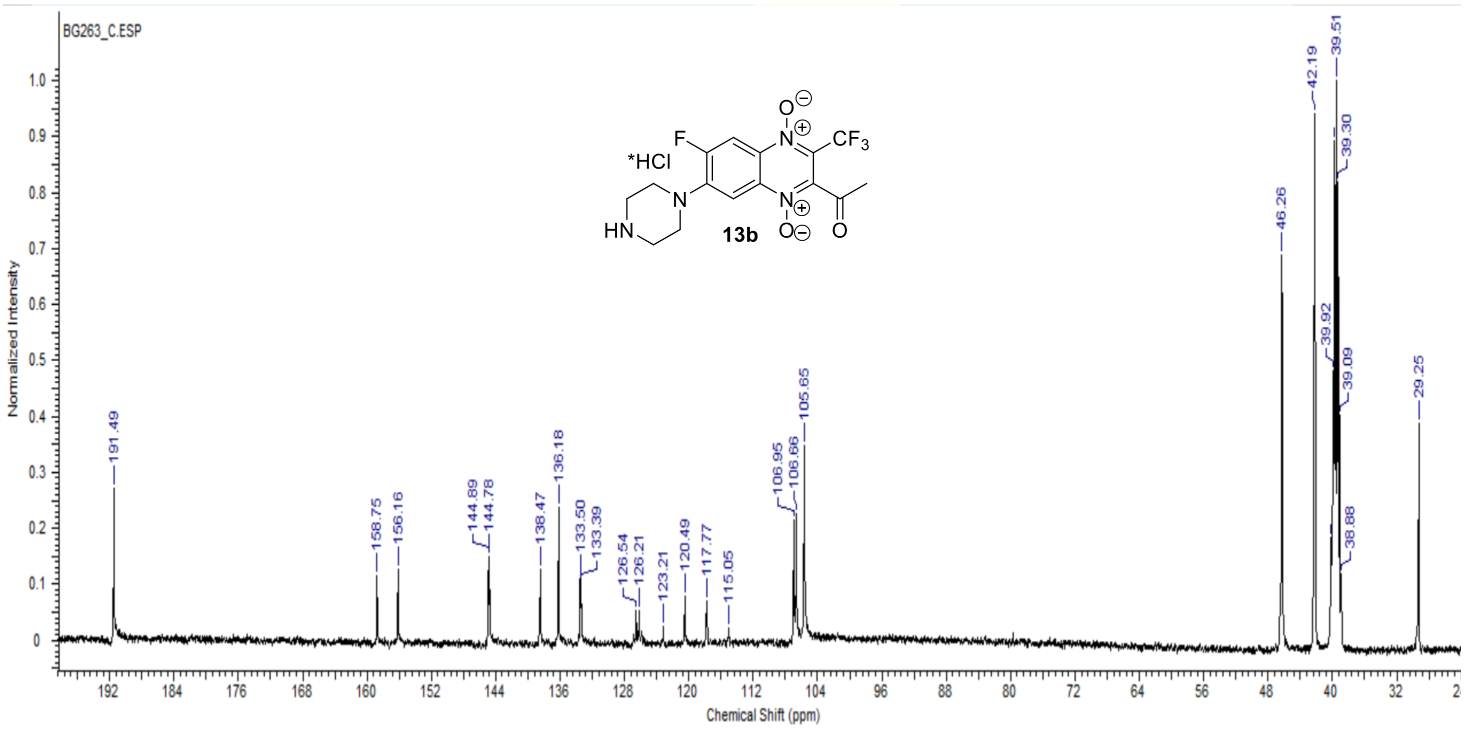

Figure S15. Copy of ^{1}H NMR spectrum of the derivative **13c**.

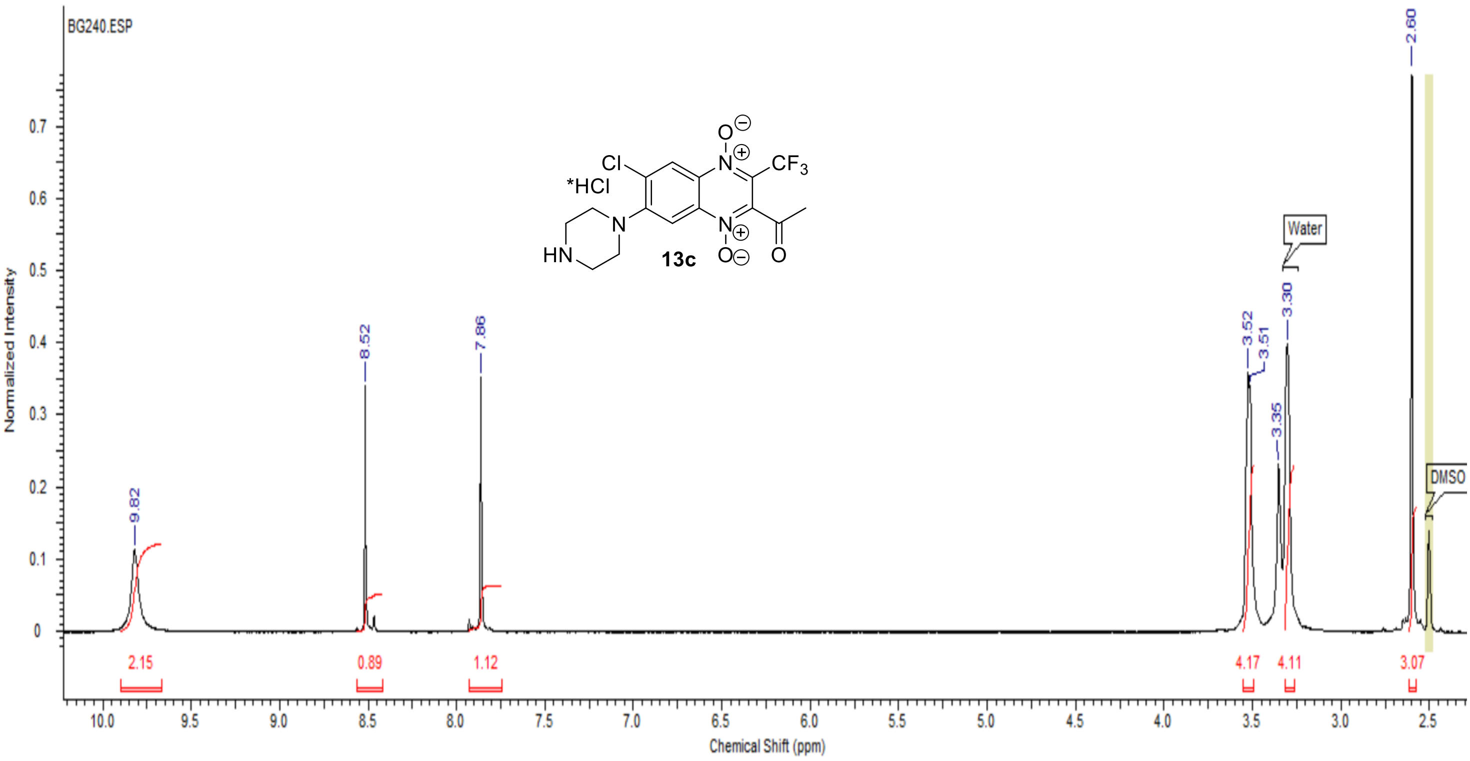

Figure S16. Copy of ^{13}C NMR spectrum of the derivative **13c**.

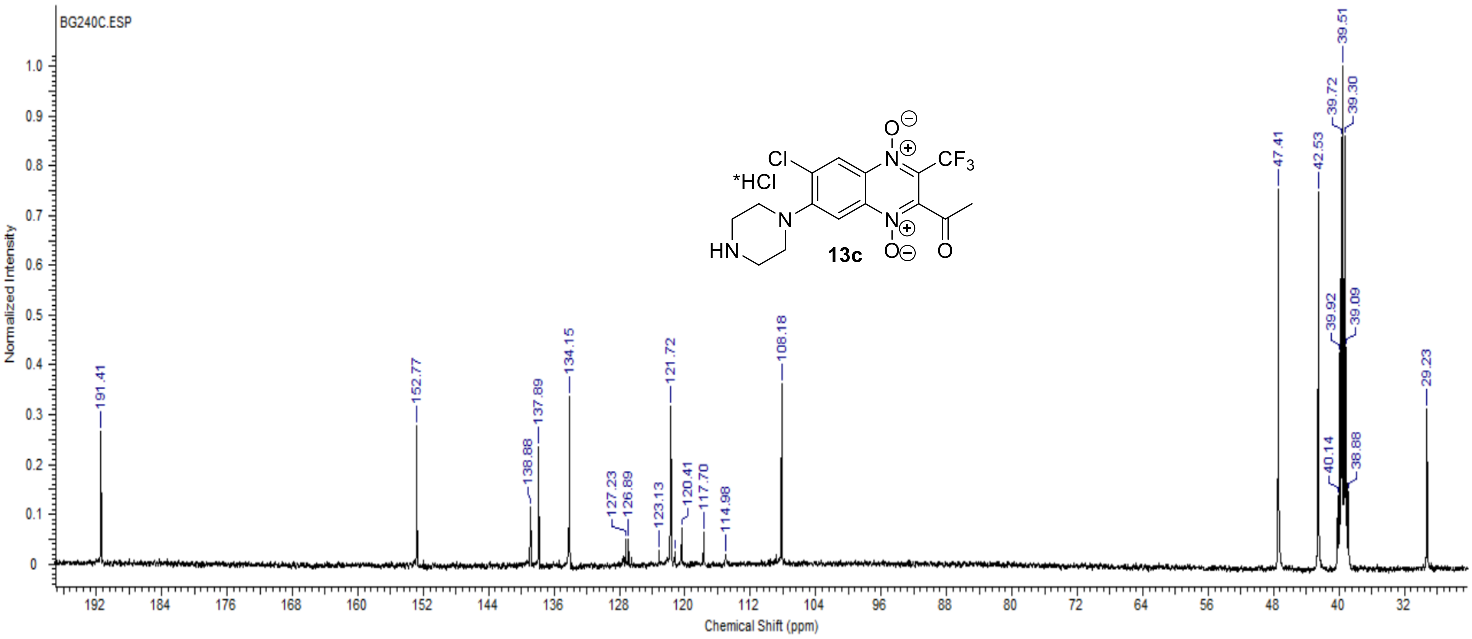

Figure S17. Copy of ^{1}H NMR spectrum of the derivative **14a**.

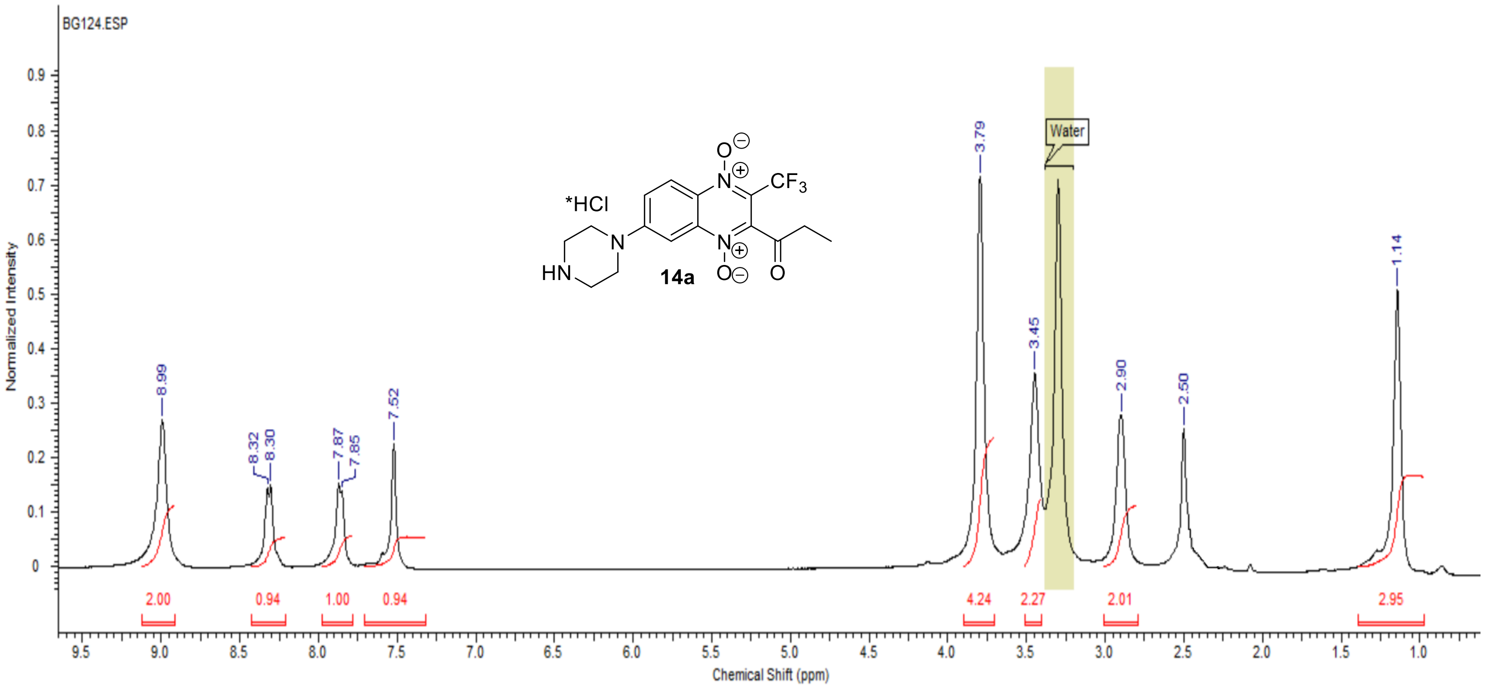

Figure S18. Copy of ^{13}C NMR spectrum of the derivative **14a**.

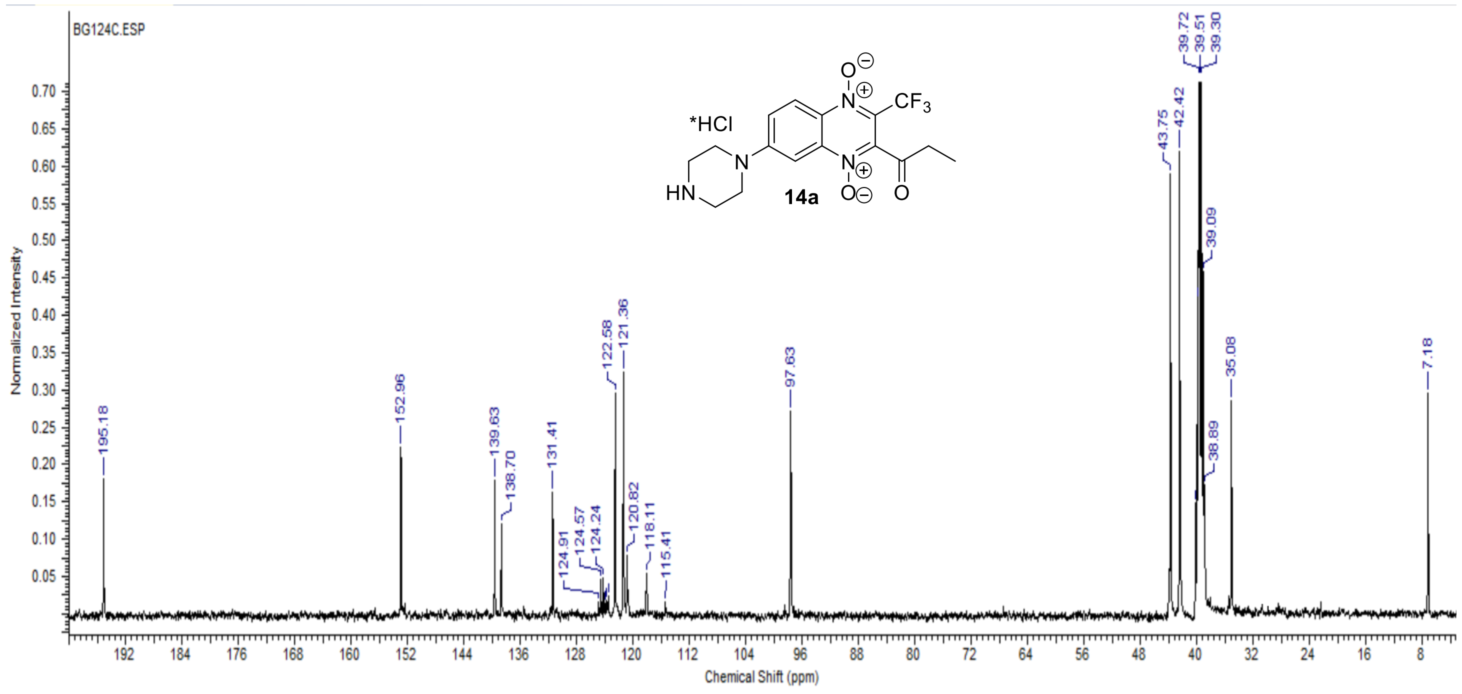

Figure S19. Copy of ^{1}H NMR spectrum of the derivative **14b**.

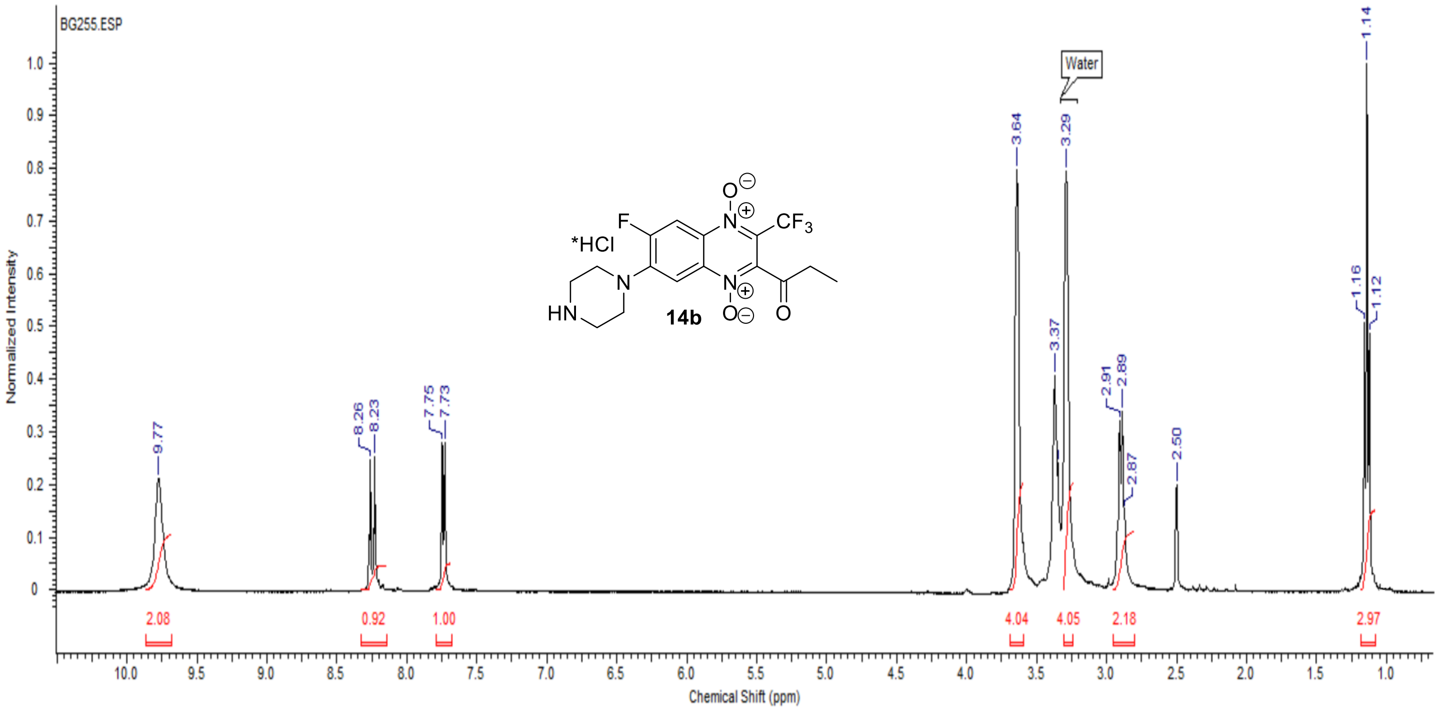

Figure S20. Copy of ^{13}C NMR spectrum of the derivative **14b**.

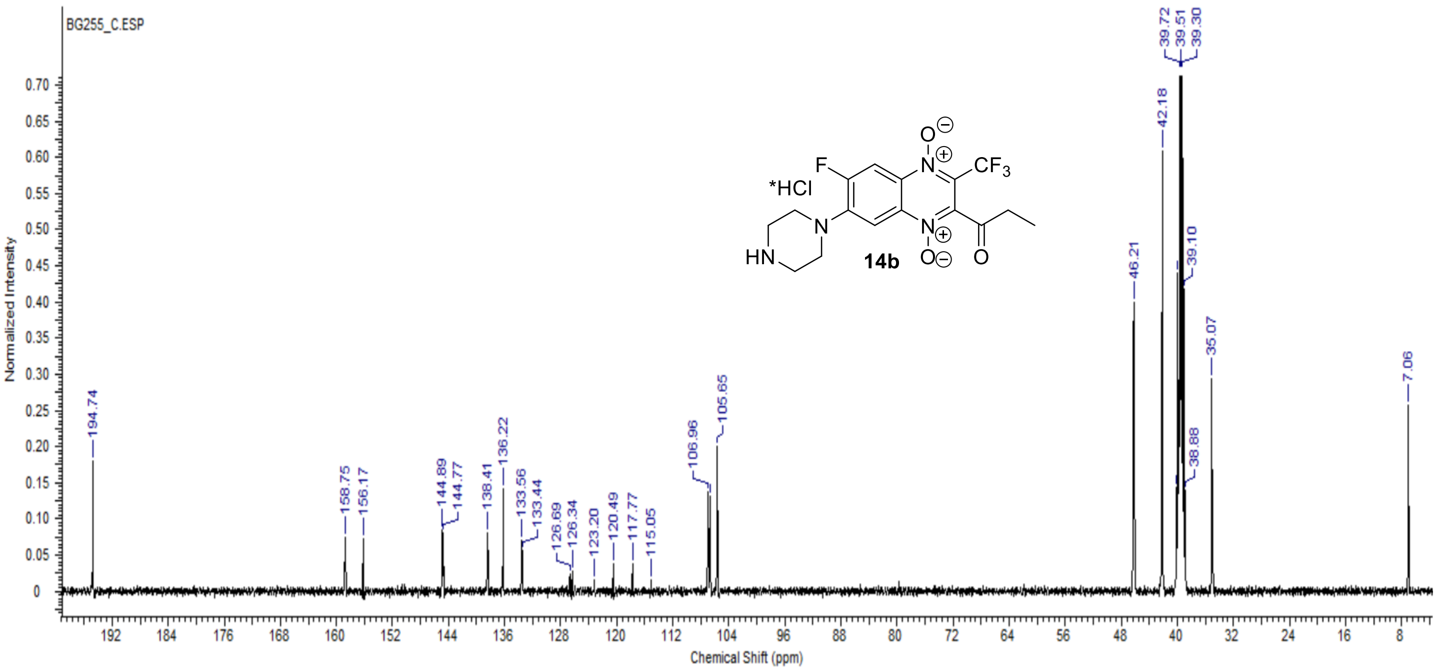

Figure S21. Copy of ^{1}H NMR spectrum of the derivative **14c**.

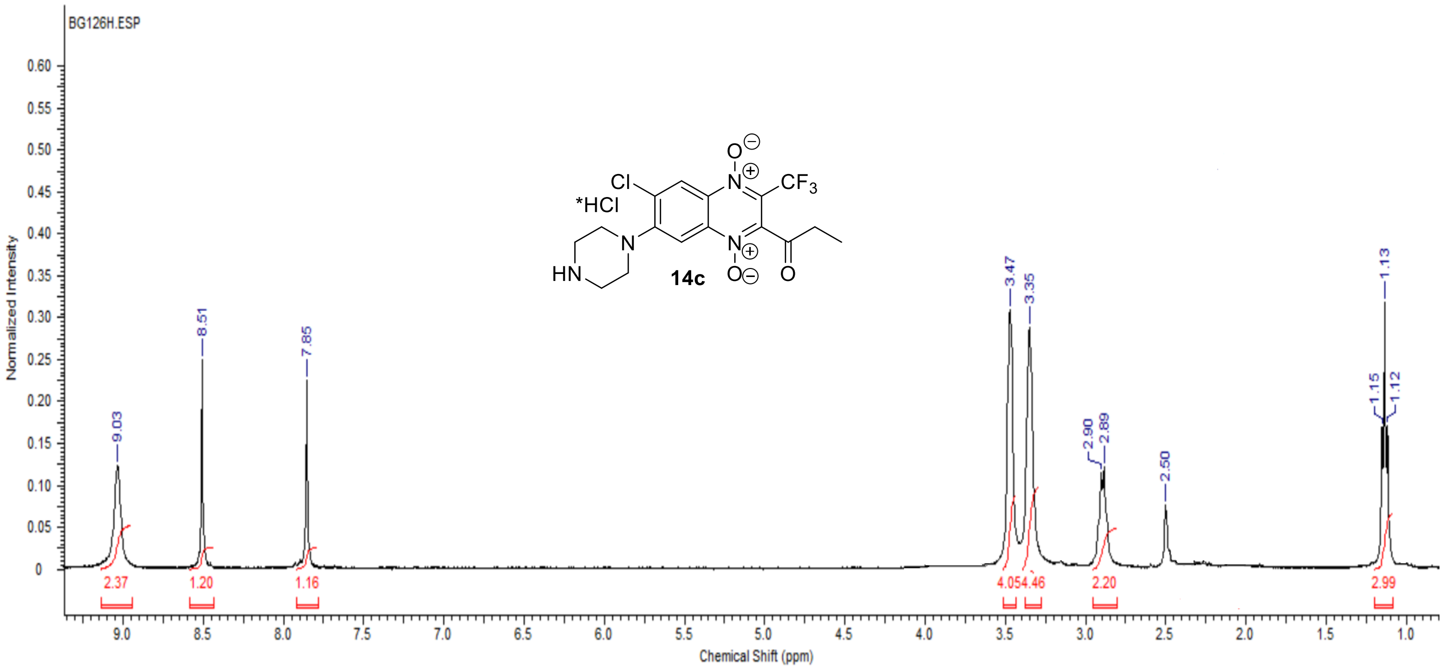

Figure S22. Copy of ^{13}C NMR spectrum of the derivative **14c**.

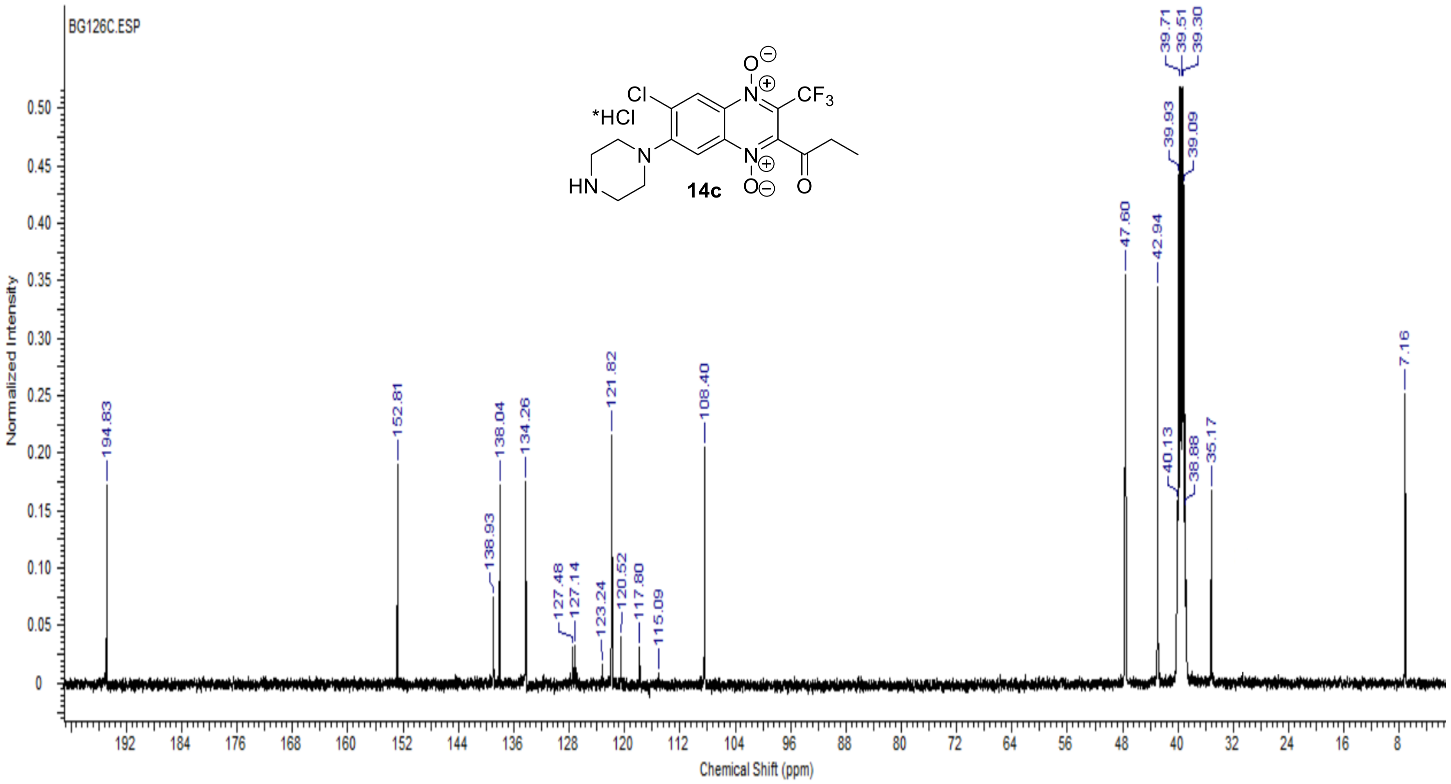

Figure S23. Copy of ^{1}H NMR spectrum of the derivative **15a**.

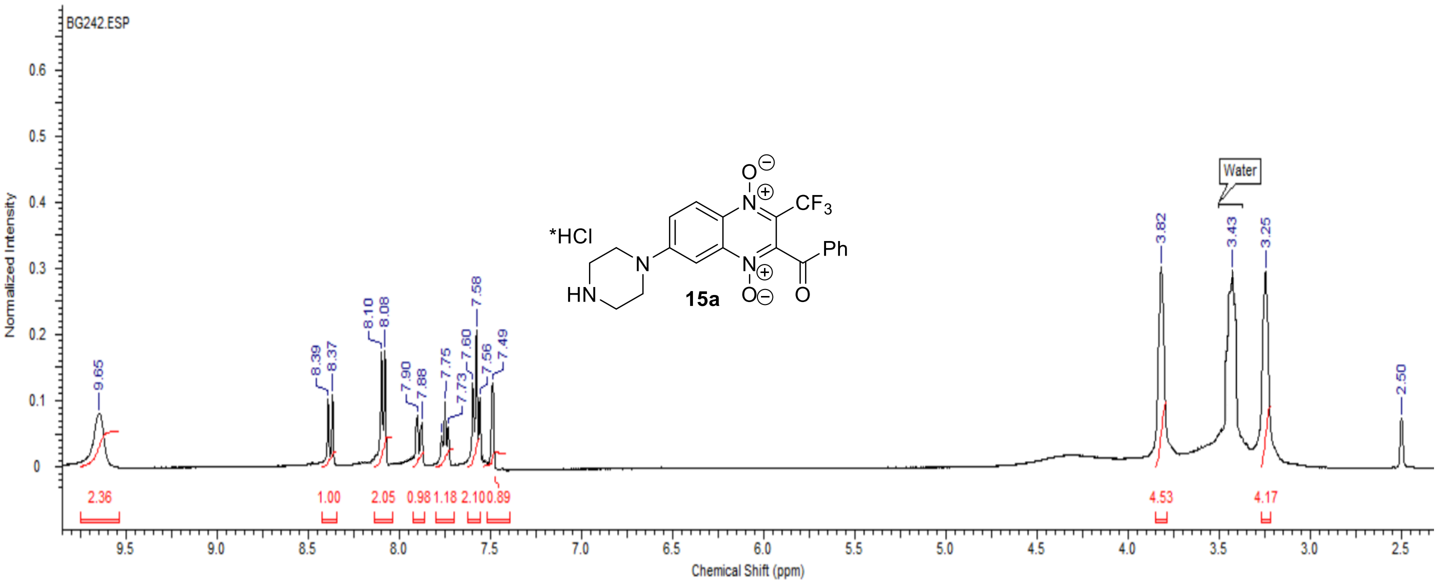

Figure S24. Copy of ^{13}C NMR spectrum of the derivative **15a**.

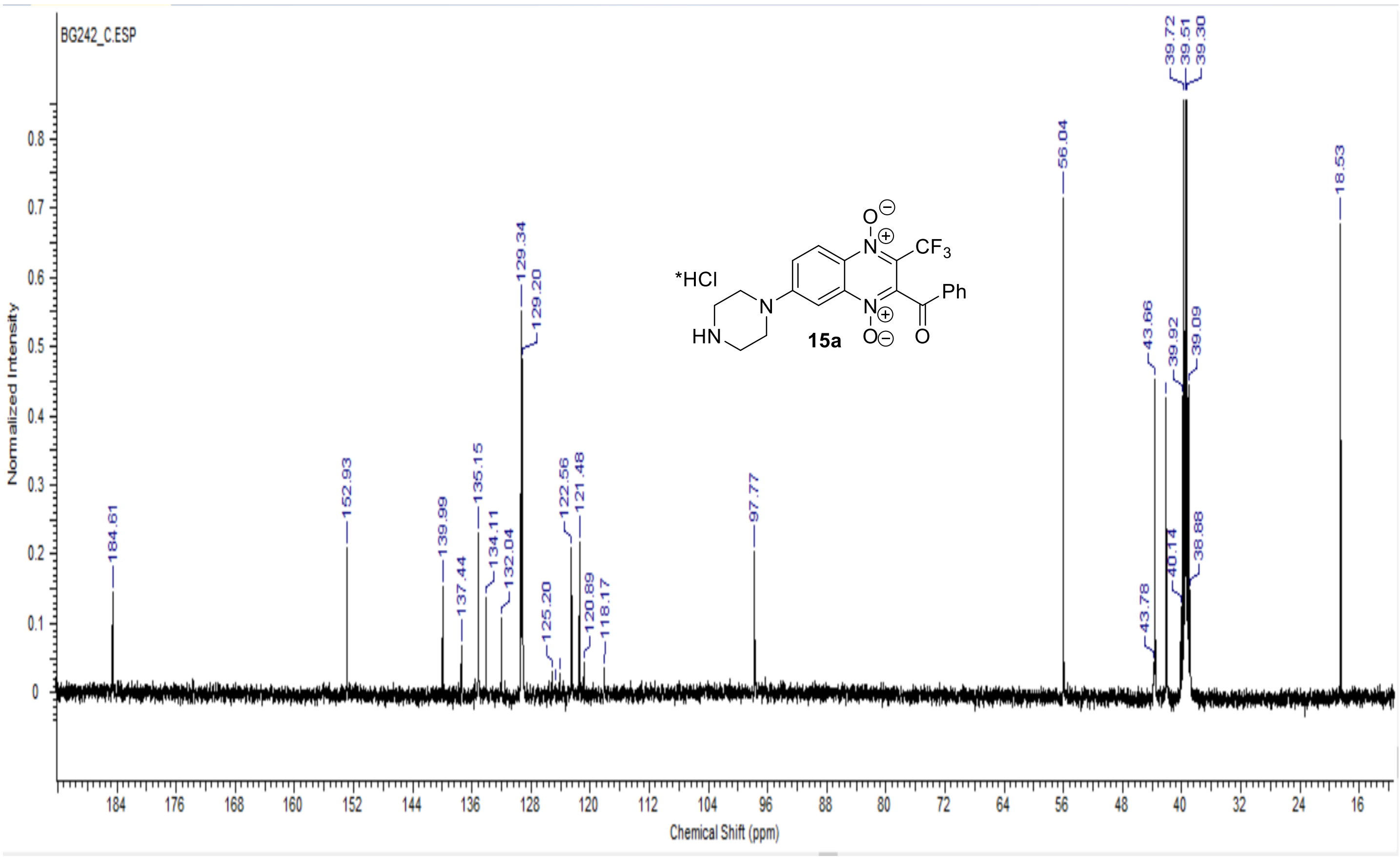

Figure S25. Copy of ^{1}H NMR spectrum of the derivative **15b**.

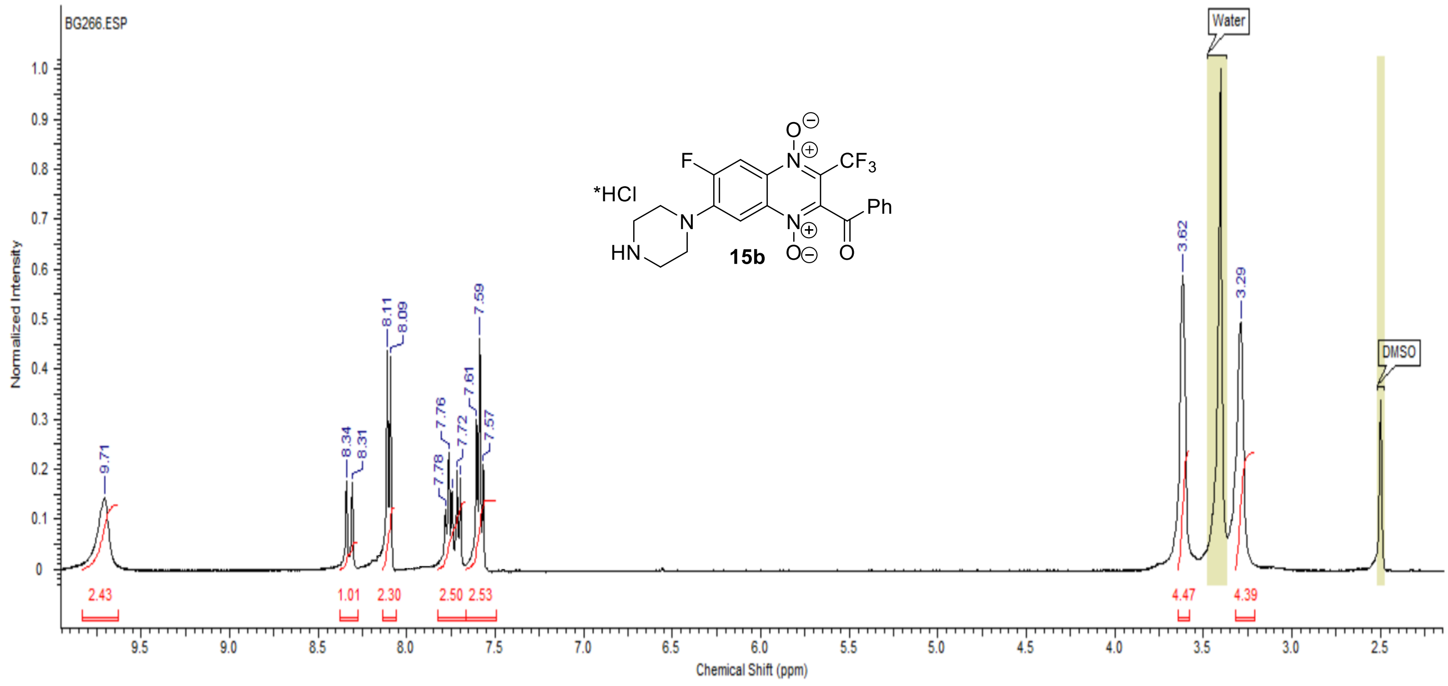

Figure S26. Copy of ^{13}C NMR spectrum of the derivative **15b**.

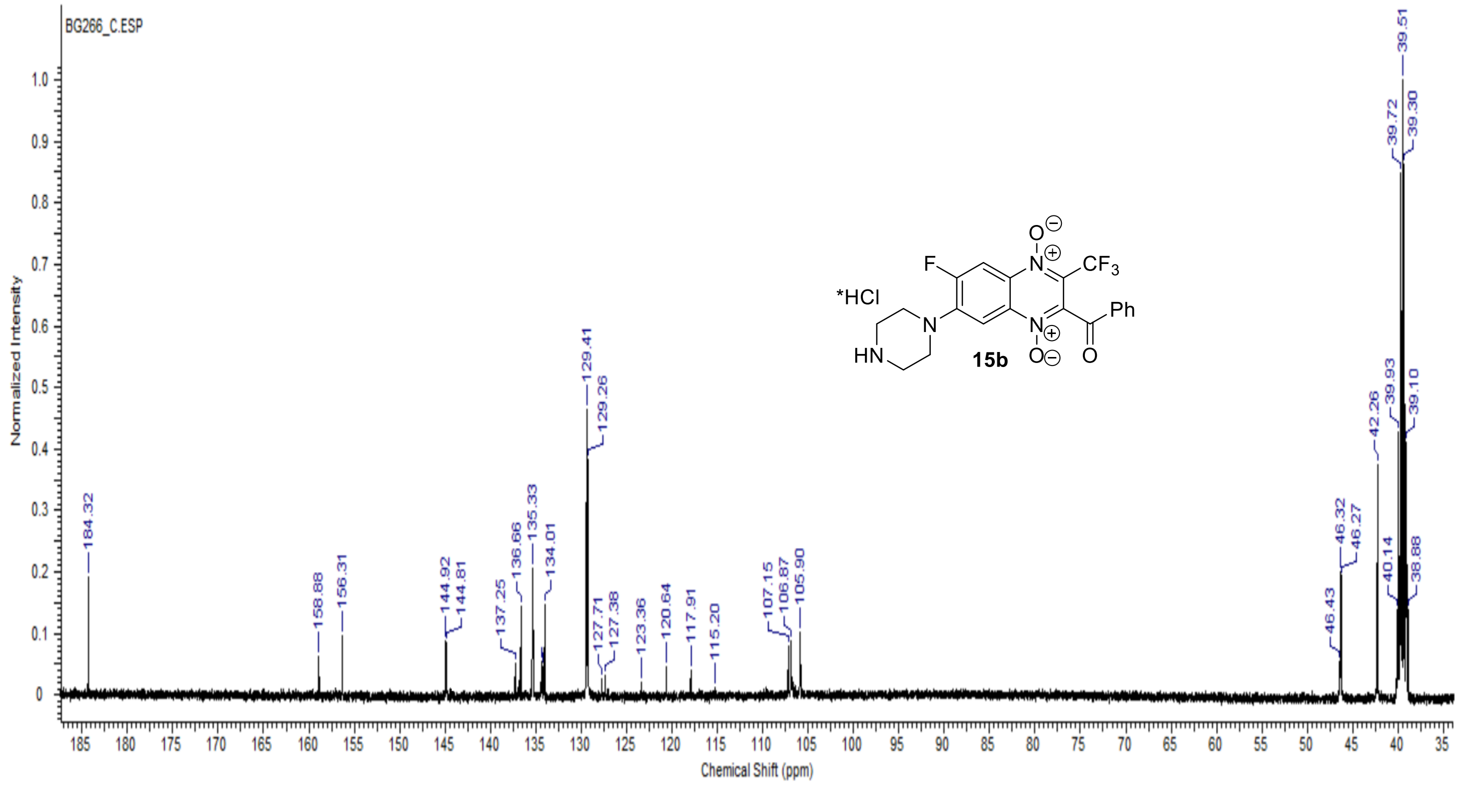

Figure S27. Copy of ^{1}H NMR spectrum of the derivative **15c**.

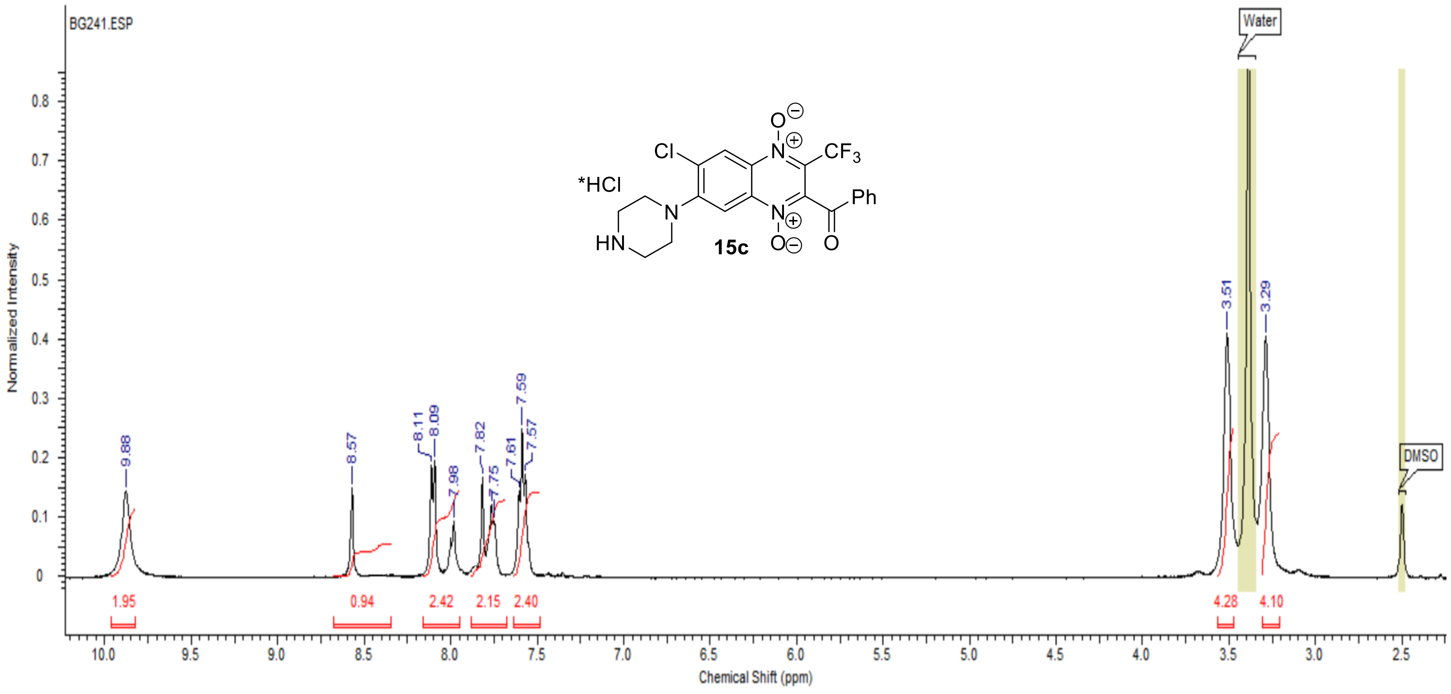

Figure S28. Copy of ^{13}C NMR spectrum of the derivative **15c**.

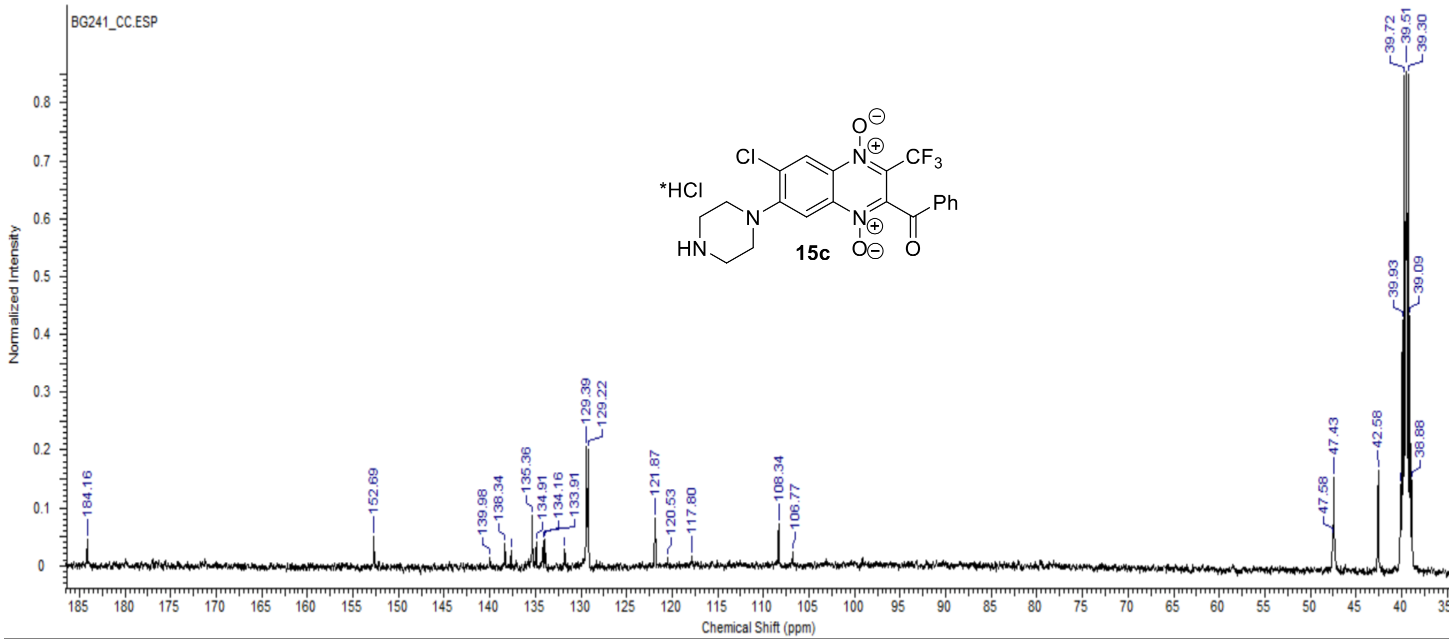

Figure S29. Copy of ^{1}H NMR spectrum of the derivative **15d**.

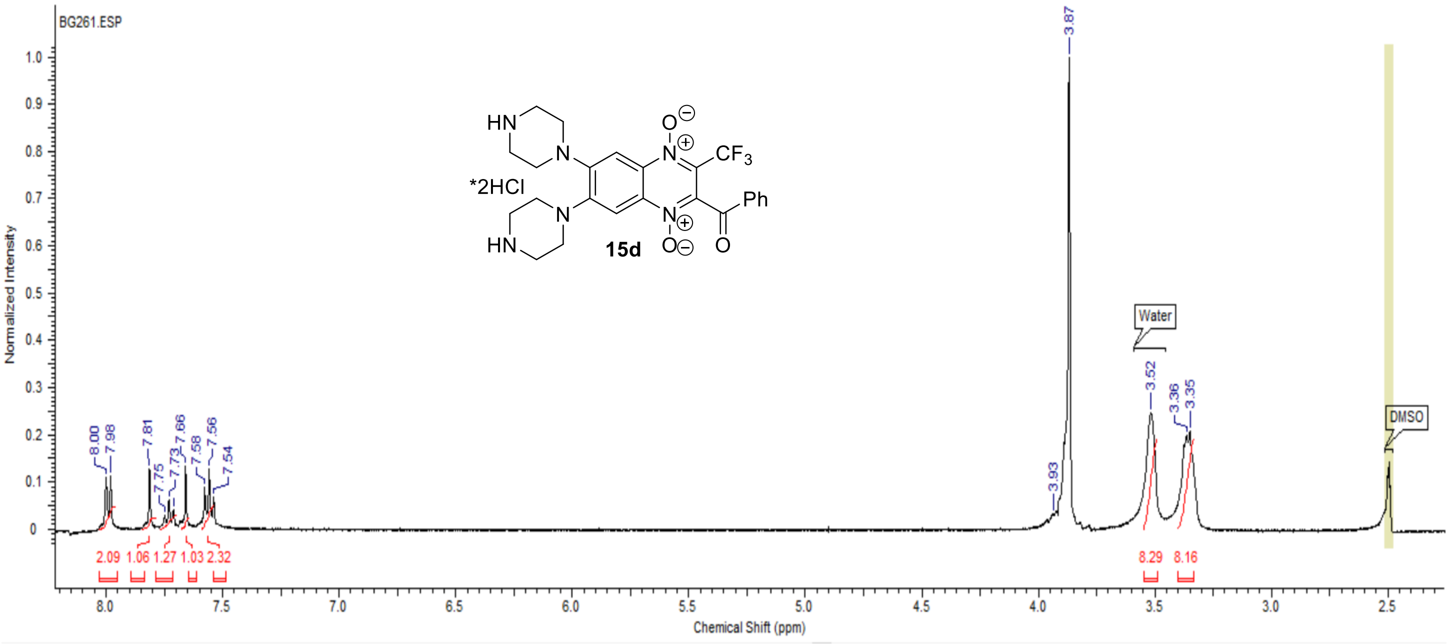

Figure S30. Copy of ^{13}C NMR spectrum of the derivative **15d**.

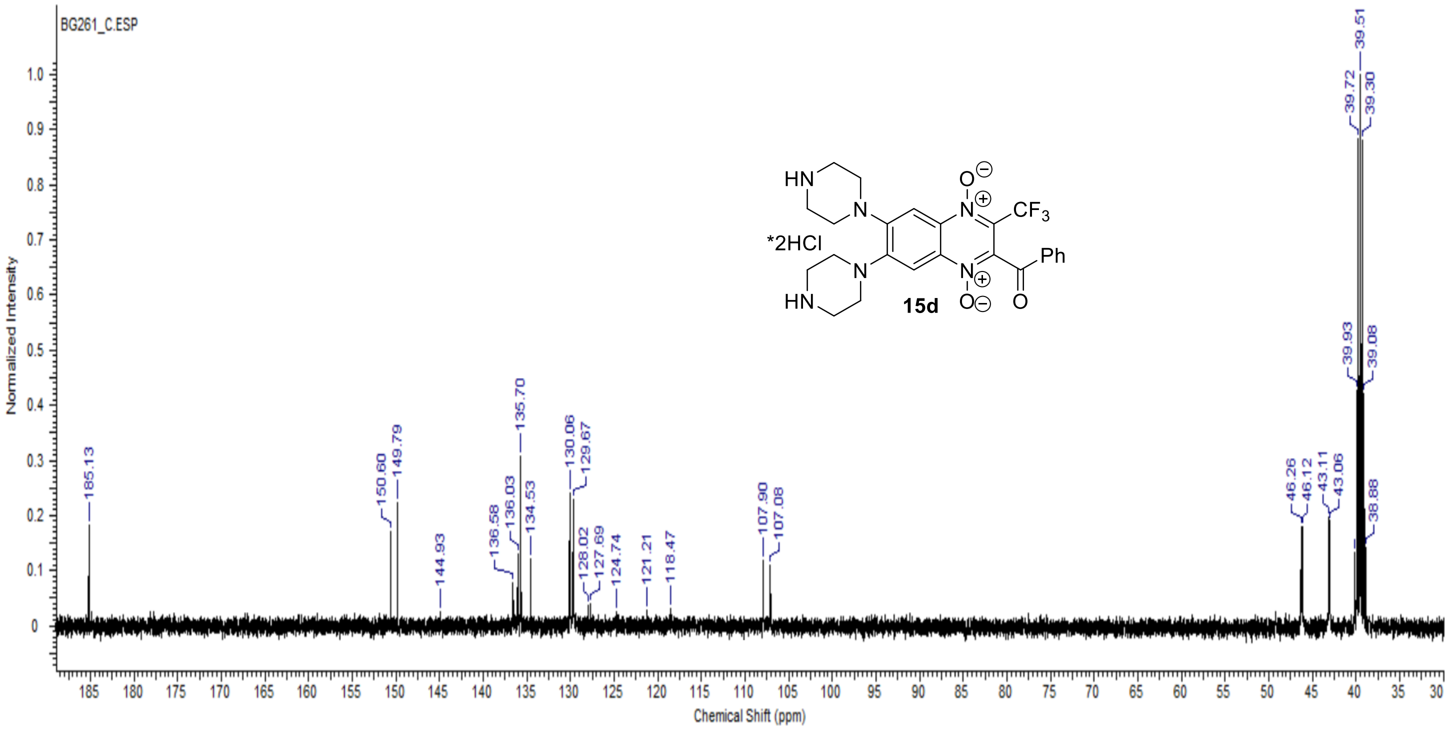

Figure S31. Copy of ^{1}H NMR spectrum of the derivative **16a**.

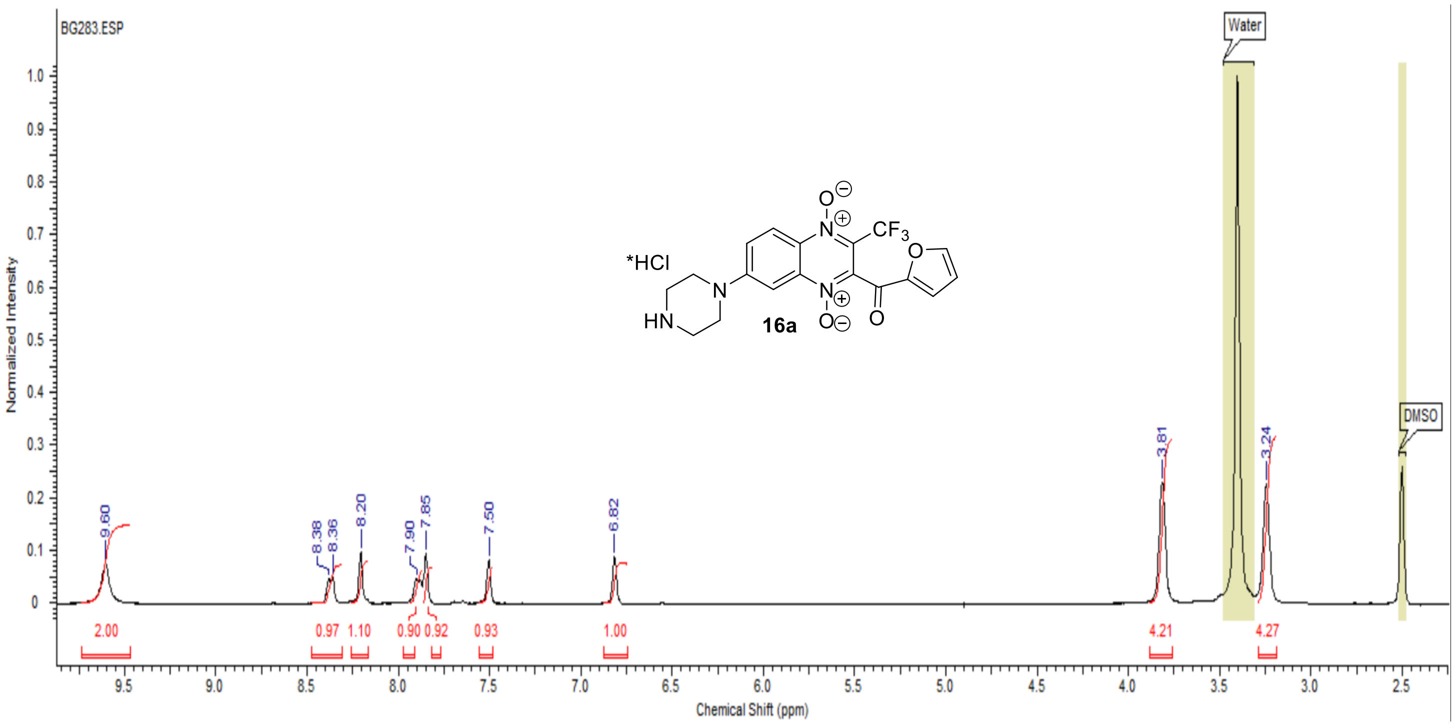

Figure S32. Copy of ^{13}C NMR spectrum of the derivative **16a**.

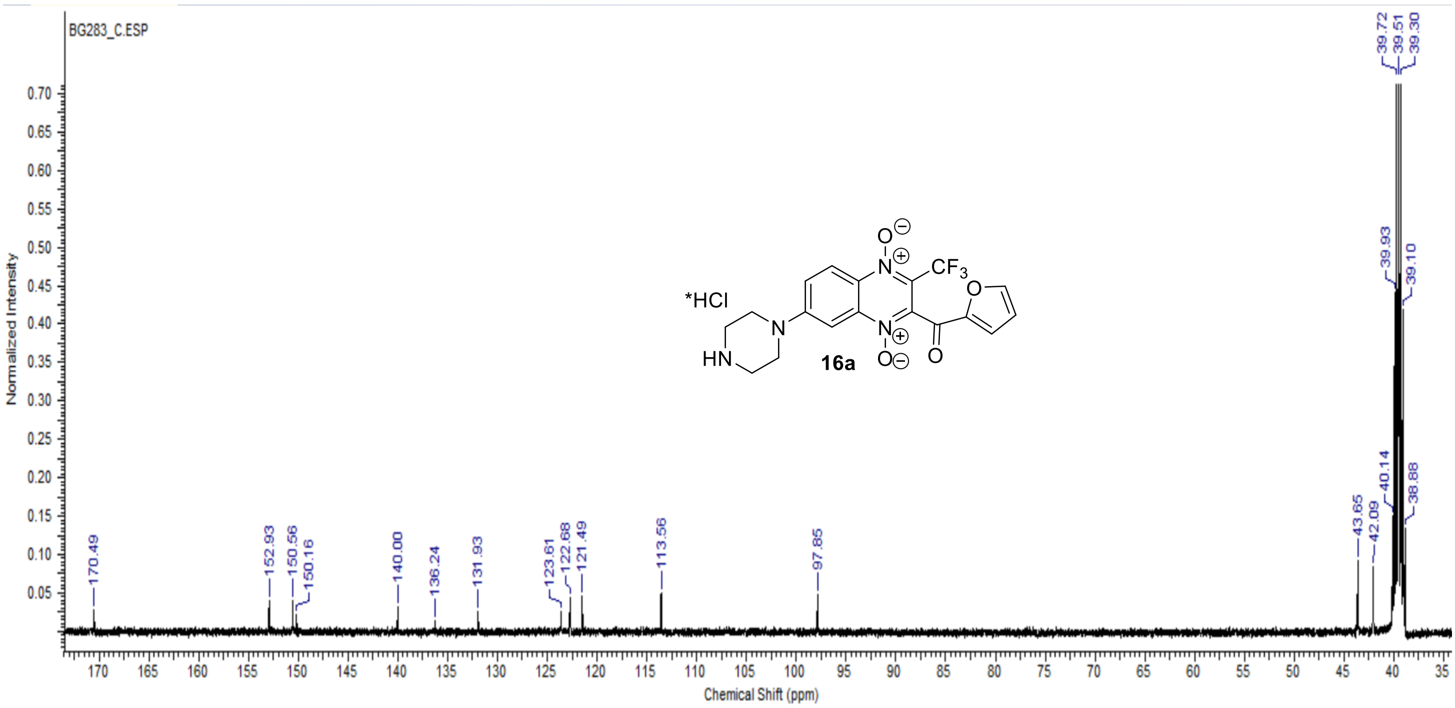

Figure S33. Copy of ^{1}H NMR spectrum of the derivative **16b**.

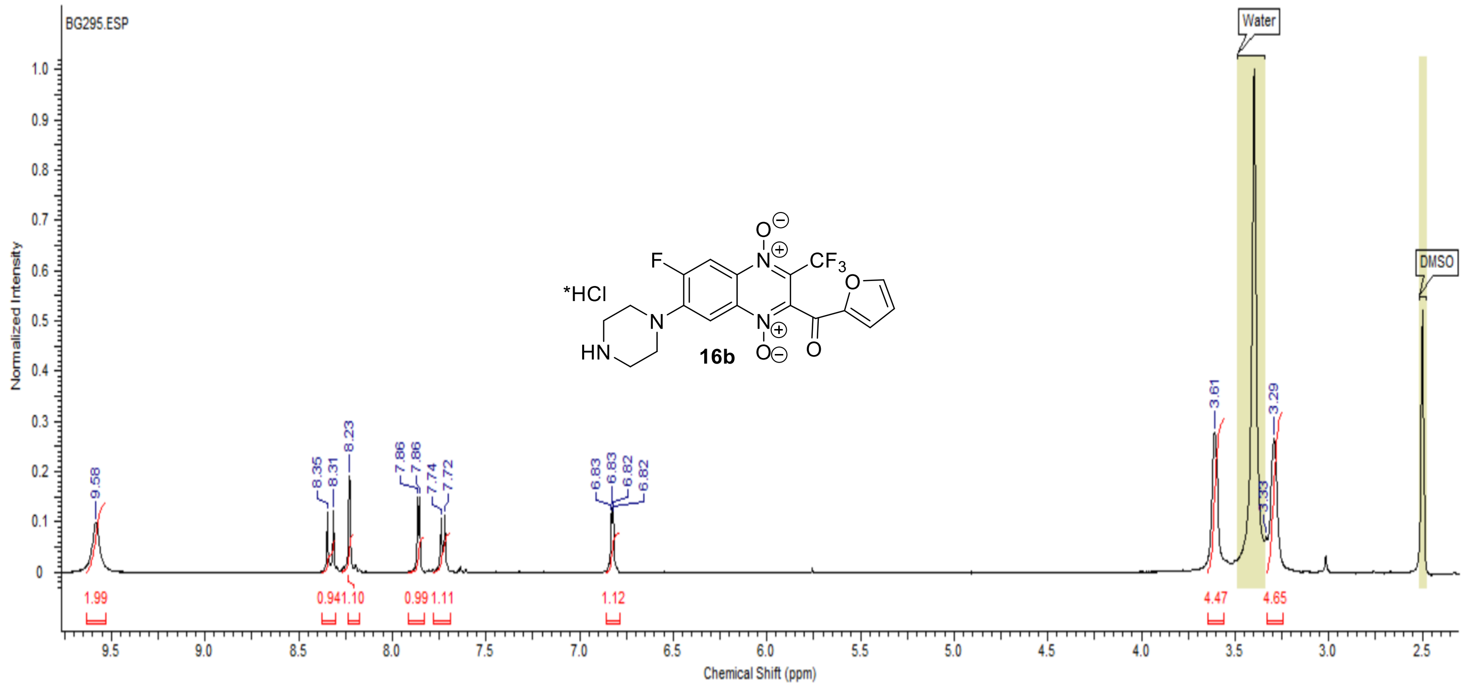

Figure S34. Copy of ^{13}C NMR spectrum of the derivative **16b**.

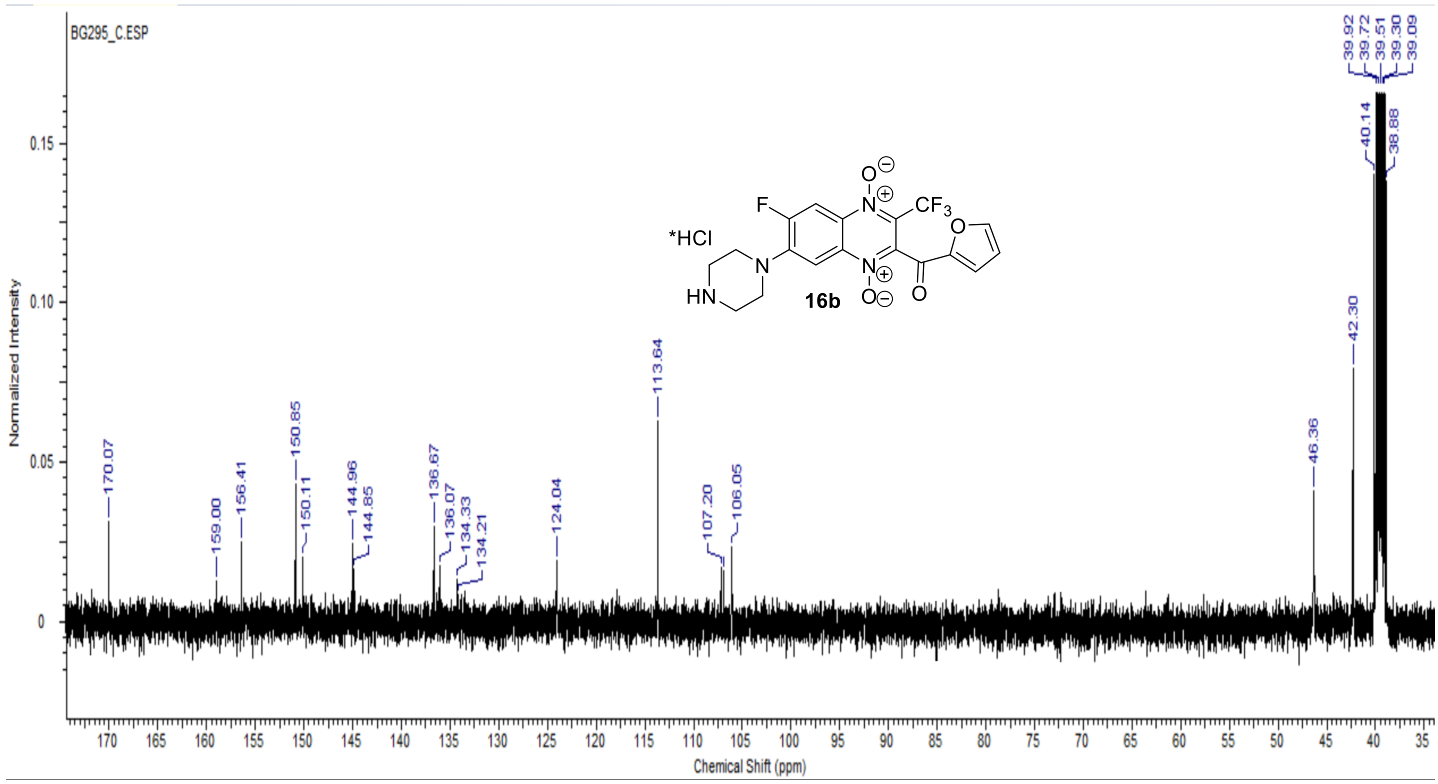

Figure S35. Copy of ^{1}H NMR spectrum of the derivative **16c**.

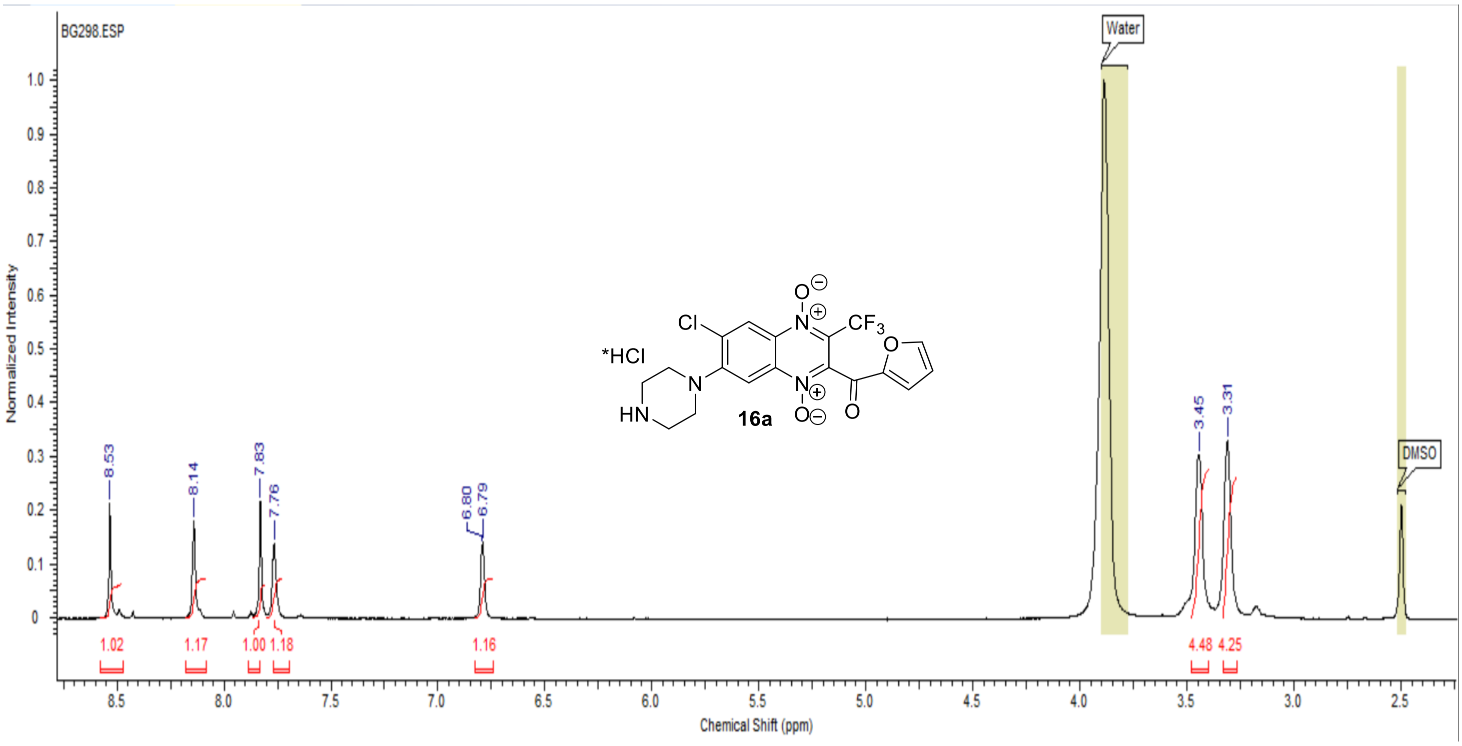

Figure S36. Copy of ^{13}C NMR spectrum of the derivative **16c**.

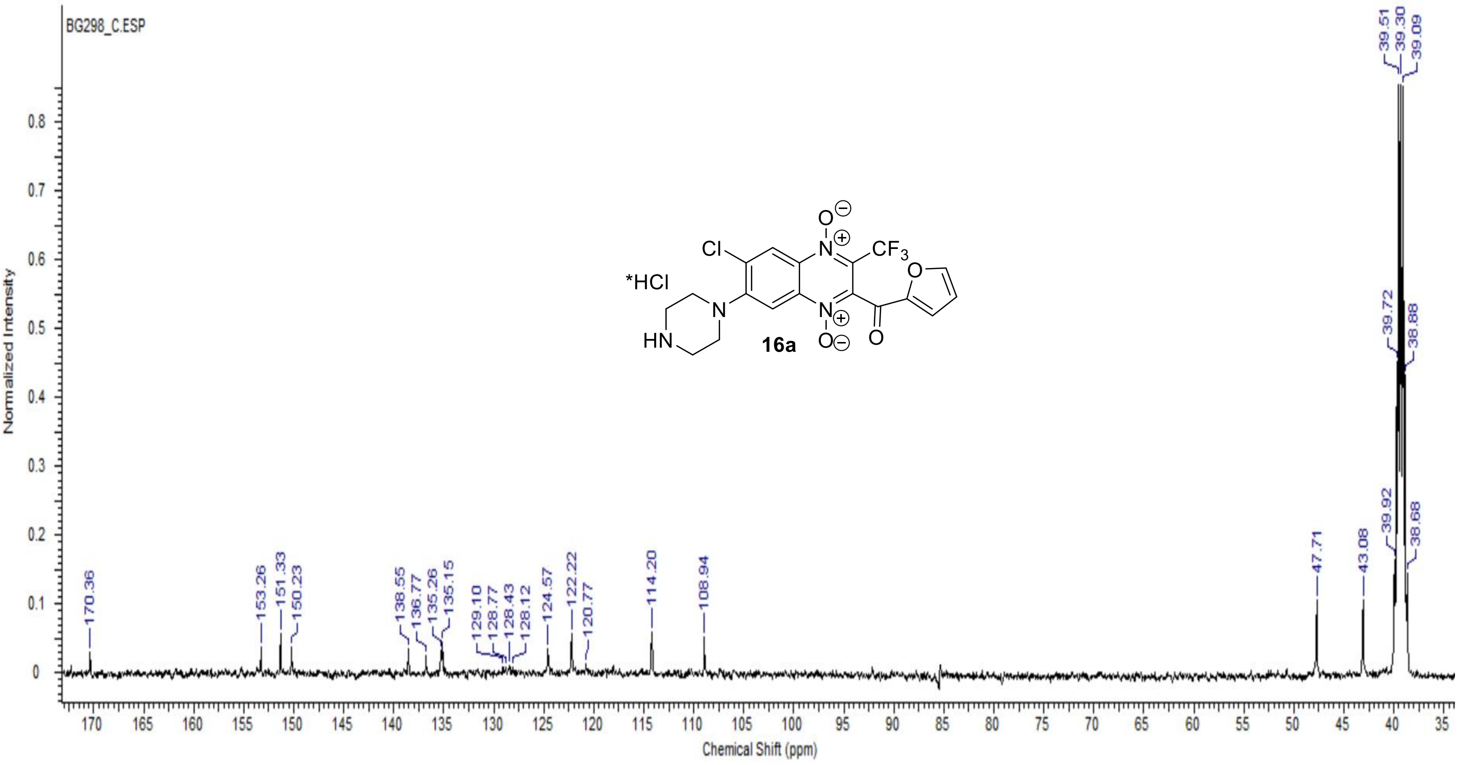

Figure S37. Copy of ^{1}H NMR spectrum of the derivative **16d**.

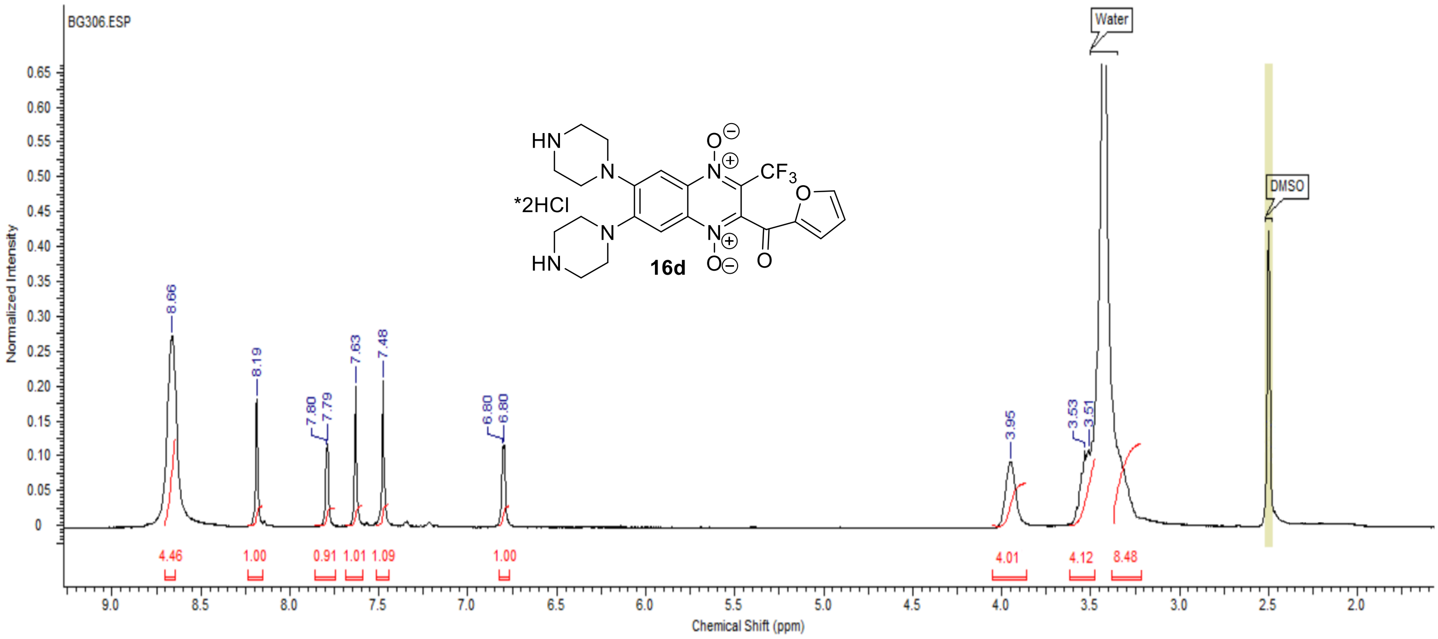

Figure S38. Copy of ^{13}C NMR spectrum of the derivative **16d**.

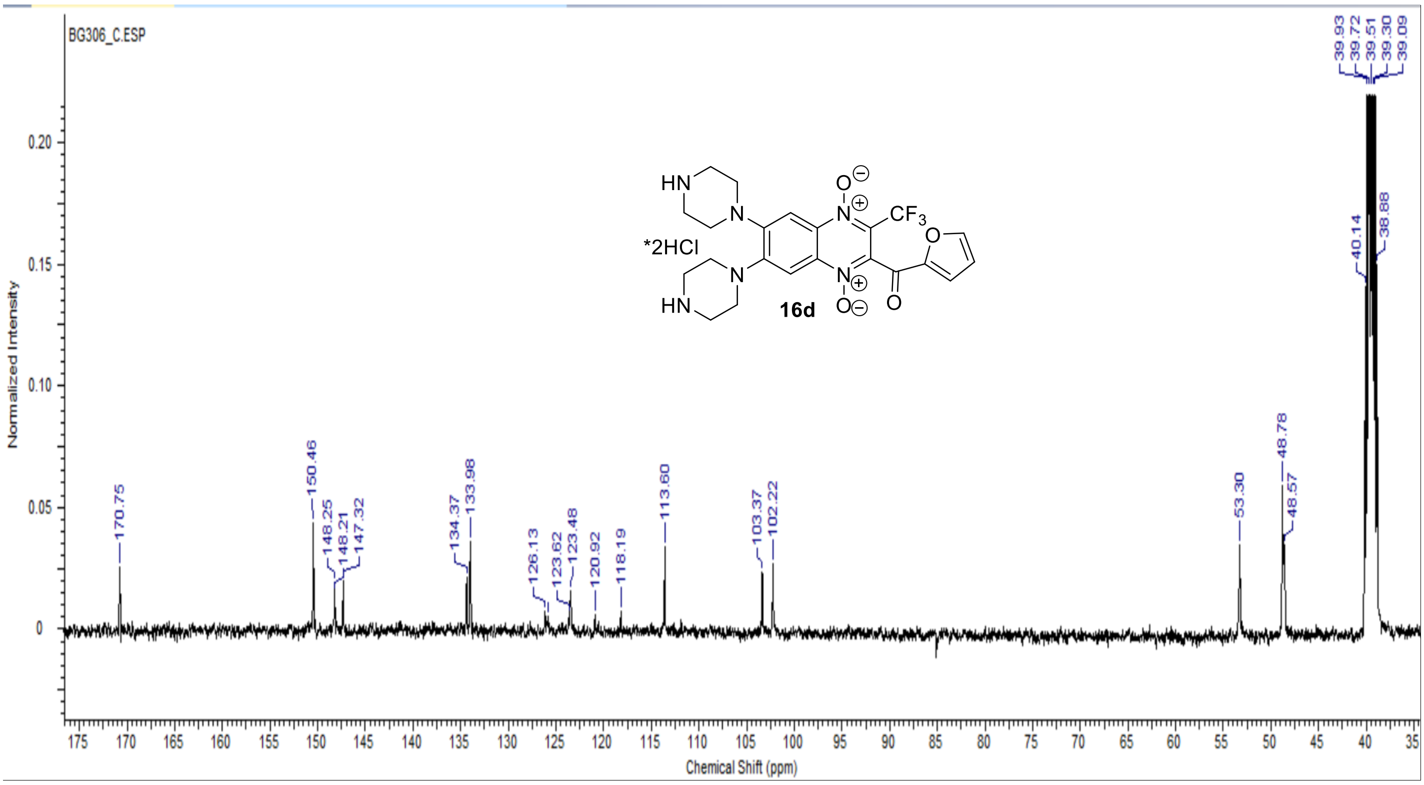

Figure S39. Copy of ^{1}H NMR spectrum of the derivative **17a**.

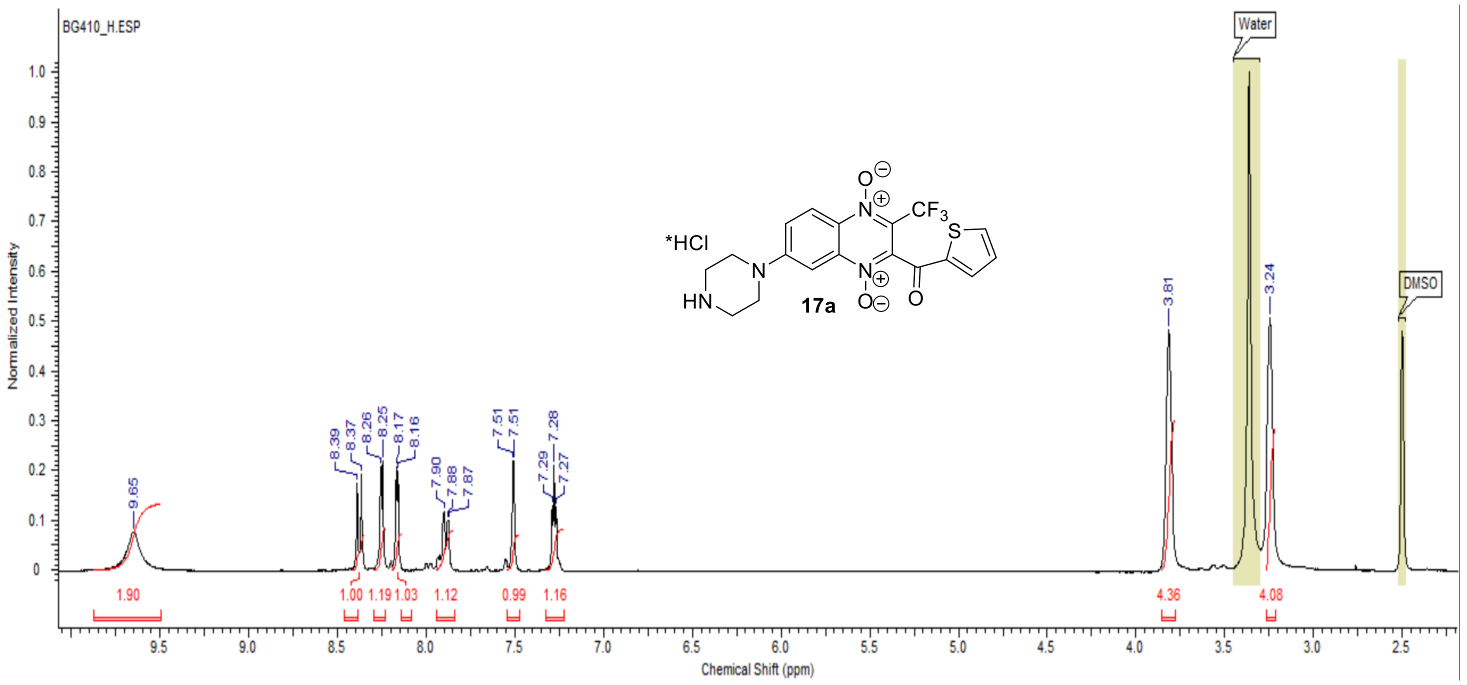

Figure S40. Copy of ^{13}C NMR spectrum of the derivative **17a**.

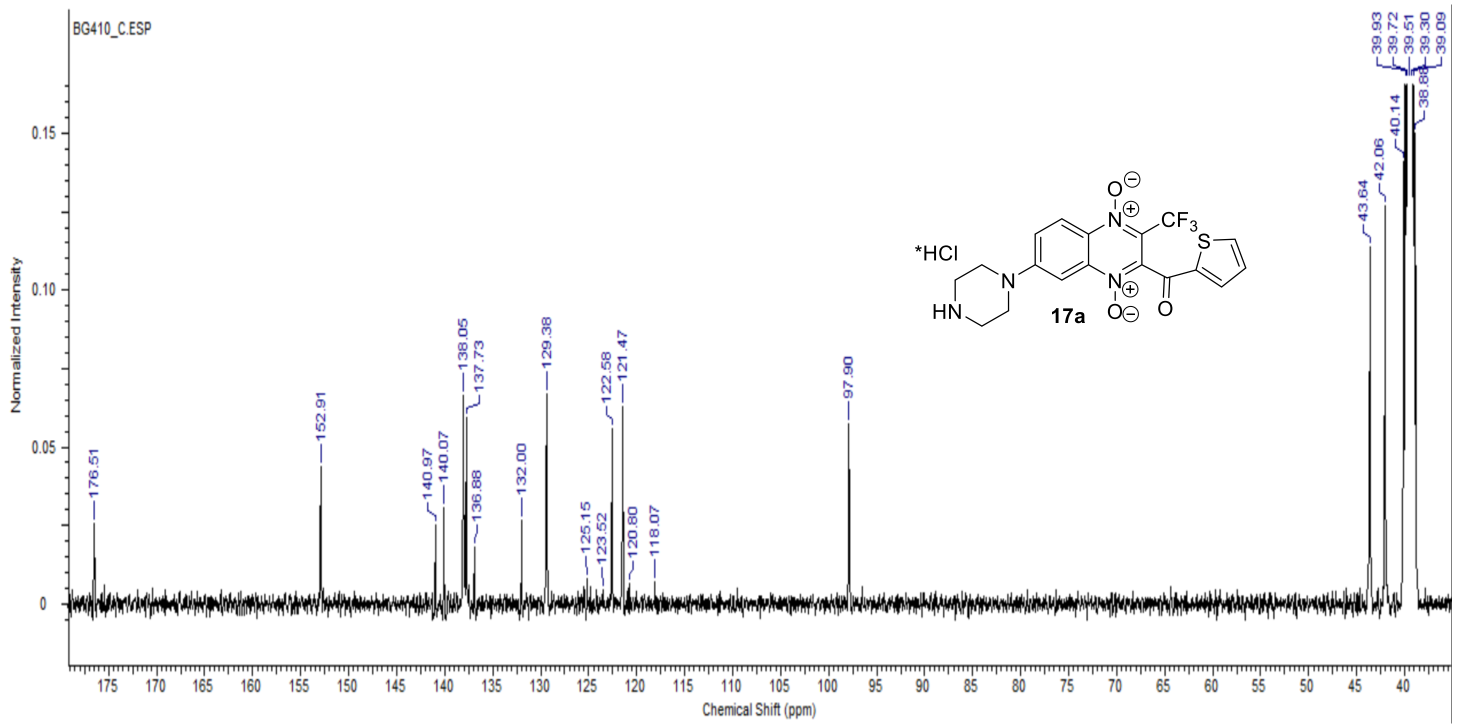

Figure S41. Copy of ^{1}H NMR spectrum of the derivative **17b**.

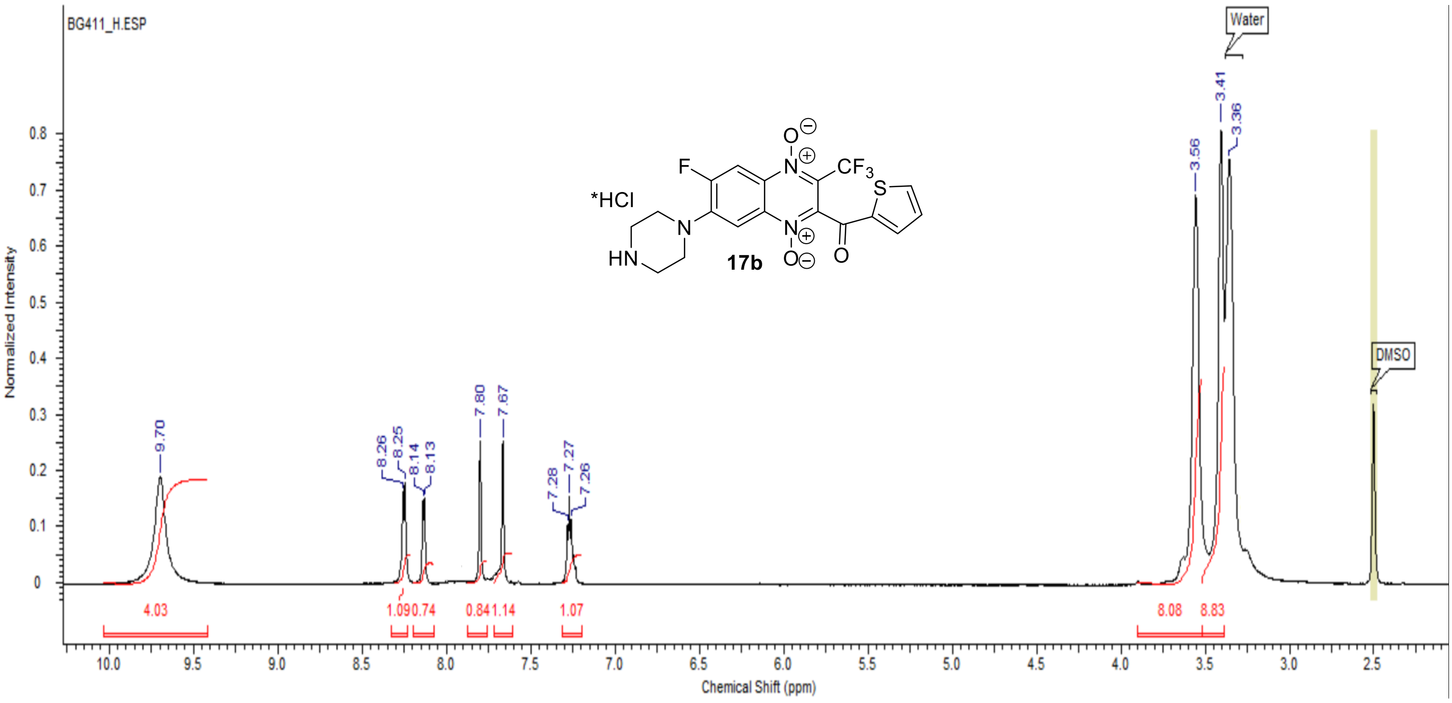

Figure S42. Copy of ^{13}C NMR spectrum of the derivative **17b**.

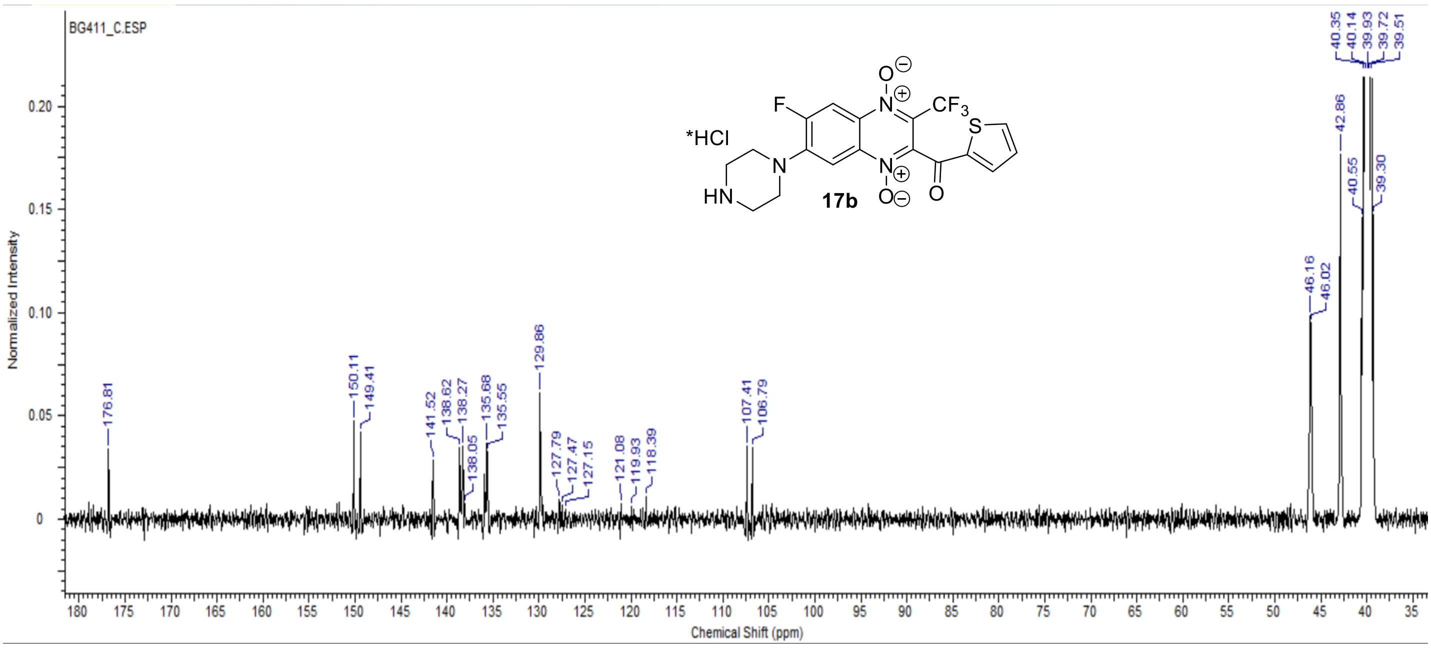

Figure S43. Copy of ^{1}H NMR spectrum of the derivative **17c**.

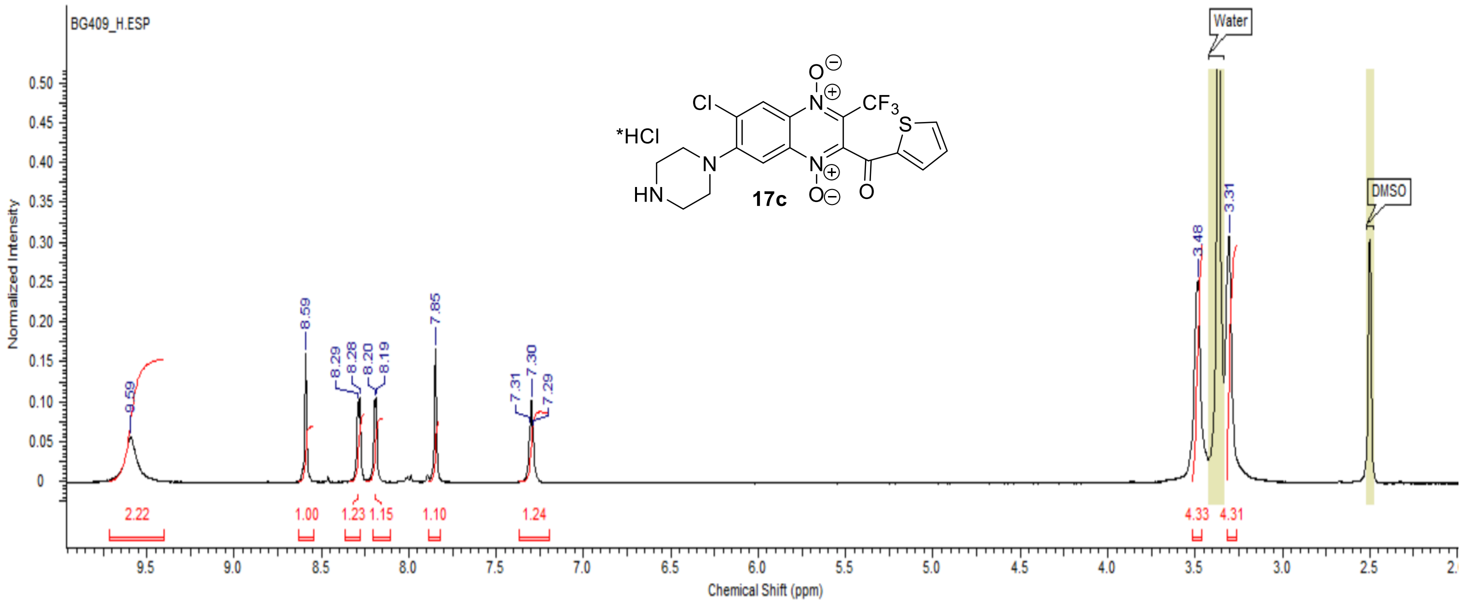

Figure S44. Copy of ^{13}C NMR spectrum of the derivative **17c**.

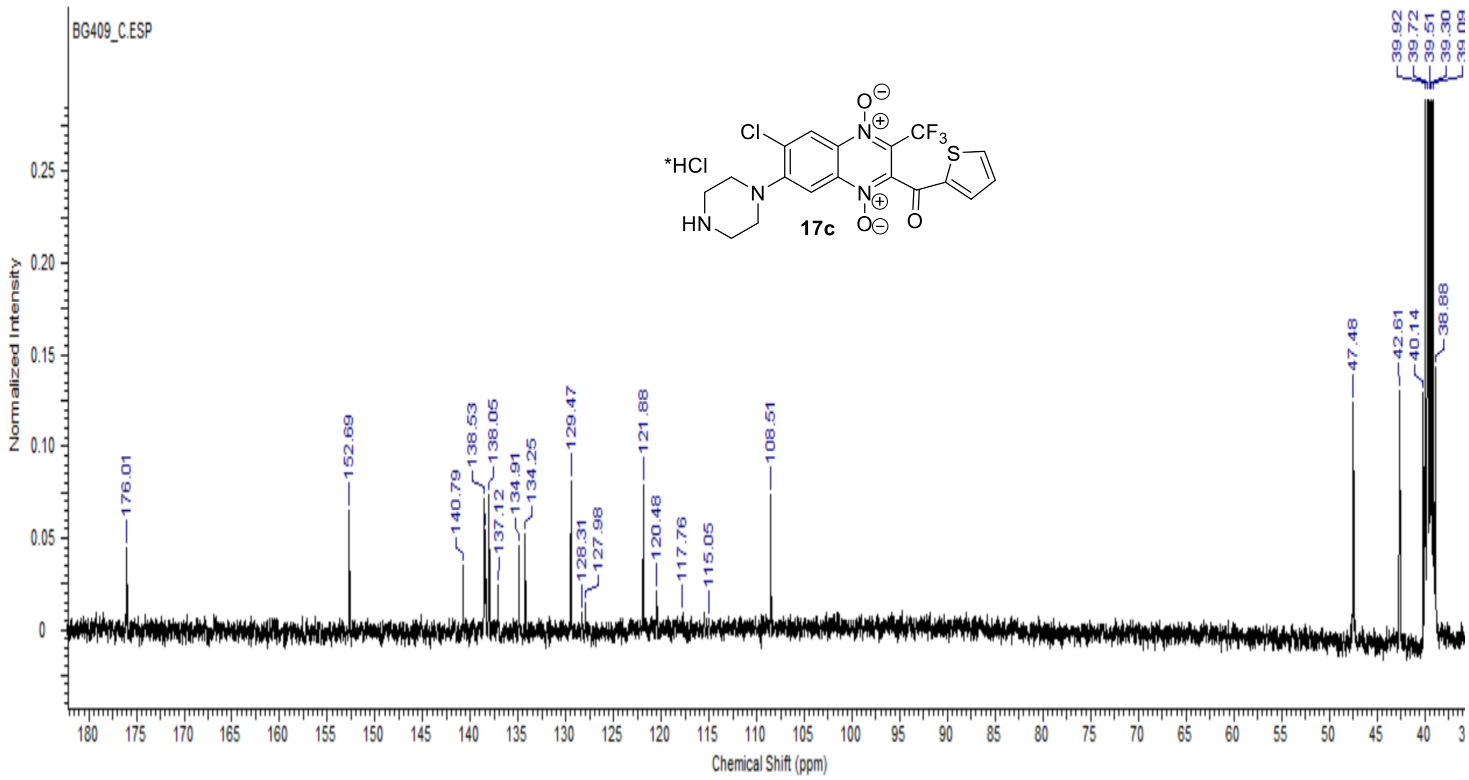

Figure S45. Copy of ^{1}H NMR spectrum of the derivative **17d**.

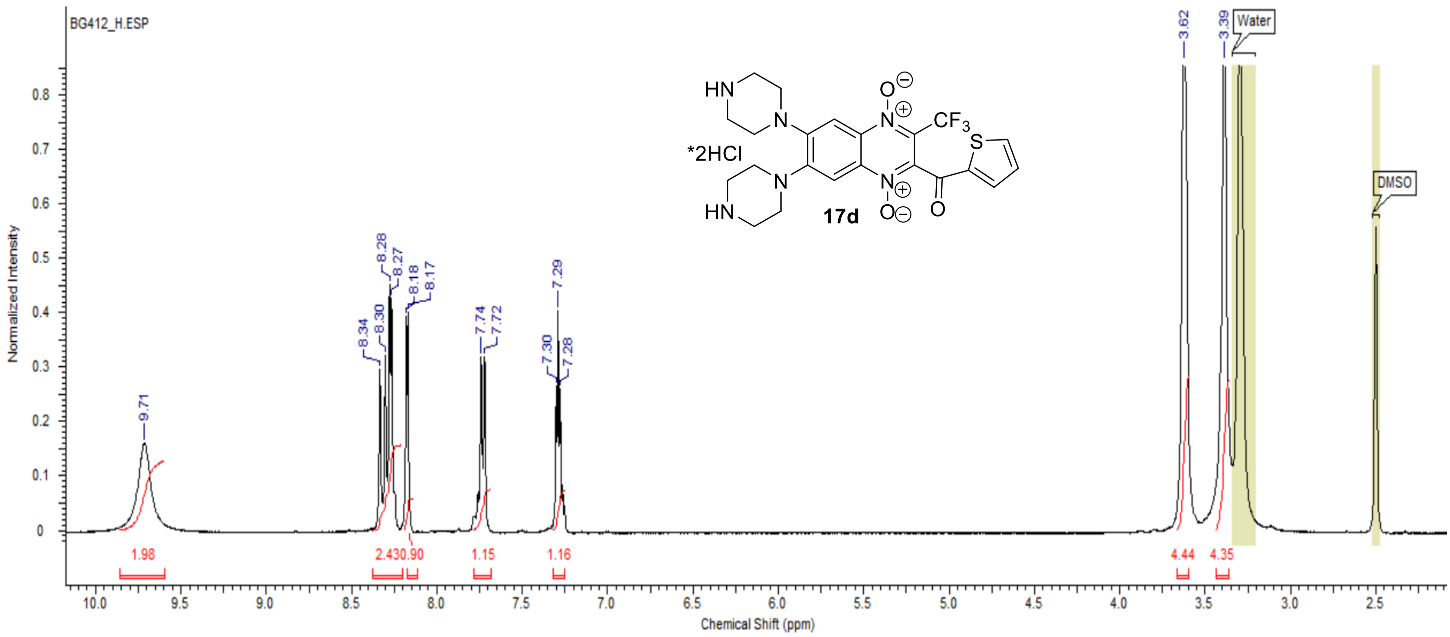

Figure S46. Copy of ^{13}C NMR spectrum of the derivative **17d**.

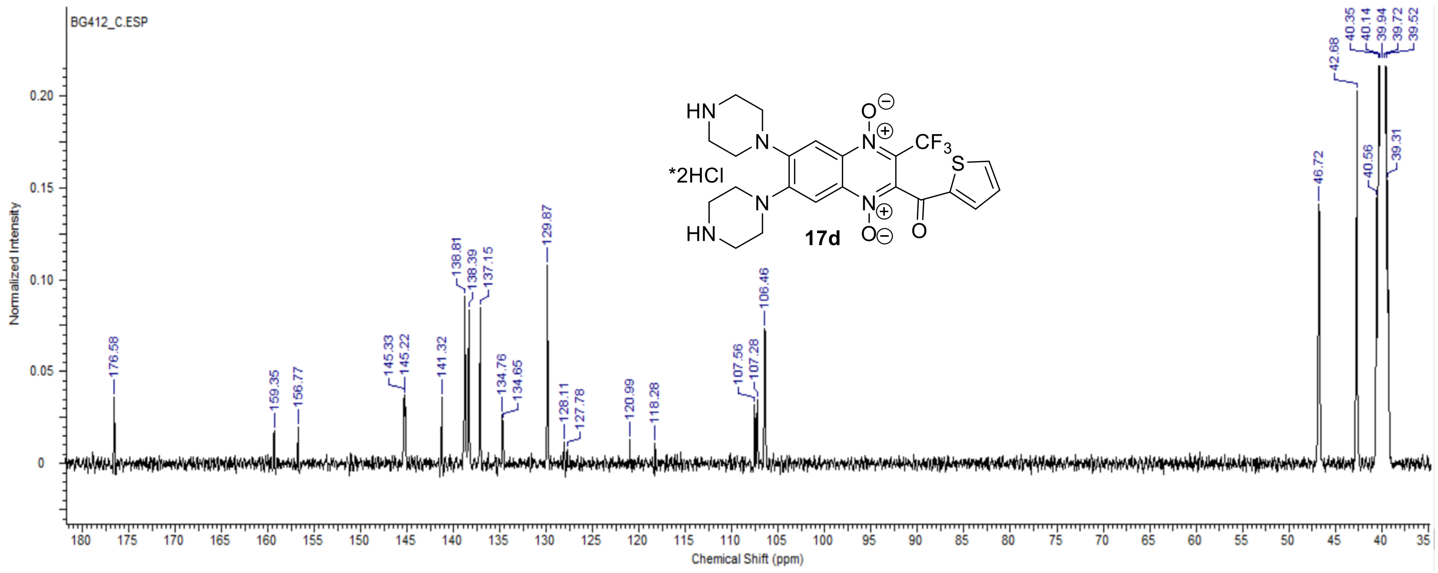

Figure S47. Copy of ^{1}H NMR spectrum of the derivative **18a**.

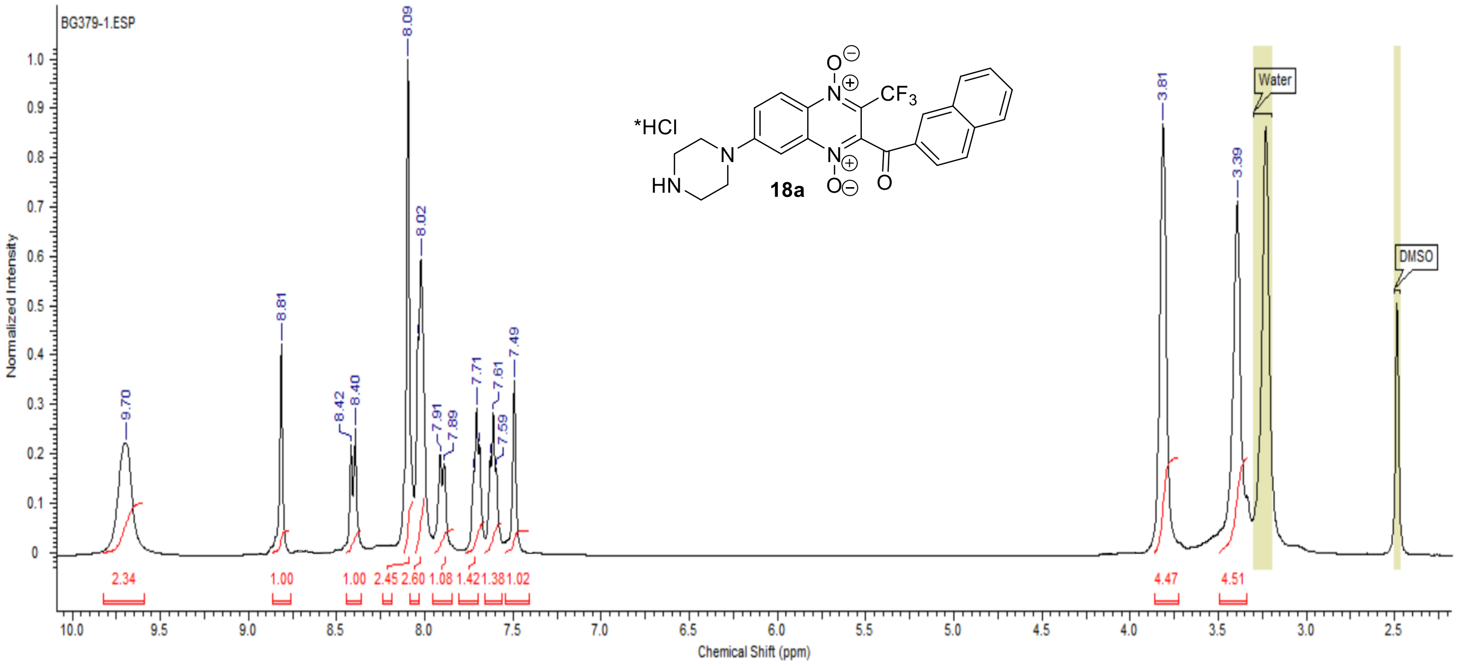

Figure S48. Copy of ^{13}C NMR spectrum of the derivative **18a**.

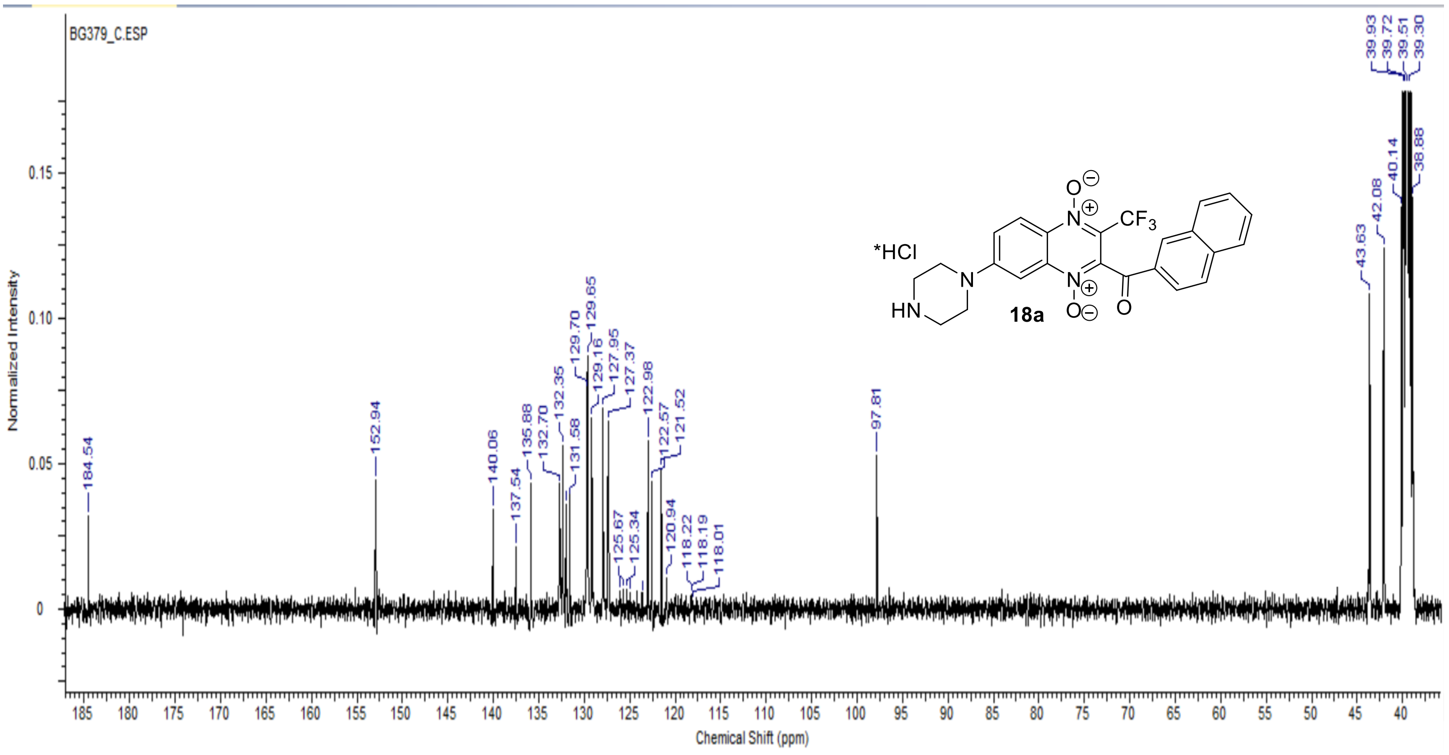

Figure S49. Copy of ^{1}H NMR spectrum of the derivative **18b**.

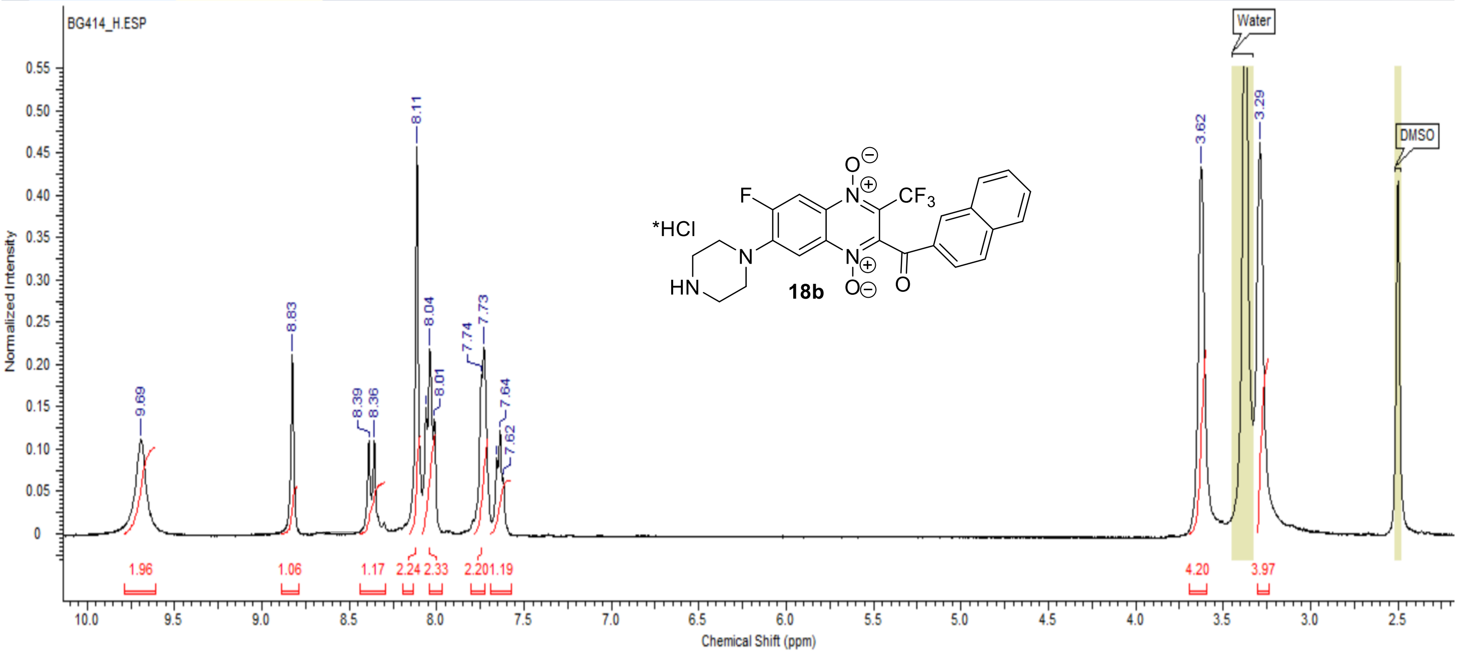

Figure S50. Copy of ^{13}C NMR spectrum of the derivative **18b**.

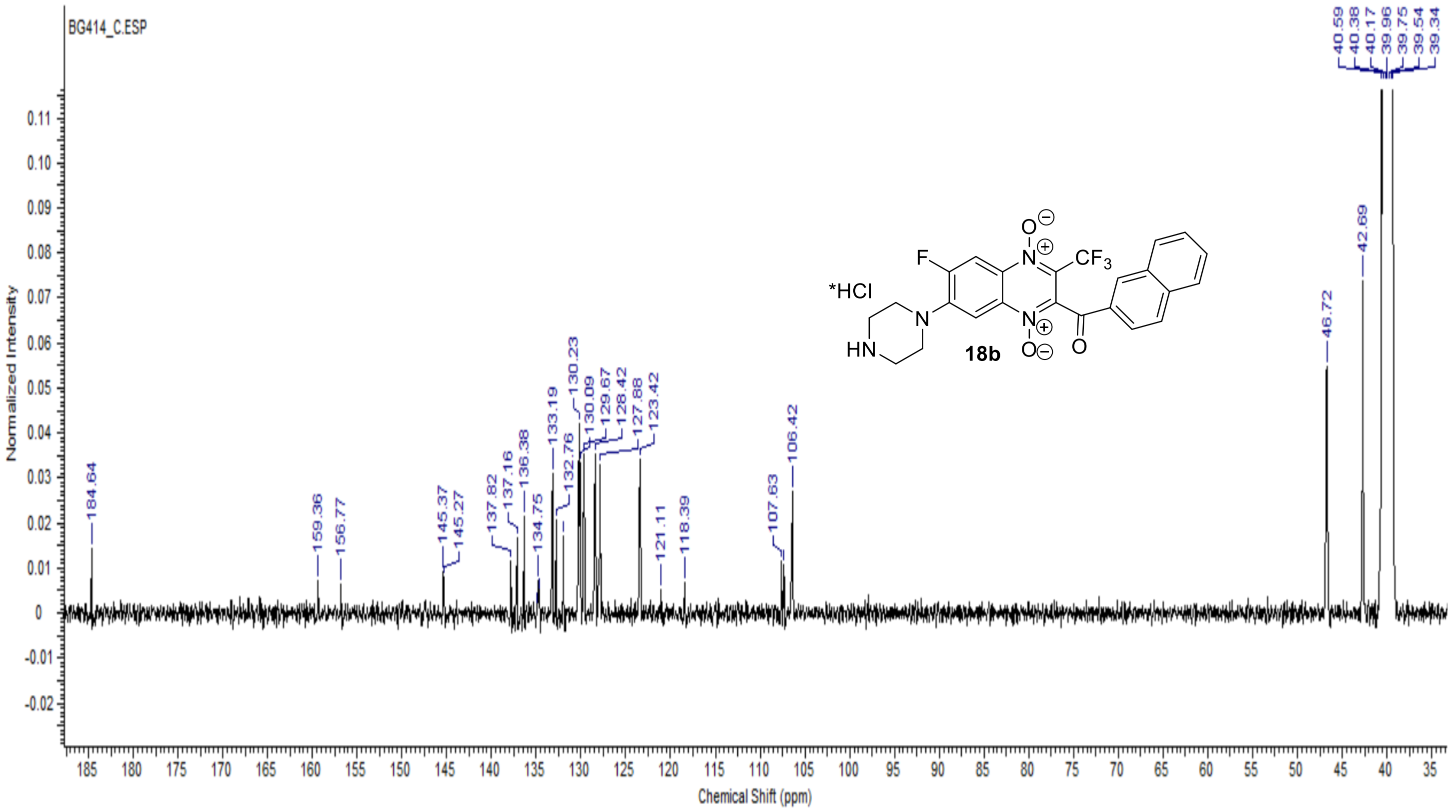

Figure S51. Copy of ^{1}H NMR spectrum of the derivative **18c**.

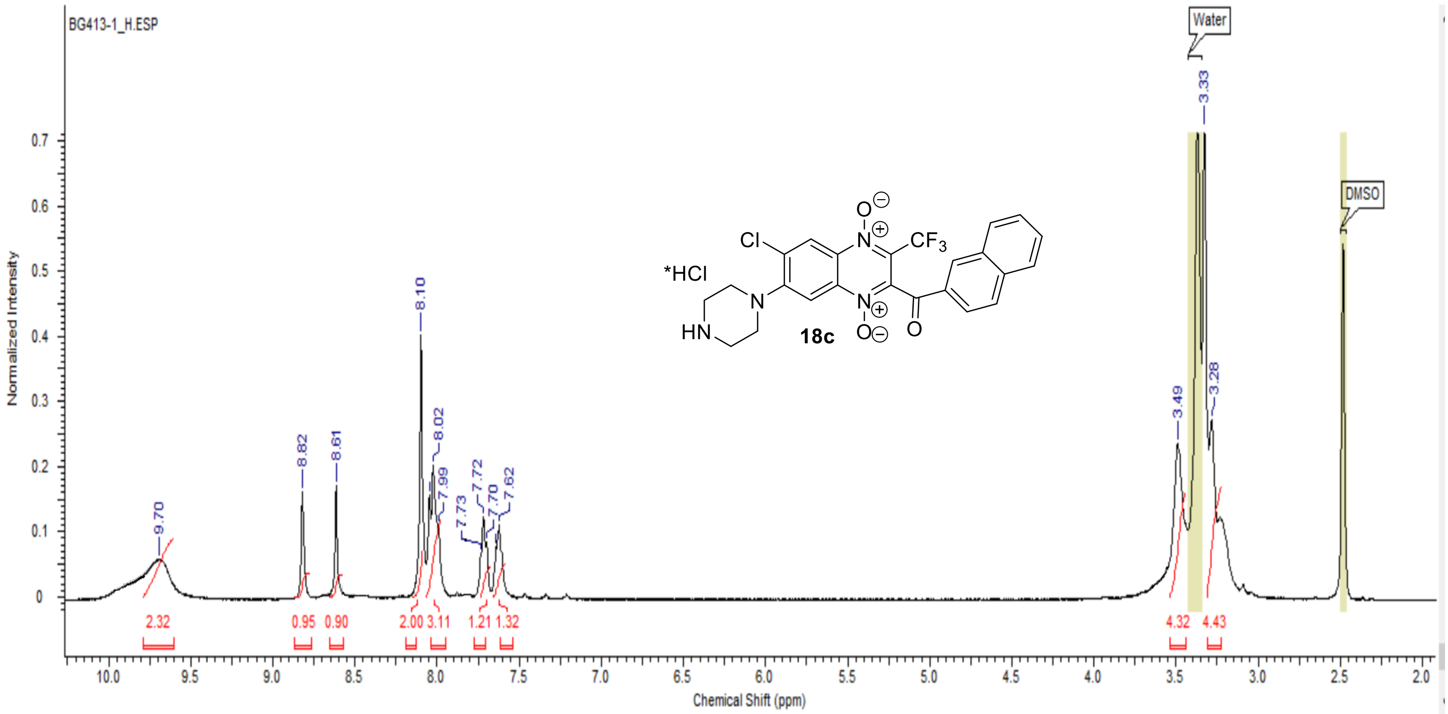

Figure S52. Copy of ^{13}C NMR spectrum of the derivative **18c**.

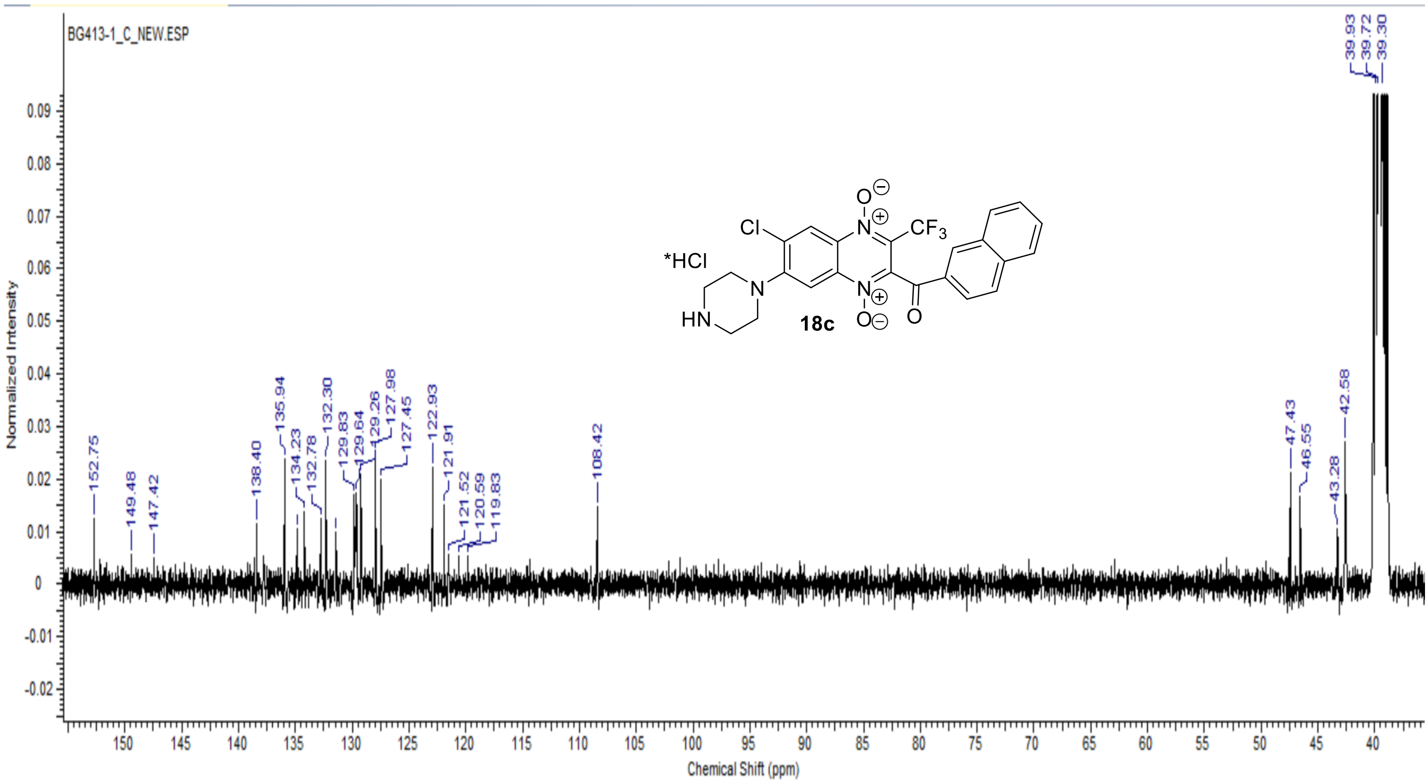

Figure S53. Copy of ^{1}H NMR spectrum of the derivative **18d**.

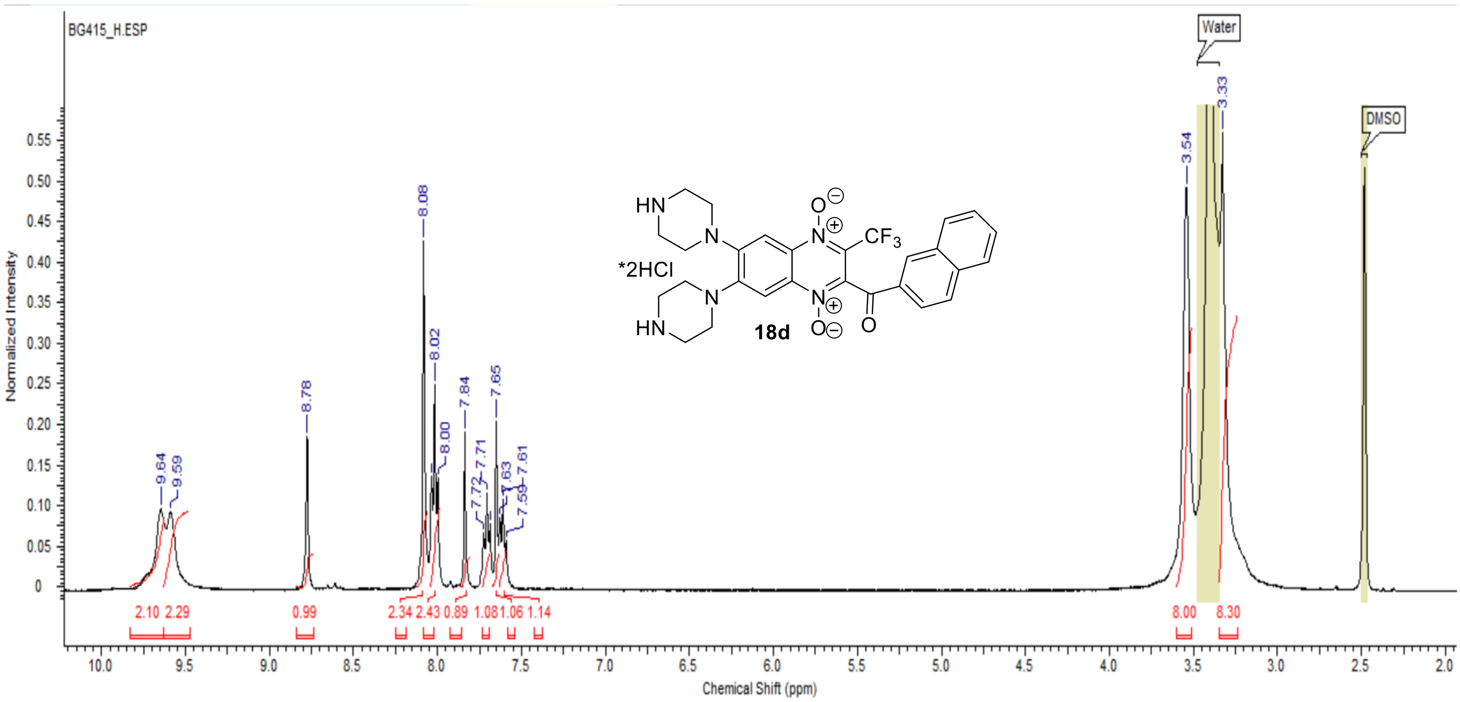

Figure S54. Copy of ^{13}C NMR spectrum of the derivative **18d**.

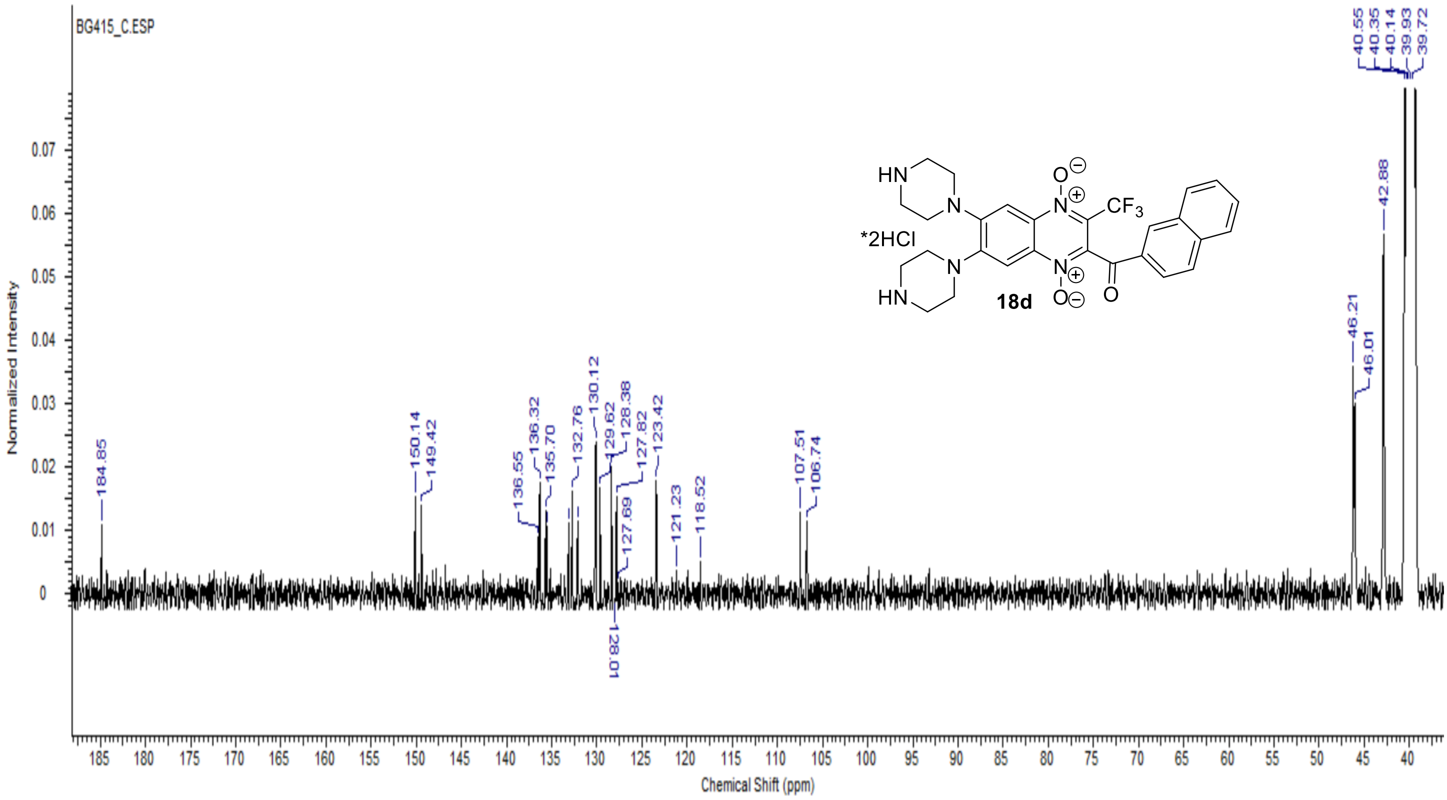

Figure S55. Copy of ^{1}H NMR spectrum of the derivative **7d**.

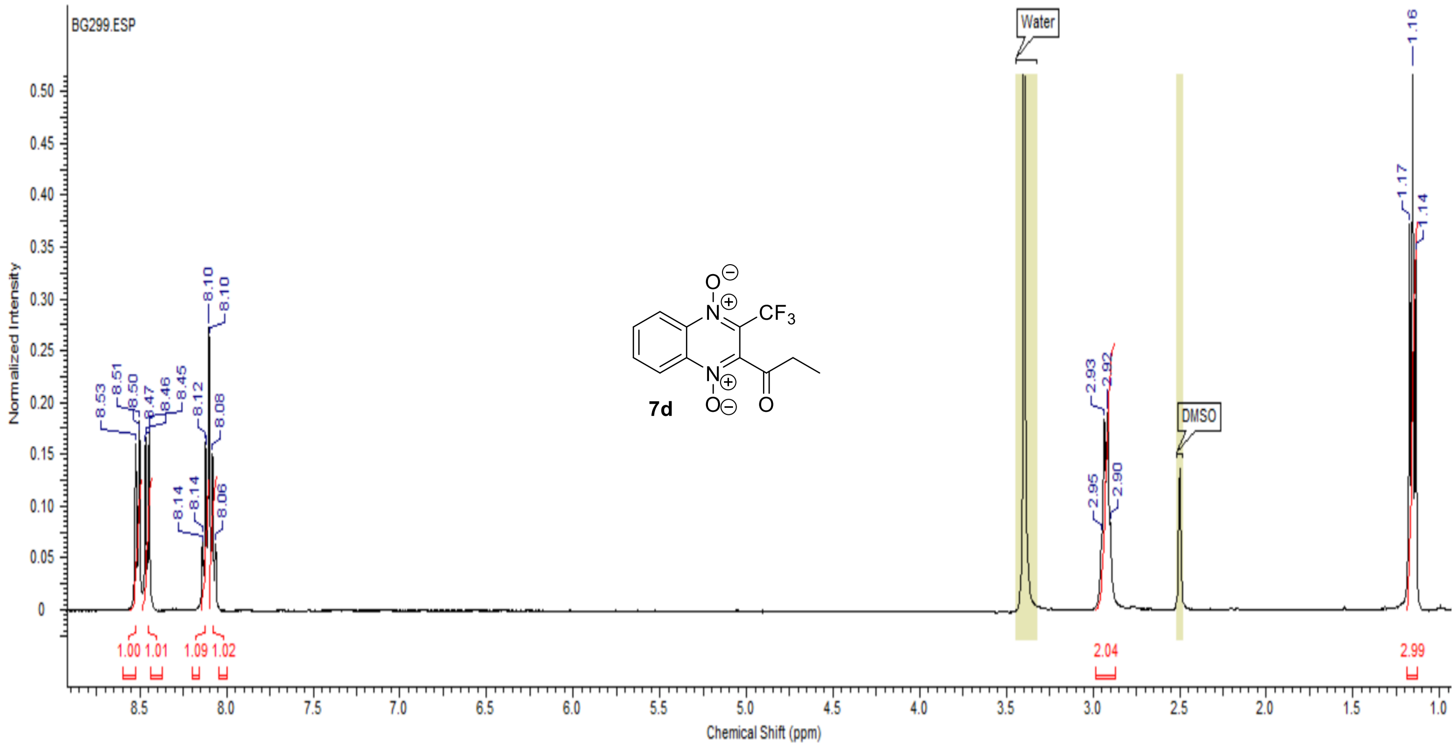

Figure S56. Copy of ^{13}C NMR spectrum of the derivative **7d**.

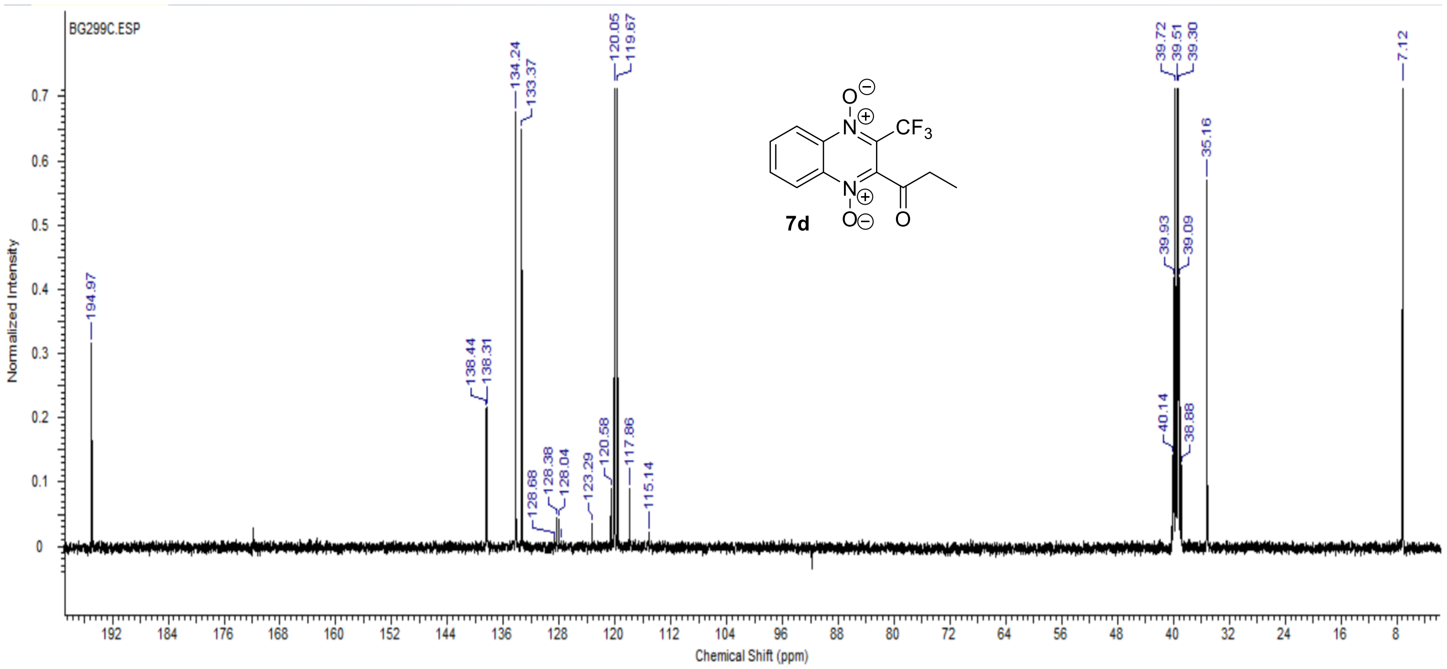

Table S1. ^{13}C chemical shifts (δ_C, ppm) and characteristic increments (I_C) for the ^{13}C chemical shift differences (relative to 2-propionyl-3-trifluoromethylquinoxaline 1,4-dioxide (**7d**)) for the piperazine group for **14a–c**.

**Position**	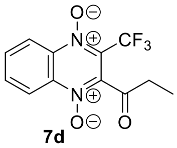 **7d**	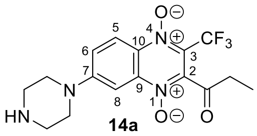 **14a**		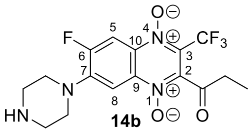 **14b**		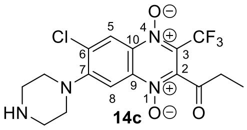 **14c**	
	**δ_C**	**δ_C**	**I^C**	**δ_C (*J*, Гц)**	**I^C**	**δ_C**	**I^C**
C-2	138.44	138.7	+0.3	136.2	−2.2	138.0	+0.3
C-3	128.2	124.4	−3.8	126.5	−1.7	127.3	−3.8
C-9	138.41	139.6	+1.2	138.4	−0.03	138.9	+1.2
C-10	138.3	131.4	−6.9	133.5 (11.5)	−4.8	134.3	−6.9

Copies of 2D NMR Spectra

Figure S57. ^1H-^{13}C HSQC spectrum for compound **13b**.

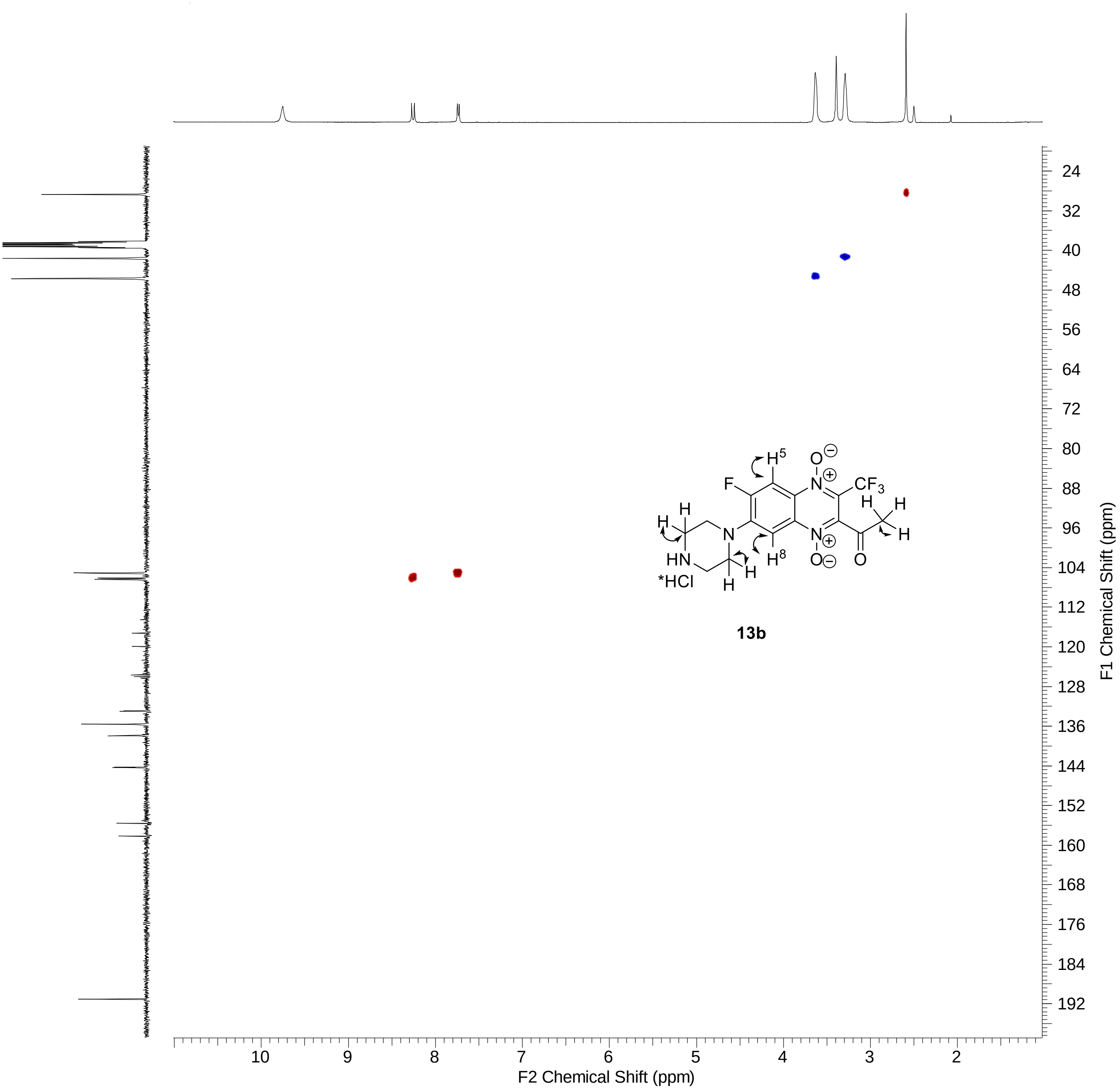

Figure S58. ^{1}H-^{13}C CIGAR-HMBC spectrum for compound **13b**.

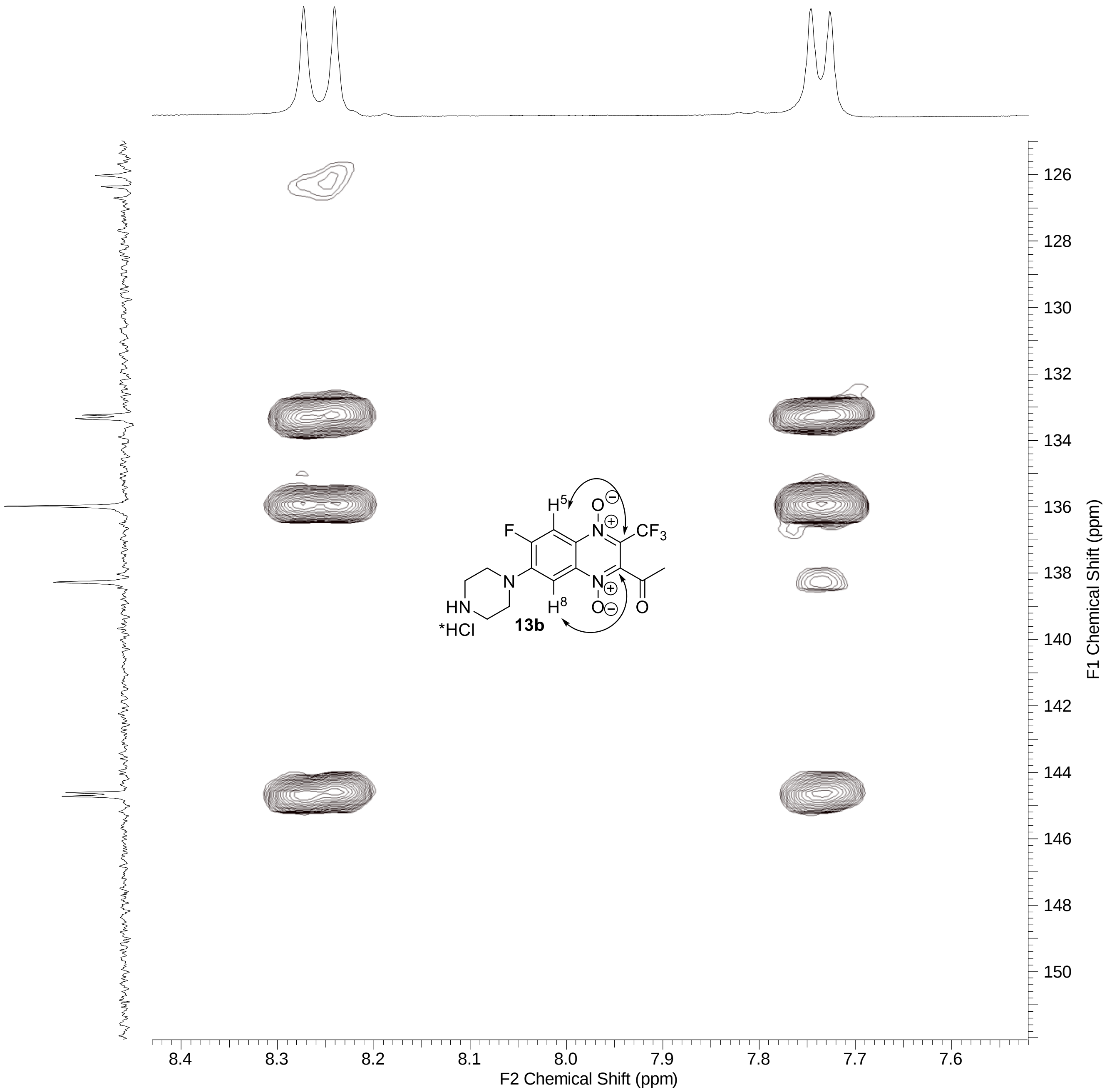

Figure S59. ^{1}H-^{13}C HSQC spectrum for compound **13c**.

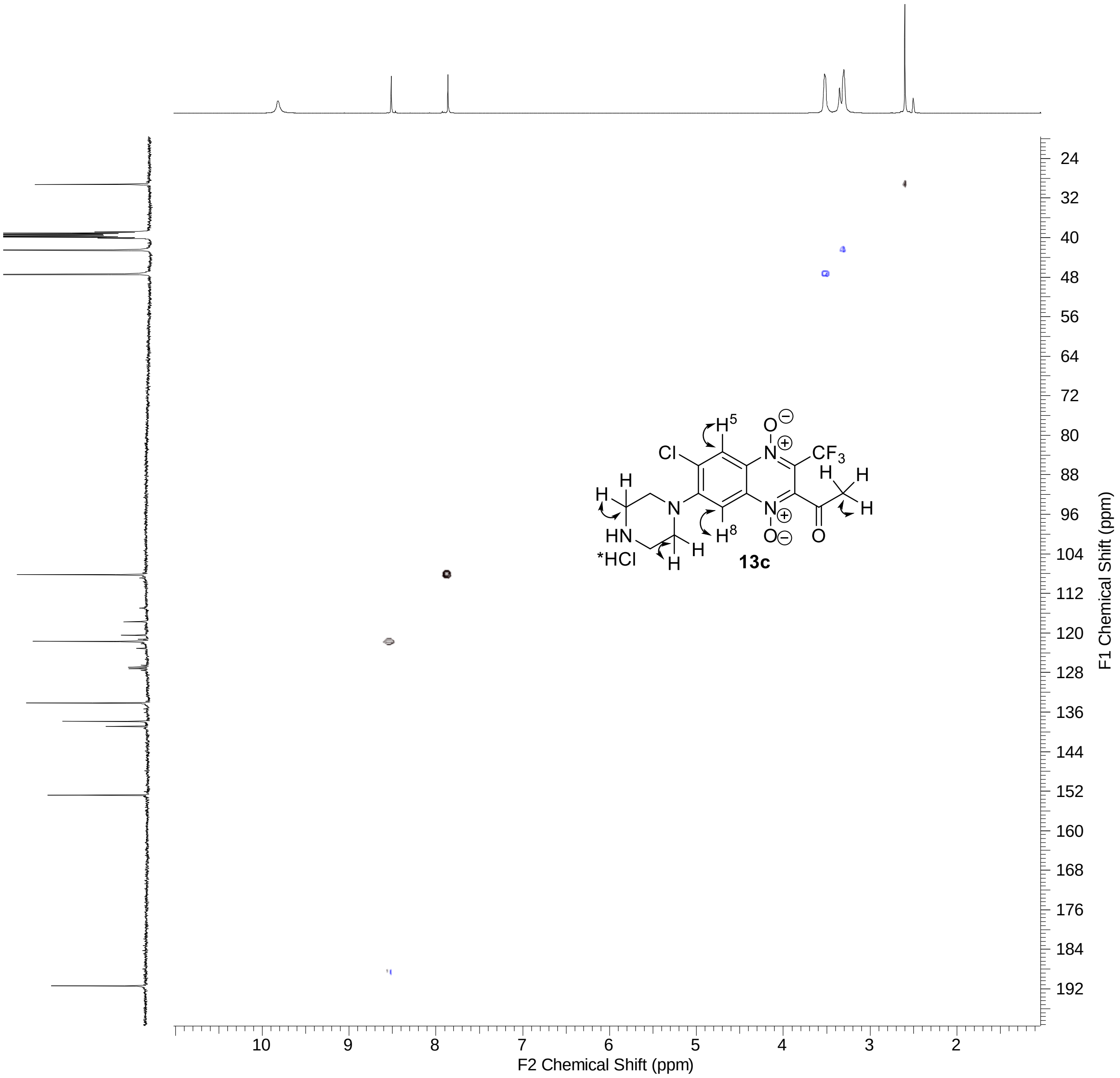

Figure S60. ^{1}H-^{13}C CIGAR-HMBC spectrum for compound **13c**.

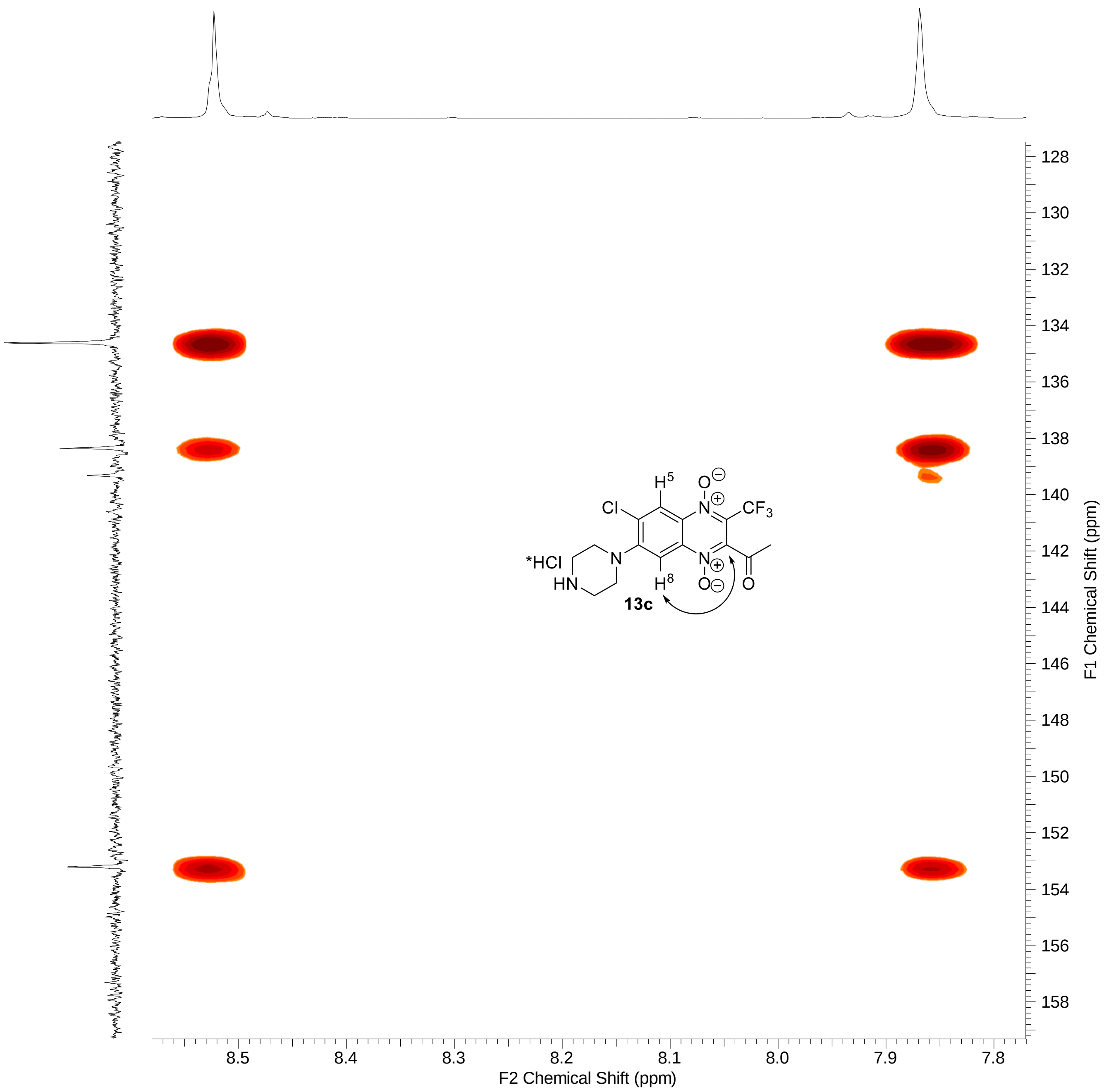

Copies of HRMS ESI Analysis

Figure S61. Copy of HRMS ESI analysis of the derivatives **6a**.

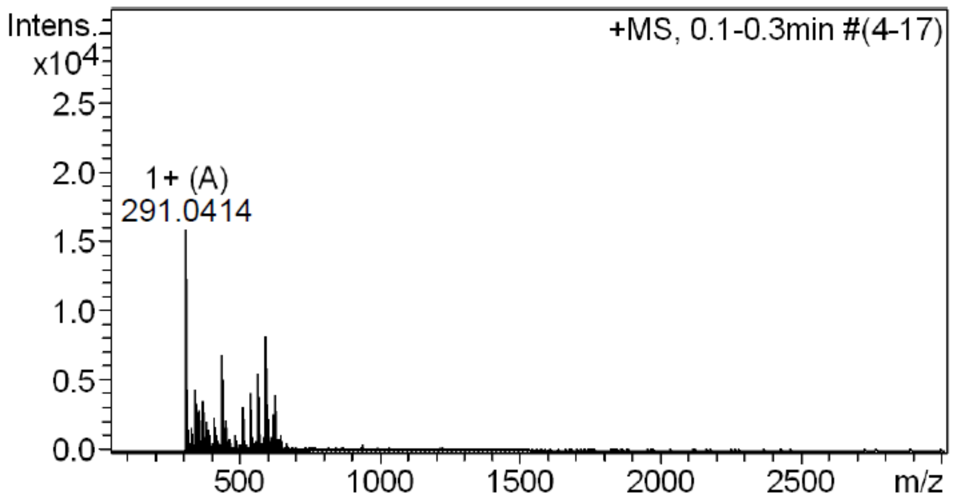

#	m/z	Res.	S/N	I	I %
1	291.0414	8243	542.6	15860	100.0
2	344.3170	7188	126.6	4345	27.4
3	372.3455	7659	89.6	3495	22.0
4	437.1918	7576	138.0	6878	43.4
5	540.5333	7924	60.9	4077	25.7
6	568.5627	9071	81.4	5428	34.2
7	596.5946	9277	123.5	8192	51.7
8	597.5950	8224	49.3	3289	20.7
9	624.6259	8501	60.5	4018	25.3

Figure S62. Copy of HRMS ESI analysis of the derivatives **7a**.

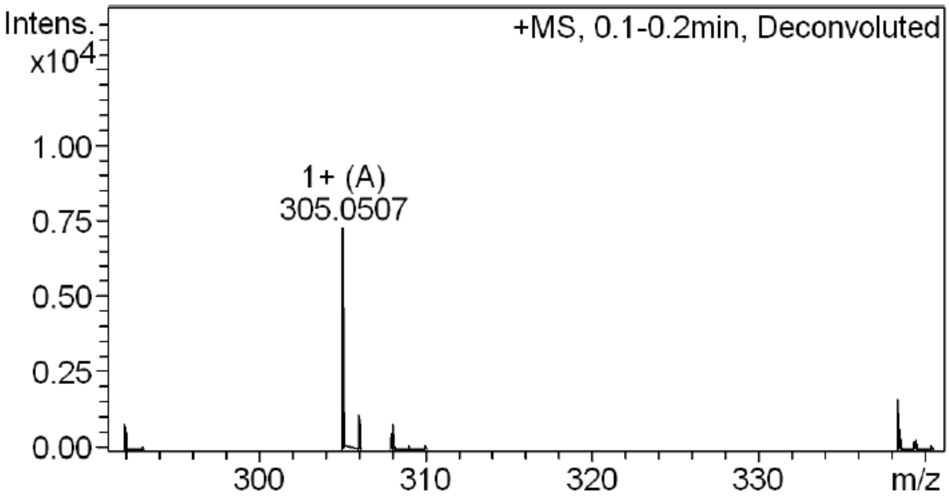

#	m/z	Res.	S/N	I	I %
1	292.0451			841	11.6
2	305.0507			7273	100.0
3	308.0185			969	13.3
4	338.3345			1727	23.7

Figure S63. Copy of HRMS ESI analysis of the derivatives **12a**.

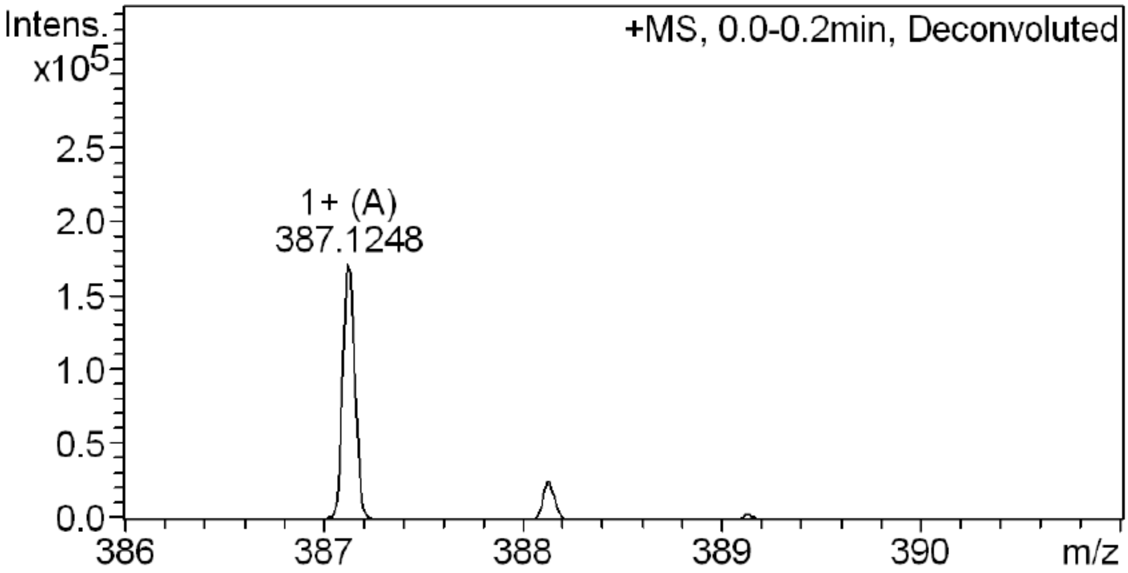

#	m/z	Res.	S/N	I	I %
1	387.1248			173053	100.0

Figure S64. Copy of HRMS ESI analysis of the derivatives **12b**.

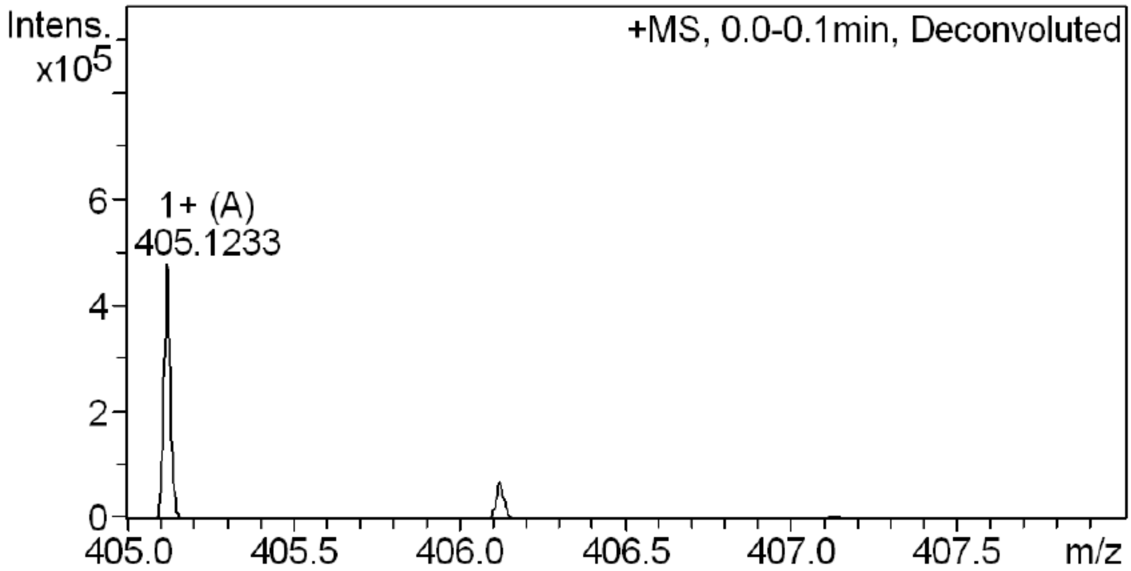

#	m/z	Res.	S/N	I	I %
1	405.1228			528259	100.0

Figure S65. Copy of HRMS ESI analysis of the derivatives **12c**.

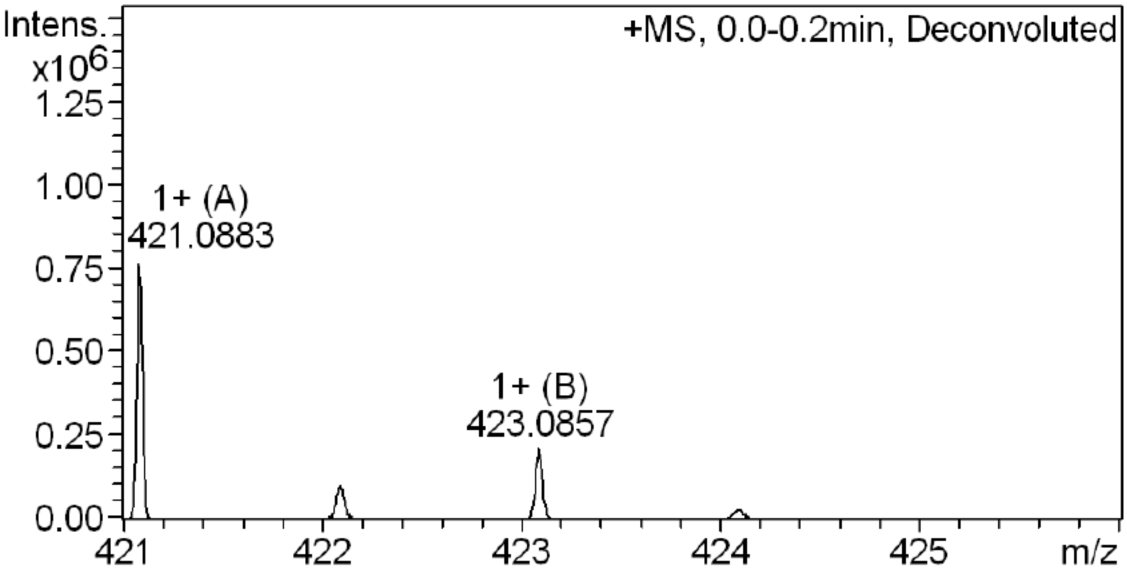

#	m/z	Res.	S/N	I	I %
1	421.0881			795749	100.0
2	423.0857			219559	27.6

Figure S66. Copy of HRMS ESI analysis of the derivatives **13a**.

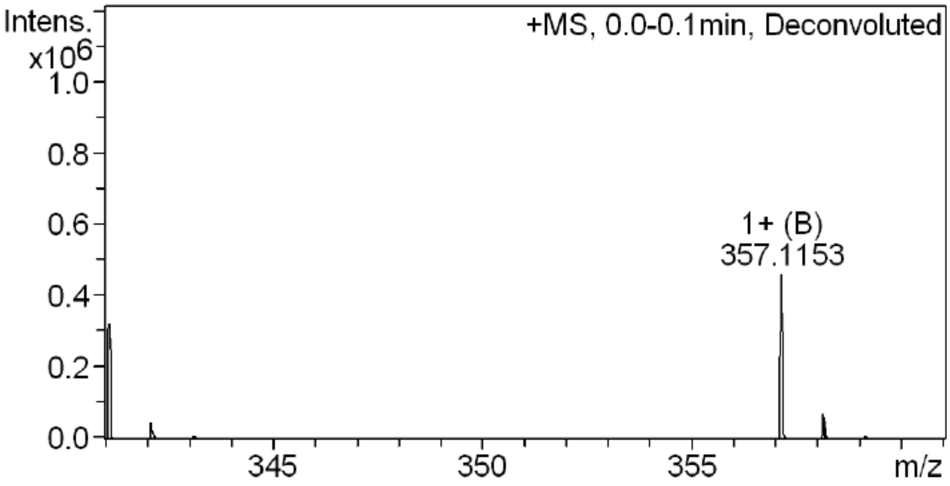

#	m/z	Res.	S/N	I	I %
1	357.1153			617939	100.0
2	341.1206			460891	74.6

Figure S67. Copy of HRMS ESI analysis of the derivatives **13b**.

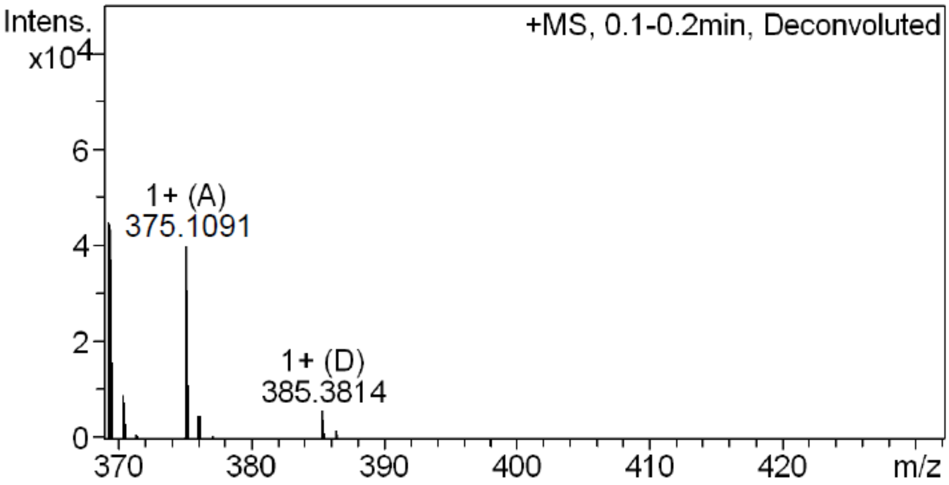

#	m/z	Res.	S/N	I	I %
1	369.3856			45475	100.0
2	375.1091			42464	93.4
3	385.3814			5987	13.2

Figure S68. Copy of HRMS ESI analysis of the derivatives **13c**.

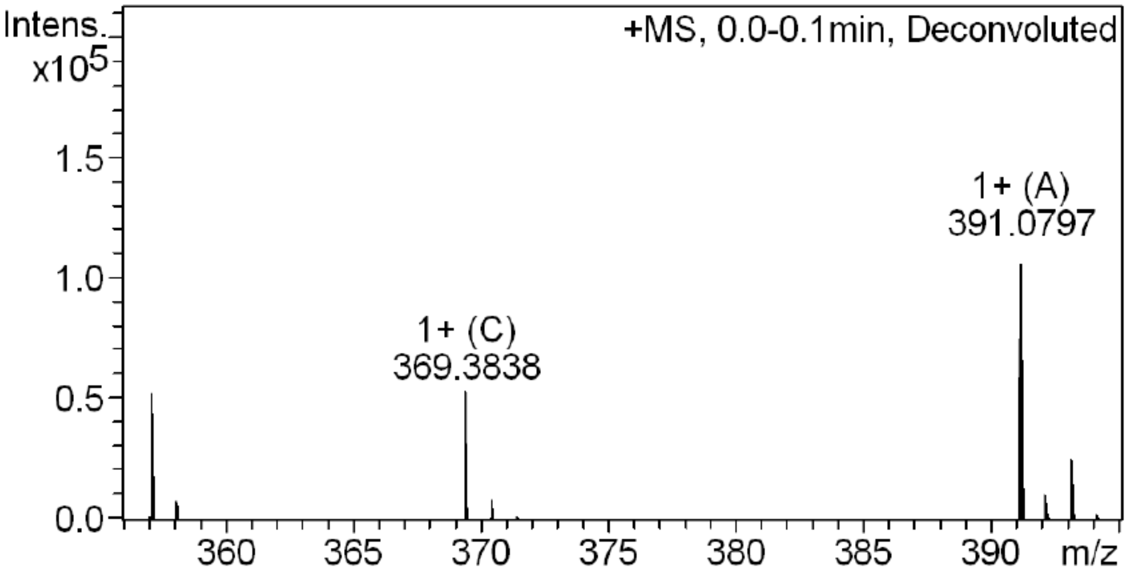

#	m/z	Res.	S/N	I	I %
1	357.1185			56018	49.3
2	369.3838			54242	47.8
3	391.0797			113521	100.0
4	393.0772			25846	22.8

Figure S69. Copy of HRMS ESI analysis of the derivatives **14a**.

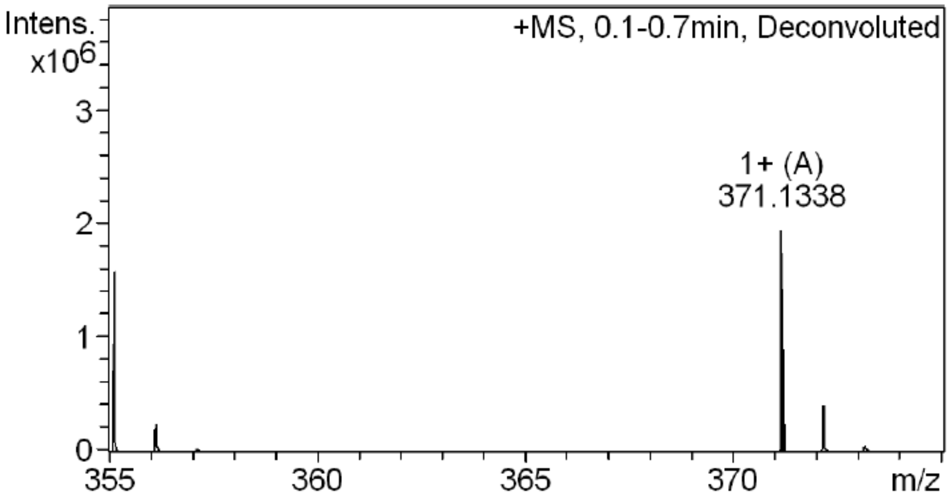

#	m/z	Res.	S/N	I	I %
1	355.1379			1584219	76.4
2	371.1338			2074941	100.0

Figure S70. Copy of HRMS ESI analysis of the derivatives **14b**.

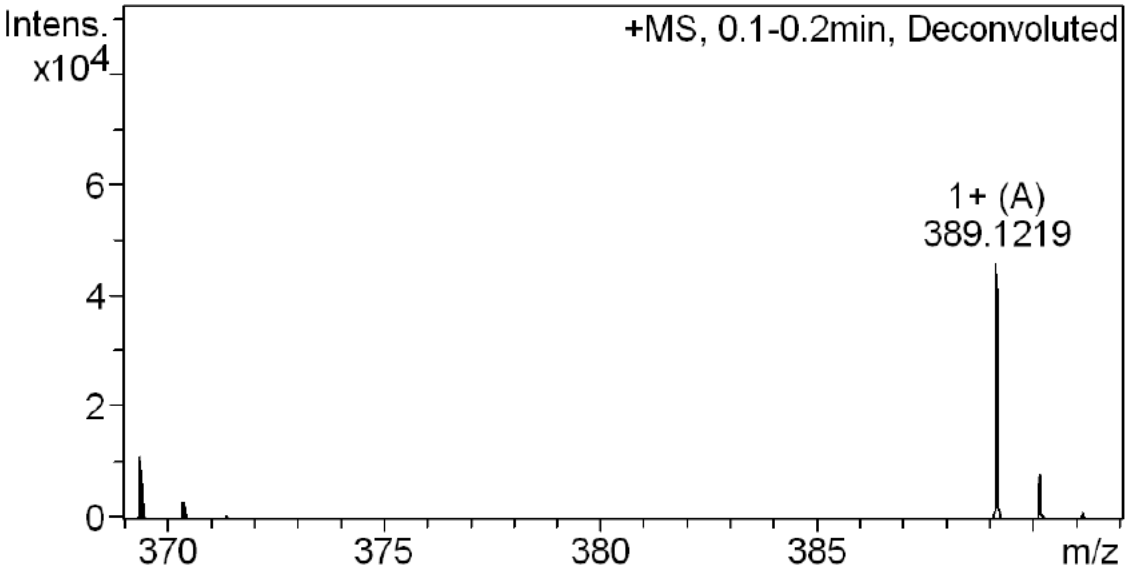

#	m/z	Res.	S/N	I	I %
1	369.3836			11475	24.3
2	389.1219			47281	100.0

Figure S71. Copy of HRMS ESI analysis of the derivatives **14c**.

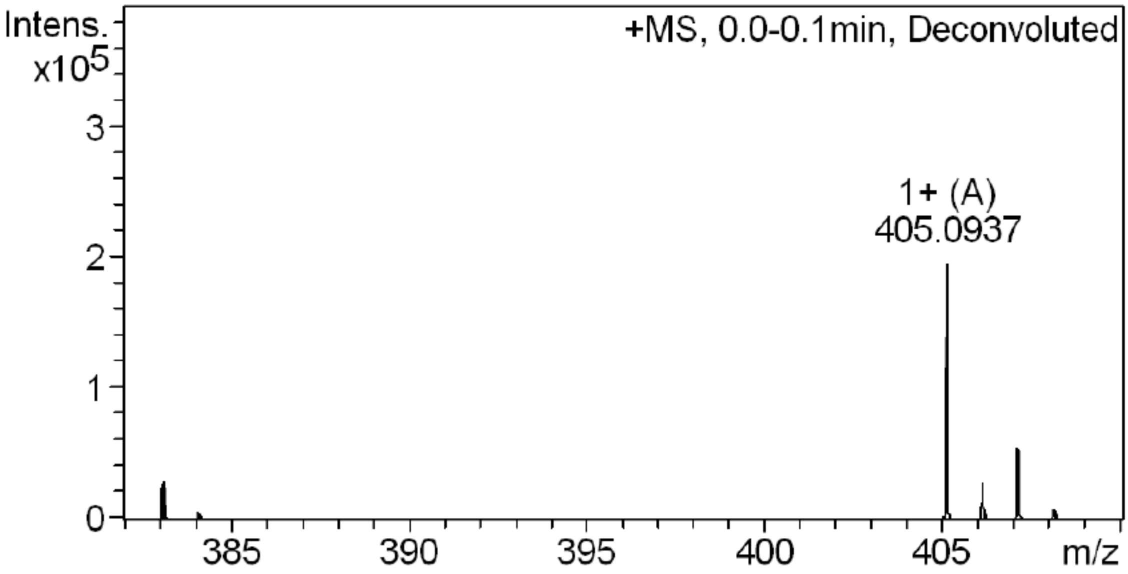

#	m/z	Res.	S/N	I	I %
1	383.1023			29871	14.6
2	405.0937			203999	100.0
3	407.0904			54865	26.9

Figure S72. Copy of HRMS ESI analysis of the derivatives **15a**.

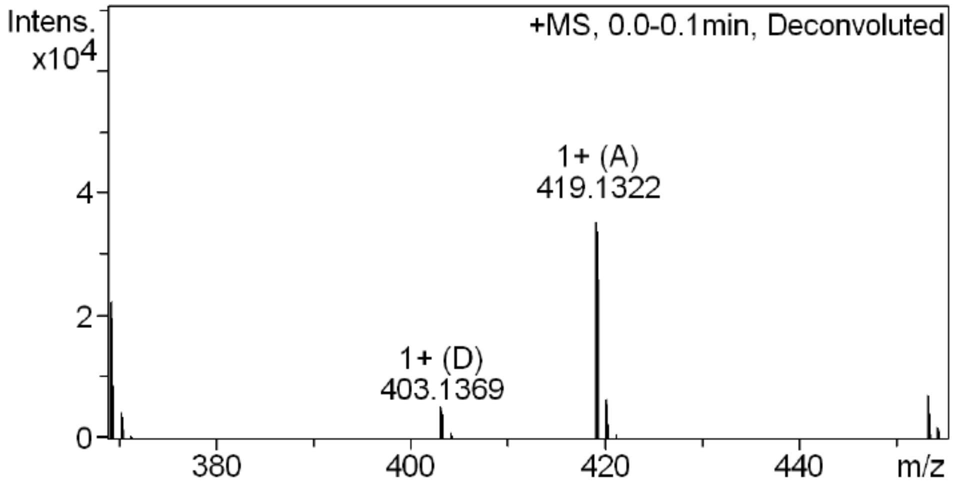

#	m/z	Res.	S/N	I	I %
1	369.3823			23177	61.3
2	403.1369			5143	13.6
3	419.1322			37838	100.0
4	453.0924			7223	19.1

Figure S73. Copy of HRMS ESI analysis of the derivatives **15b**.

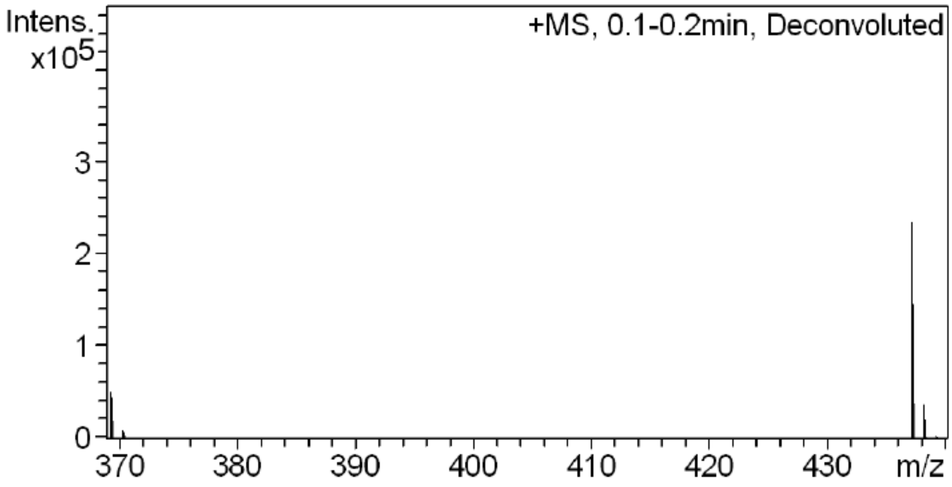

#	m/z	Res.	S/N	I	I %
1	369.3849			50780	21.3
2	437.1251			238037	100.0

Figure S74. Copy of HRMS ESI analysis of the derivatives **15c**.

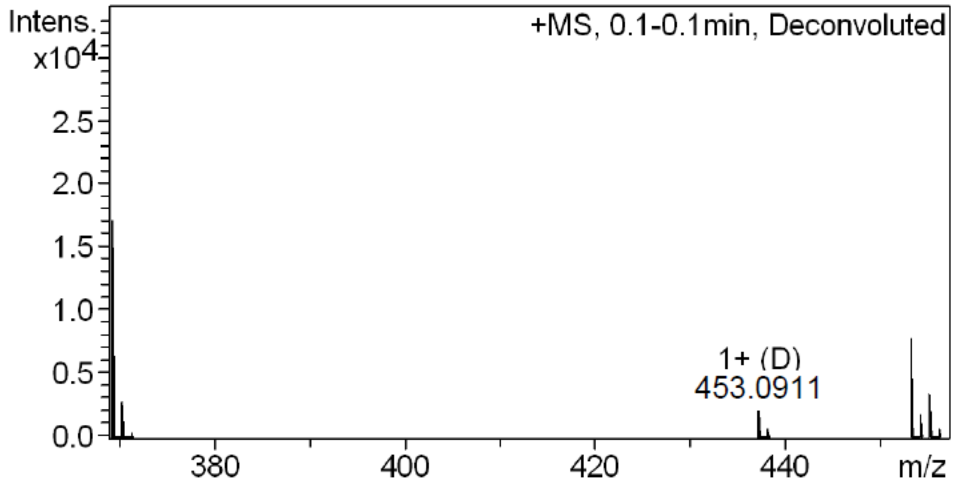

#	m/z	Res.	S/N	I	I %
1	369.3809			17802	100.0
2	437.0975			2282	12.8
3	453.0918			7963	44.7
4	455.0892			3674	20.6

Figure S75. Copy of HRMS ESI analysis of the derivatives **15d**.

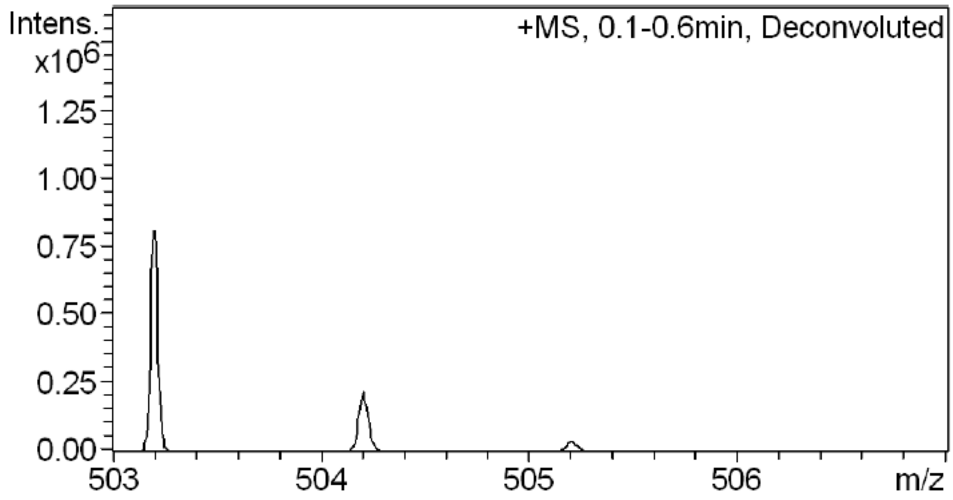

#	m/z	Res.	S/N	I	I %
1	503.2001			810008	100.0

Figure S76. Copy of HRMS ESI analysis of the derivatives **16a**.

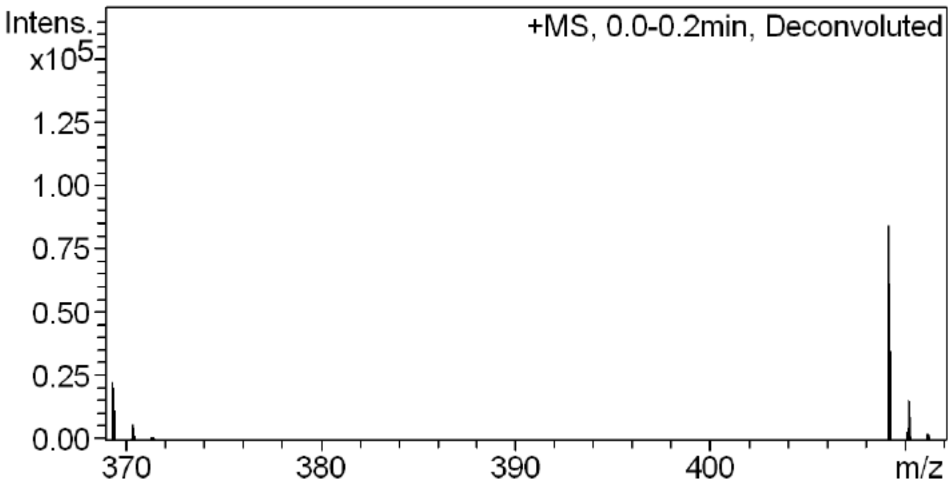

#	m/z	Res.	S/N	I	I %
1	369.3793			22877	25.4
2	409.1073			89917	100.0

Figure S77. Copy of HRMS ESI analysis of the derivatives **16b**.

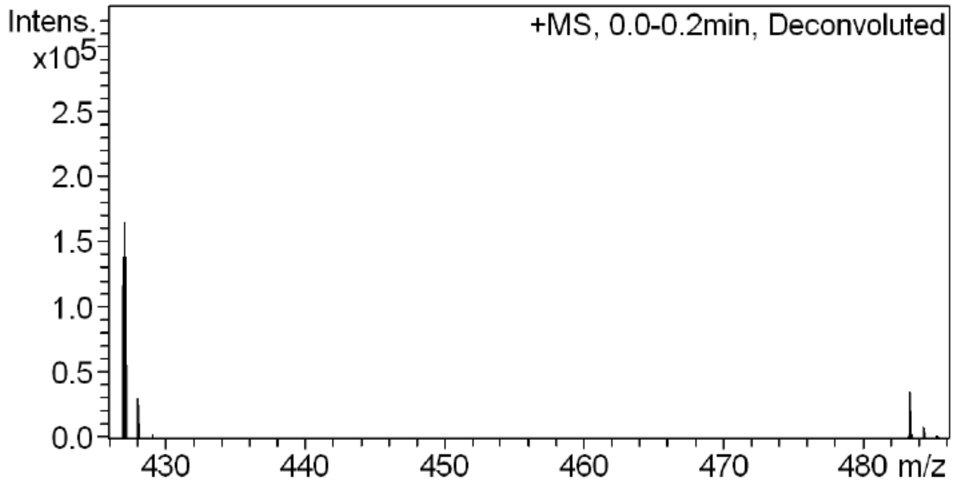

#	m/z	Res.	S/N	I	I %
1	427.1028			166372	100.0
2	483.2318			35718	21.5

Figure S78. Copy of HRMS ESI analysis of the derivatives **16c**.

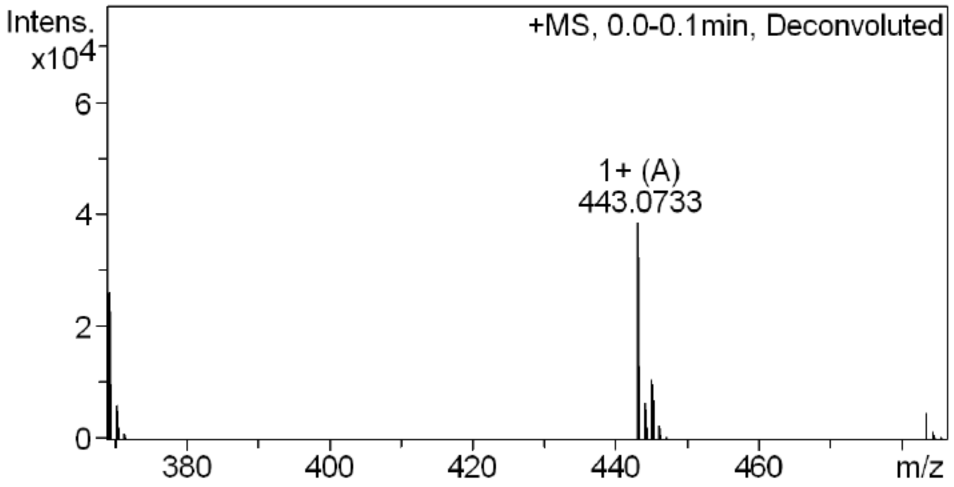

#	m/z	Res.	S/N	I	I %
1	369.3839			27579	71.1
2	443.0733			38793	100.0
3	445.0718			10844	28.0
4	483.2339			4797	12.4

Figure S79. Copy of HRMS ESI analysis of the derivatives **16d**.

+MS, 0.0-0.1min #(2-8)

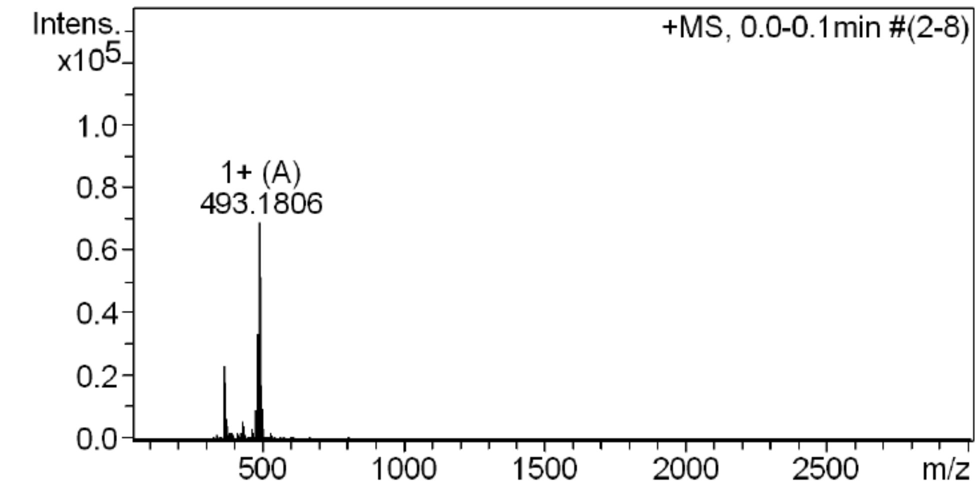

#	m/z	Res.	S/N	I	I %
1	369.3840			23224	33.8
2	477.1856			9119	13.3
3	483.2308			33499	48.7
4	493.1803			68762	100.0

Figure S80. Copy of HRMS ESI analysis of the derivatives **17a**.

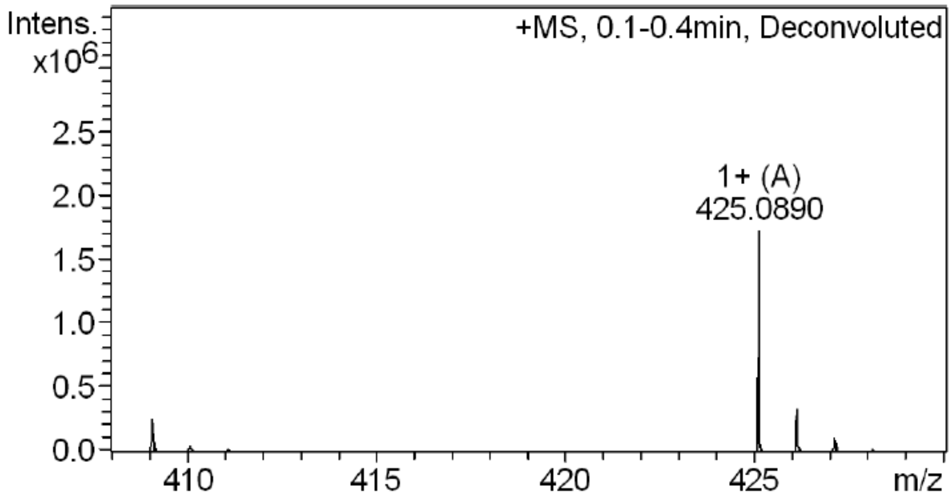

#	m/z	Res.	S/N	I	I %
1	409.0925			265624	15.2
2	425.0890			1749713	100.0

Figure S81. Copy of HRMS ESI analysis of the derivatives **17b**.

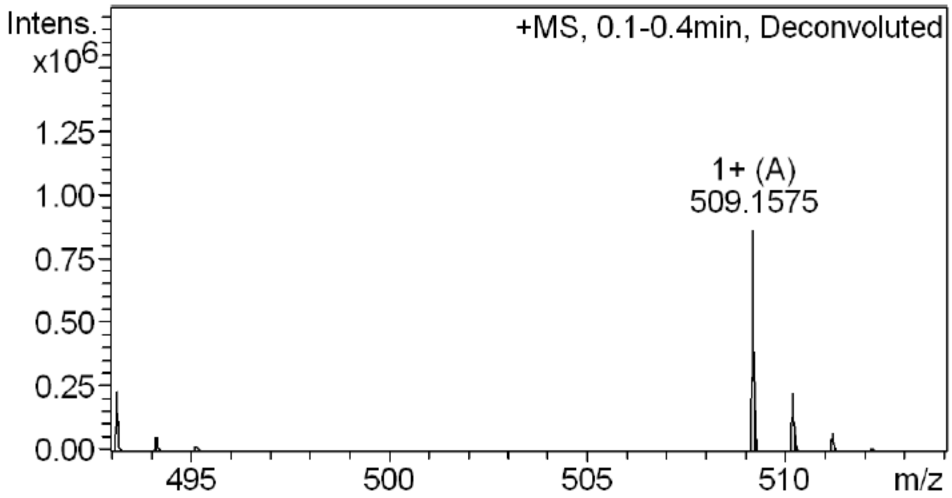

#	m/z	Res.	S/N	I	I %
1	493.1608			235929	26.3
2	509.1575			898682	100.0

Figure S82. Copy of HRMS ESI analysis of the derivatives **17c**.

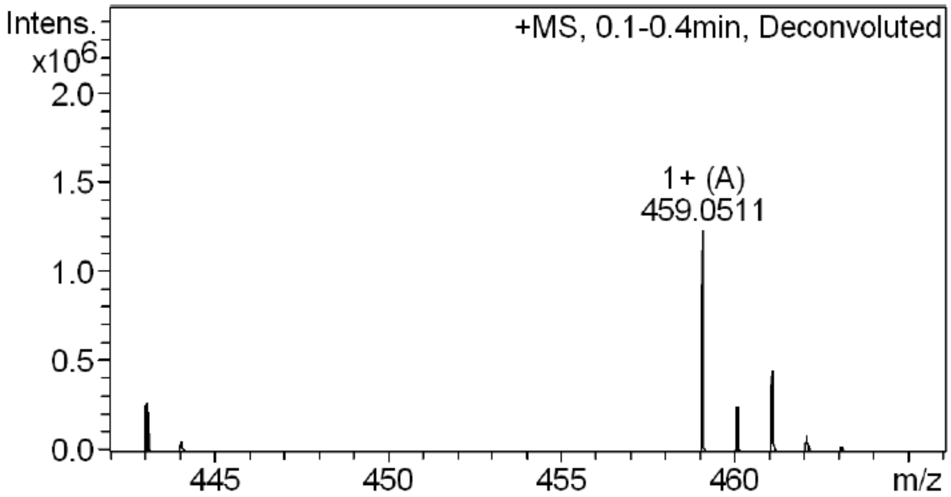

#	m/z	Res.	S/N	I	I %
1	443.0547			285054	23.1
2	459.0511			1235932	100.0
3	461.0467			474006	38.4

Figure S83. Copy of HRMS ESI analysis of the derivatives **17d**.

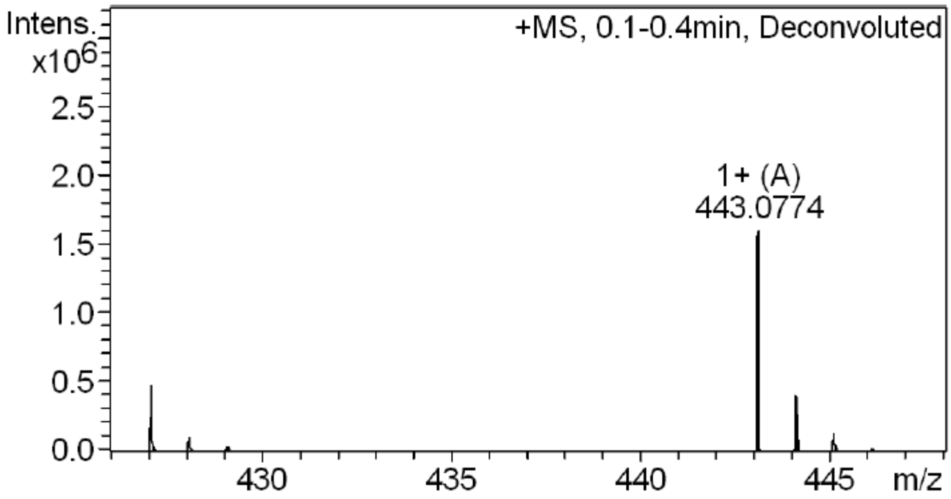

#	m/z	Res.	S/N	I	I %
1	427.0815			505638	31.0
2	443.0774			1630288	100.0

Figure S84. Copy of HRMS ESI analysis of the derivatives **18a**.

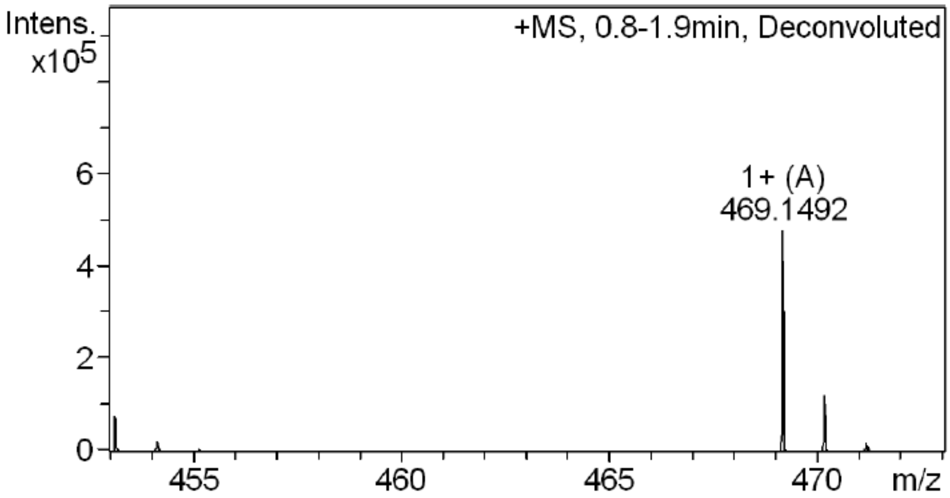

#	m/z	Res.	S/N	I	I %
1	453.1517			81992	17.0
2	469.1492			481758	100.0

Figure S85. Copy of HRMS ESI analysis of the derivatives **18b**.

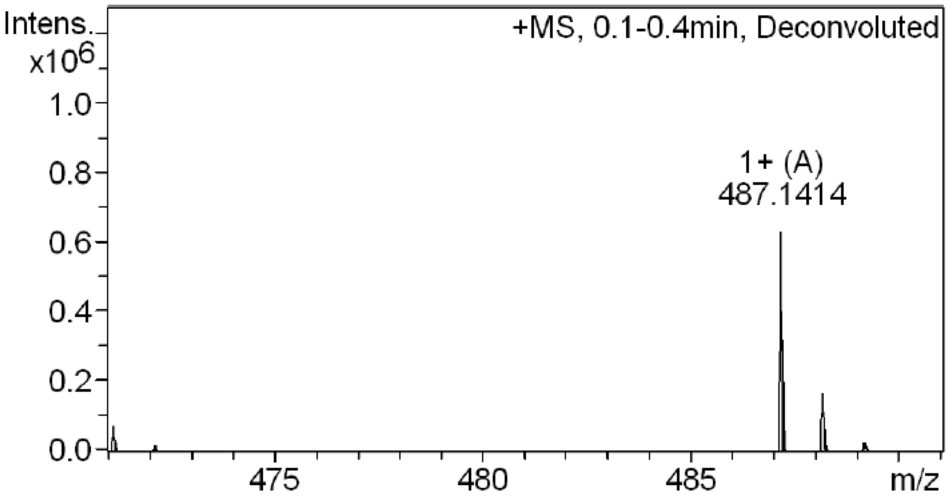

#	m/z	Res.	S/N	I	I %
1	471.1455			74406	10.9
2	487.1414			683657	100.0

Figure S86. Copy of HRMS ESI analysis of the derivatives **18c**.

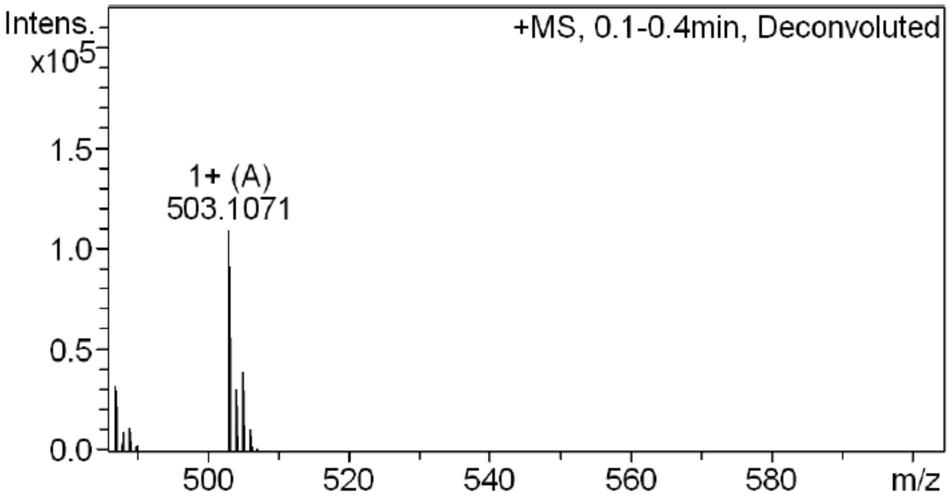

#	m/z	Res.	S/N	I	I %
1	487.1108			33433	30.2
2	503.1071			110735	100.0
3	505.1058			39076	35.3

Figure S87. Copy of HRMS ESI analysis of the derivatives **18d**.

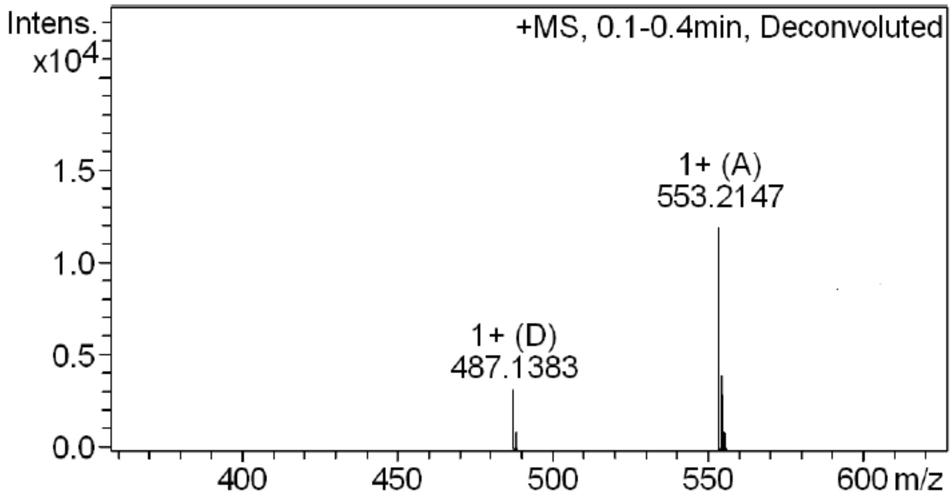

#	m/z	Res.	S/N	I	I %
1	487.1383			3352	26.7
2	553.2147			12541	100.0

Copies of HPLC Analysis

Figure S88. Copy HPLC analysis of the derivative **12a**.

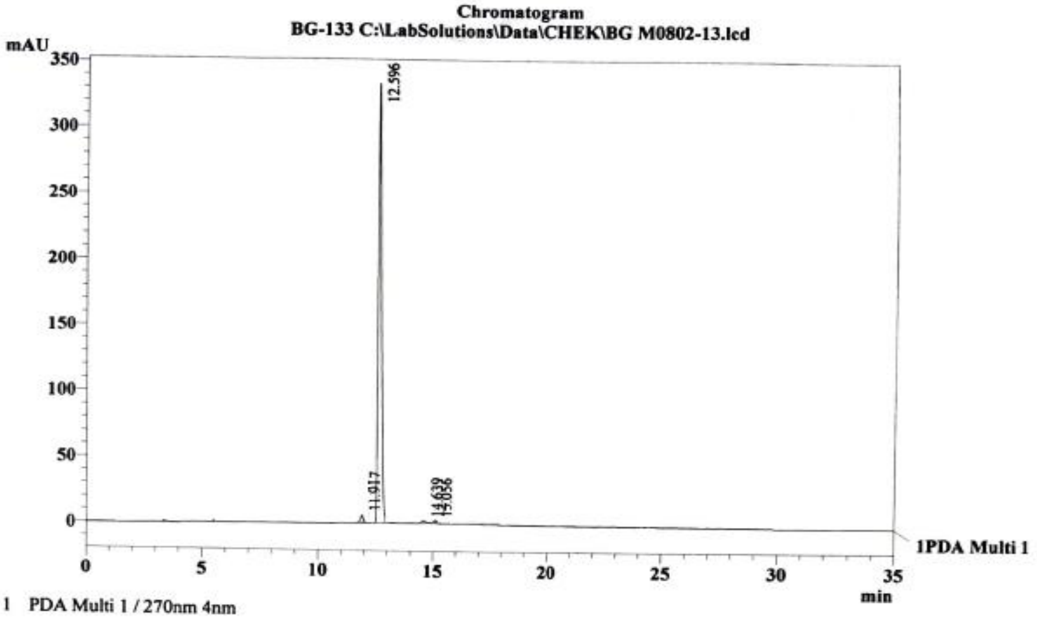

1 PDA Multi 1 / 270nm 4nm

PeakTable

PDA Ch1 270nm 4nm

Peak#	Ret. Time	Area	Height	Area %
1	11.917	35314	5197	0.983
2	12.596	3531604	332109	98.258
3	14.639	16625	1606	0.463
4	15.056	10678	1623	0.297
Total		3594221	340534	100.000

Method

<<LC Program>>

Time	Unit	Command	Value
0.10	Pumps	B.Conc	20
30.00	Pumps	B.Conc	70
33.00	Pumps	B.Conc	20
43.00	Controller	Stop	

Method Filename : FOS Bv.lcm

Shimadzu LC-20AD; 2-System FOS, Colon Kromasil 100-C18,.size 5mkm, 4,6*250mm, N 86912
Elution: A - H3PO4 0.01M pH 2.6; B - MeCN, fl. 1,0 ml/min, loop 20mkl.

Figure S89. Copy HPLC analysis of the derivative **12b**.

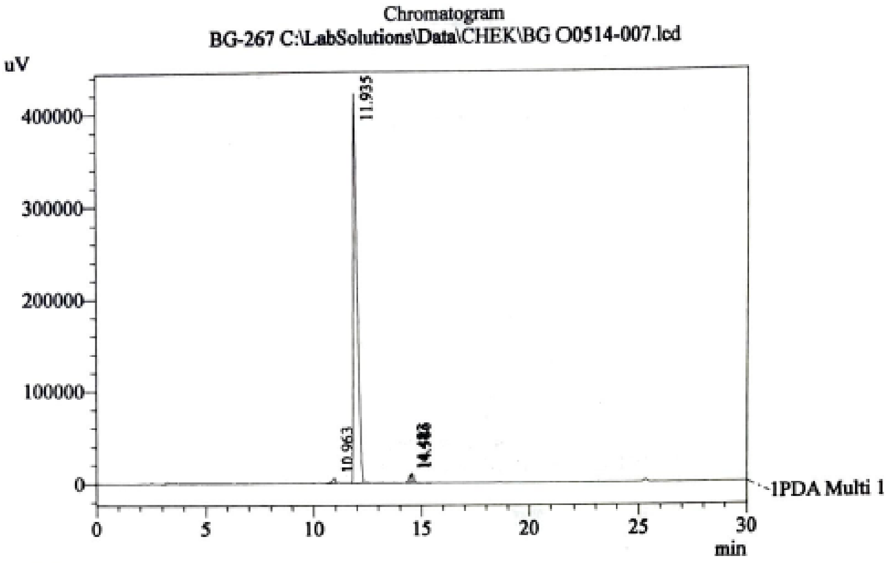

1 PDA Multi 1 / 335nm 4nm

PDA Ch1 335nm 4nm

Peak#	Ret. Time	Area	Height	Area %
1	10.963	59020	5953	1.023
2	11.935	5570934	419270	96.538
3	14.483	58454	8588	1.013
4	14.586	82319	10042	1.426
Total		5770728	443853	100.000

Method Filename : FOS Cv.lcm 15.05.2019 12:02:57

Time	Unit	Command	Valu
0.10	Pumps	B.Conc	20
30.00	Pumps	B.Conc	80
33.00	Pumps	B.Conc	20
45.00	Controller	Stop	

Shimadzu LC-20 AD; System - FOS Colon- Kromasil-100-5mkm. C-18, 4,6x250 mm. N 62511
Elution: A - H3PO4 0,01M pH 2,6; B - MeCN, fl - 1.0 ml/min, loop 20 mkl

Figure S90. Copy HPLC analysis of the derivative **12c**.

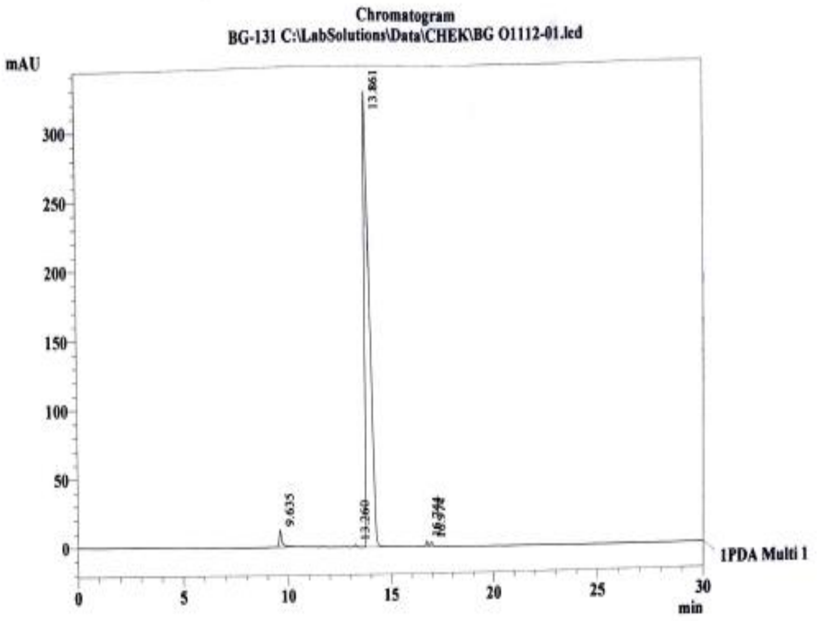

1 PDA Multi 1 / 280nm 4nm

PeakTable

PDA Ch1 280nm 4nm

Peak#	Ret. Time	Area	Height	Area %
1	9.635	115727	12578	2.261
2	13.260	9718	1720	0.190
3	13.861	4947465	326451	96.640
4	16.744	26363	4086	0.515
5	16.974	20182	2983	0.394
Total		5119455	347818	100.000

Method

<<LC Program>>

Time	Unit	Command	Value
0.10	Pumps	B.Conc	20
30.00	Pumps	B.Conc	80
33.00	Pumps	B.Conc	20
45.00	Controller	Stop	

Method Filename : FOS Bv.lcm

Shimadzu LC-20AD; 2-System FOS, Colon Kromasil 100-C18,.size 5mkm, 4,6*250mm, N 86912
Elution: A - H3PO4 0.01M pH 2.6; B - MeCN, fl. 1,0 ml/min, loop 20mkl.

Figure S91. Copy HPLC analysis of the derivative **13a**.

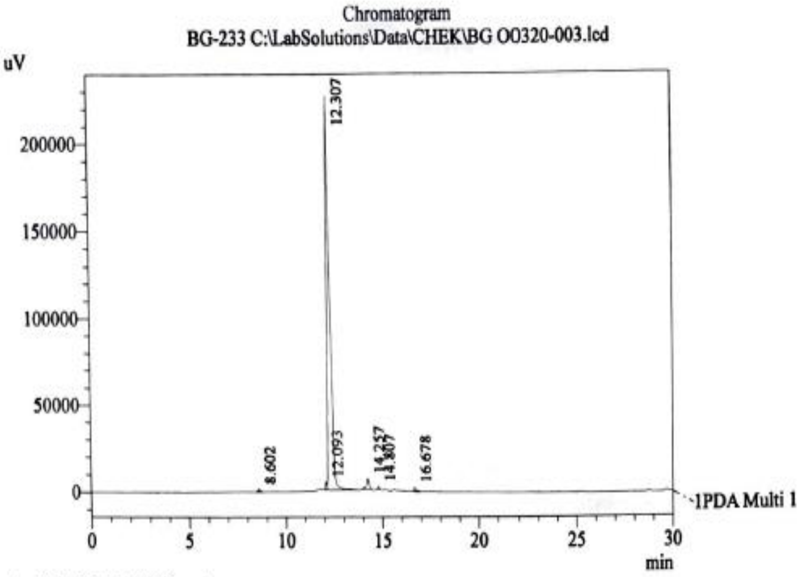

1 PDA Multi 1 / 336nm 4nm

PDA Ch1 336nm 4nm

Peak#	Ret. Time	Area	Height	Area %
1	8.602	12828	1522	0.473
2	12.093	28383	4661	1.046
3	12.307	2577588	225758	95.026
4	14.257	66260	6593	2.443
5	14.807	9100	1706	0.335
6	16.678	18348	2448	0.676
Total		2712507	242689	100.000

Method Filename : FOS B.lcm 22.03.2019 12:06:12

Time	Unit	Command	Valu
0.01	Pumps	B.Conc	10
30.00	Pumps	B.Conc	90
33.00	Pumps	B.Conc	10
45.00	Controller	Stop	

Shimadzu LC-20 AD; System - FOS Colon- Kromasil-100-5mkm. C-18, 4,6x250 mm. N 62511
Elution: A - H3PO4 0,01M pH 2,6; B - MeCN, fl - 1.0 ml/min, loop 20 mkl

Figure S92. Copy HPLC analysis of the derivative **13b**.

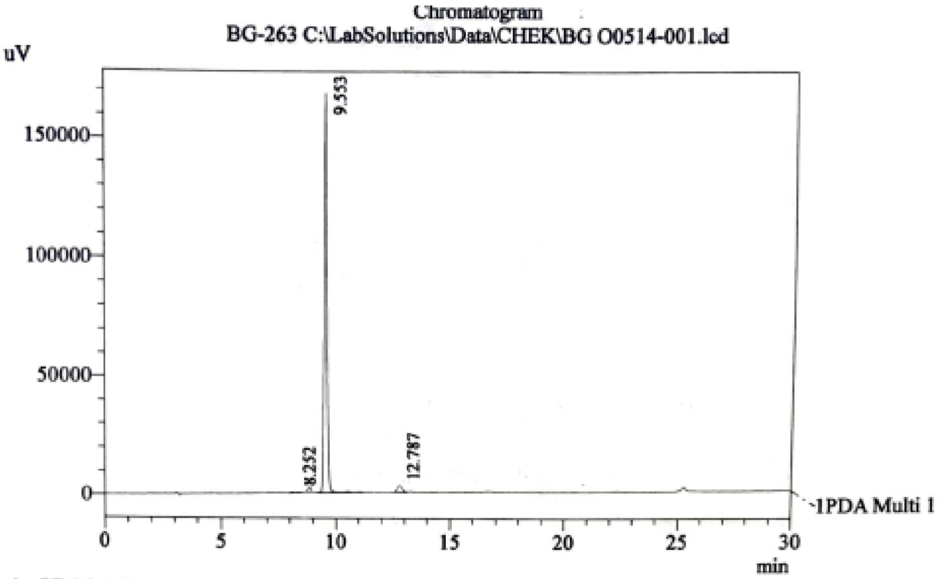

1 PDA Multi 1 / 335nm 4nm

PDA Ch1 335nm 4nm

Peak#	Ret. Time	Area	Height	Area %
1	8.252	16229	1052	1.056
2	9.553	1476035	167291	96.034
3	12.787	44726	3030	2.910
Total		1536990	171372	100.000

Method Filename : FOS Cv.lcm 14.05.2019 11:35:44

Time	Unit	Command	Valu
0.10	Pumps	B.Conc	20
30.00	Pumps	B.Conc	80
33.00	Pumps	B.Conc	20
45.00	Controller	Stop	

Shimadzu LC-20 AD; System - FOS Colon- Kromasil-100-5mkm. C-18, 4,6x250 mm. N 62511
Elution: A - H3PO4 0,01M pH 2,6; B - MeCN, fl - 1.0 ml/min, loop 20 mkl

Figure S93. Copy HPLC analysis of the derivative **13c**.

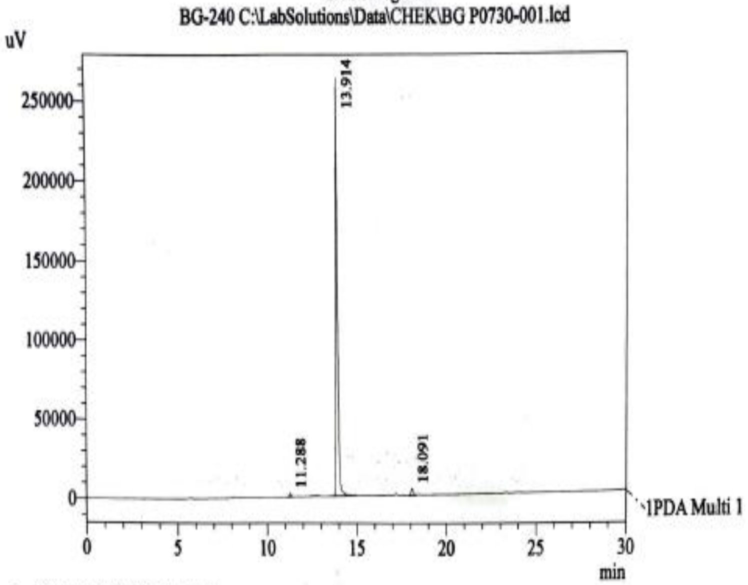

1 PDA Multi 1 / 282nm 4nm

PDA Ch1 282nm 4nm

Peak#	Ret. Time	Area	Height	Area %
1	11.288	16610	2356	0.791
2	13.914	2043760	261903	97.370
3	18.091	38589	4247	1.838
Total		2098959	268505	100.000

Method Filename : FRA02Bv.lcm 30.07.2020 11:47:43

Time	Unit	Command	Valu
0.01	Pumps	B.Conc	20
30.00	Pumps	B.Conc	60
33.00	Pumps	B.Conc	20
45.00	Controller	Stop	

Shimadzu LC-20 AD; System - FOS Colon- Kromasil-100-5mkm. C-18, 4,6x250 mm. N 62511
Elution: A - H3PO4 0,01M pH 2,6; B - MeCN, fl - 1.0 ml/min, loop 20 mkl

Figure S94. Copy HPLC analysis of the derivative **14a**.

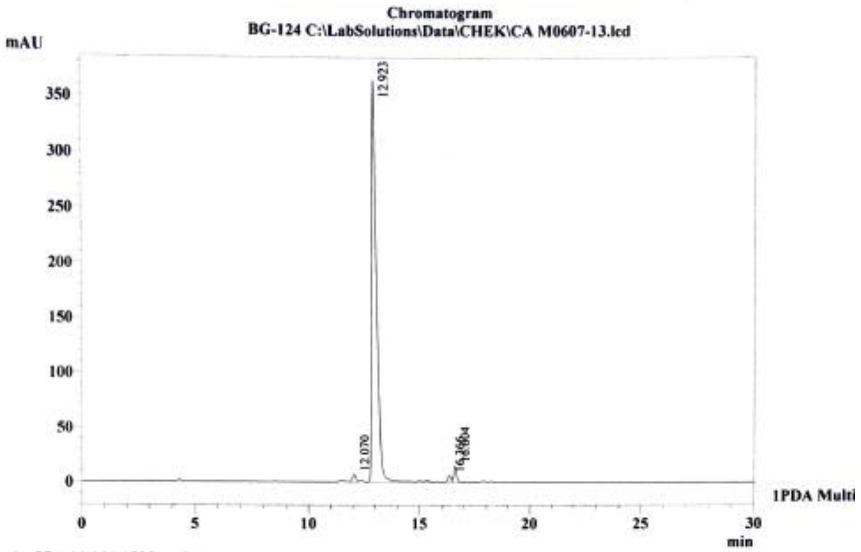

1 PDA Multi 1 / 335nm 4nm

PeakTable

PDA Ch1 335nm 4nm

Peak#	Ret. Time	Area	Height	Area %
1	12.070	71650	7000	1.246
2	12.923	5496103	365027	95.593
3	16.366	53490	6481	0.930
4	16.604	128246	15340	2.231
Total		5749489	393848	100.000

Method

<<LC Program>>

Time	Unit	Command	Value
0.10	Pumps	B.Conc	20
30.00	Pumps	B.Conc	60
33.00	Pumps	B.Conc	20
43.00	Controller	Stop	

Method Filename : FOS Bv.lcm

Shimadzu LC-20AD; 2-System FOS, Colon Kromasil 100-C18,.size 5mkm, 4,6*250mm, N 86912
Elution: A - H3PO4 0.01M pH 2.6; B - MeCN, fl. 1,0 ml/min, loop 20mkl.

Figure S95. Copy HPLC analysis of the derivative **14b**.

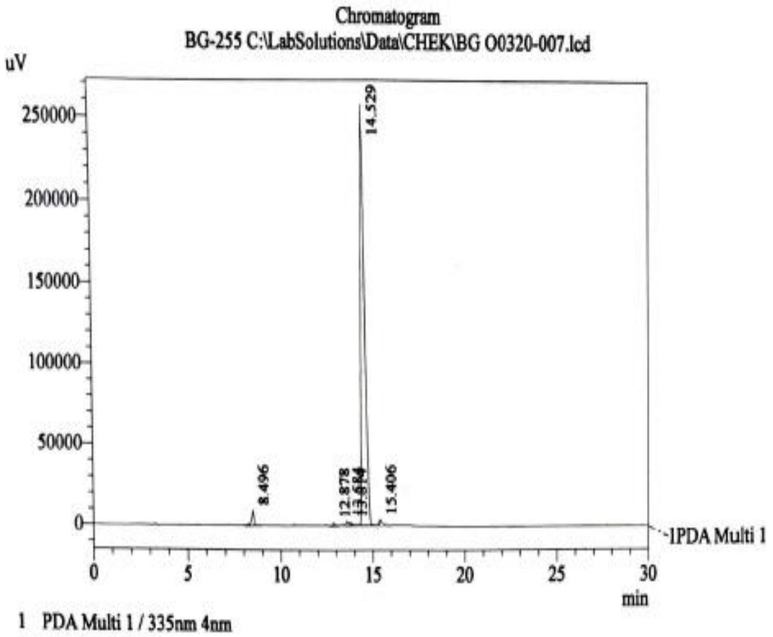

1 PDA Multi 1 / 335nm 4nm

PDA Ch1 335nm 4nm

Peak#	Ret. Time	Area	Height	Area %
1	8.496	90003	9615	2.308
2	12.878	16135	2004	0.414
3	13.584	33972	2892	0.871
4	13.814	14572	1811	0.374
5	14.529	3719342	256981	95.380
6	15.406	25470	3212	0.653
Total		3899494	276516	100.000

Method Filename : FOS B.lcm 22.03.2019 15:32:07

Time	Unit	Command	Valu
0.01	Pumps	B.Conc	10
30.00	Pumps	B.Conc	90
33.00	Pumps	B.Conc	10
45.00	Controller	Stop	

Shimadzu LC-20 AD; System - FOS Colon- Kromasil-100-5mkm. C-18, 4,6x250 mm. N 62511
Elution: A - H3PO4 0,01M pH 2,6; B - MeCN, fl - 1.0 ml/min, loop 20 mkl

Figure S96. Copy HPLC analysis of the derivative **14c**.

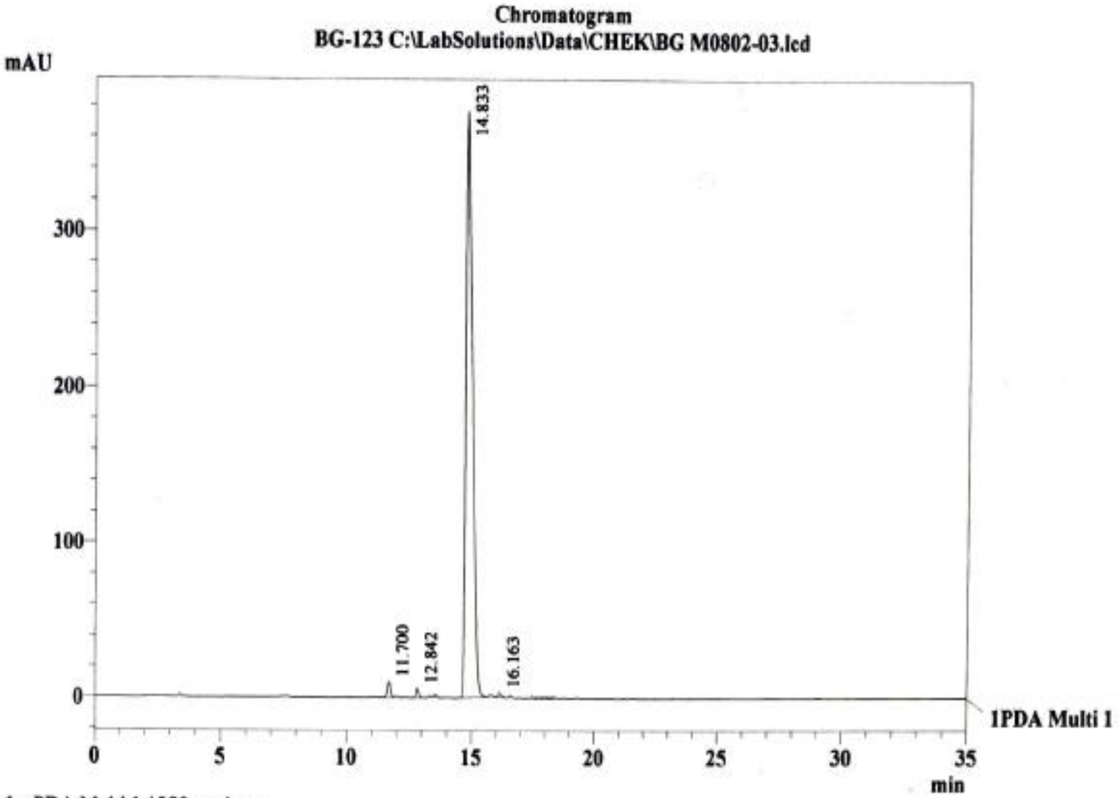

1 PDA Multi 1 / 280nm 4nm

PeakTable

PDA Ch1 280nm 4nm

Peak#	Ret. Time	Area	Height	Area %
1	11.700	84428	10293	1.217
2	12.842	44681	6264	0.644
3	14.833	6782069	375700	97.787
4	16.163	24403	3024	0.352
Total		6935581	395280	100.000

Method

<<LC Program>>

Time	Unit	Command	Value
0.10	Pumps	B.Conc	20
30.00	Pumps	B.Conc	70
33.00	Pumps	B.Conc	20
43.00	Controller	Stop	

Method Filename : FOS Bv.lcm

Shimadzu LC-20AD; 2-System FOS, Colon Kromasil 100-C18,.size 5mkm, 4,6*250mm, N 86912
Elution: A - H3PO4 0.01M pH 2.6; B - MeCN, fl. 1,0 ml/min, loop 20mkl.

Figure S97. Copy HPLC analysis of the derivative **15a**.

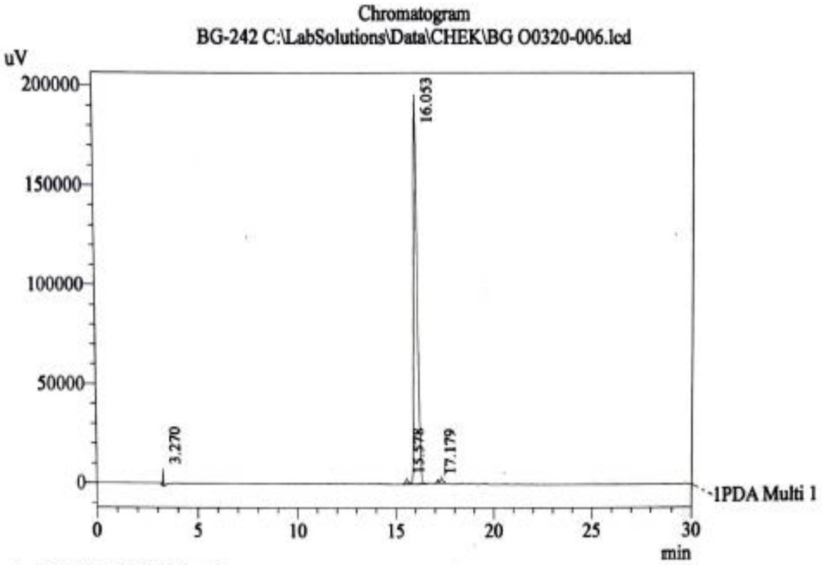

1 PDA Multi 1 / 340nm 4nm

PDA Ch1 340nm 4nm

Peak#	Ret. Time	Area	Height	Area %
1	3.270	21969	8485	0.853
2	15.578	26467	2958	1.027
3	16.053	2516775	195299	97.695
4	17.179	10948	1669	0.425
Total		2576159	208412	100.000

Method Filename : FOS B.lcm 22.03.2019 14:46:27

Time	Unit	Command	Valu
0.01	Pumps	B.Conc	10
30.00	Pumps	B.Conc	90
33.00	Pumps	B.Conc	10
45.00	Controller	Stop	

Shimadzu LC-20 AD; System - FOS Colon- Kromasil-100-5mkm. C-18, 4,6x250 mm. N 62511
Elution: A - H3PO4 0,01M pH 2,6; B - MeCN, fl - 1.0 ml/min, loop 20 mkl

Figure S98. Copy HPLC analysis of the derivative **15b**.

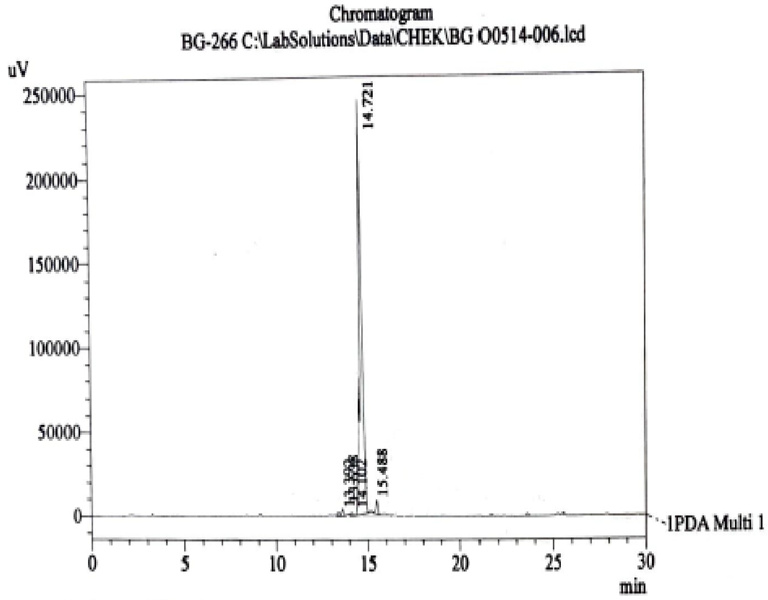

1 PDA Multi 1 / 270nm 4nm

PDA Ch1 270nm 4nm

Peak#	Ret. Time	Area	Height	Area %
1	13.392	20917	2455	0.688
2	13.638	39466	4817	1.299
3	14.102	16347	1907	0.538
4	14.721	2892005	242954	95.164
5	15.488	70246	8382	2.311
Total		3038981	260516	100.000

Method Filename : FOS Cv.lcm 15.05.2019 11:03:01

Time	Unit	Command	Valu
0.10	Pumps	B.Conc	20
30.00	Pumps	B.Conc	80
33.00	Pumps	B.Conc	20
45.00	Controller	Stop	

Shimadzu LC-20 AD; System - FOS Colon- Kromasil-100-5mkm. C-18, 4,6x250 mm. N 62511
Elution: A - H3PO4 0,01M pH 2,6; B - MeCN, fl - 1.0 ml/min, loop 20 mkl

Figure S99. Copy HPLC analysis of the derivative **15c**.

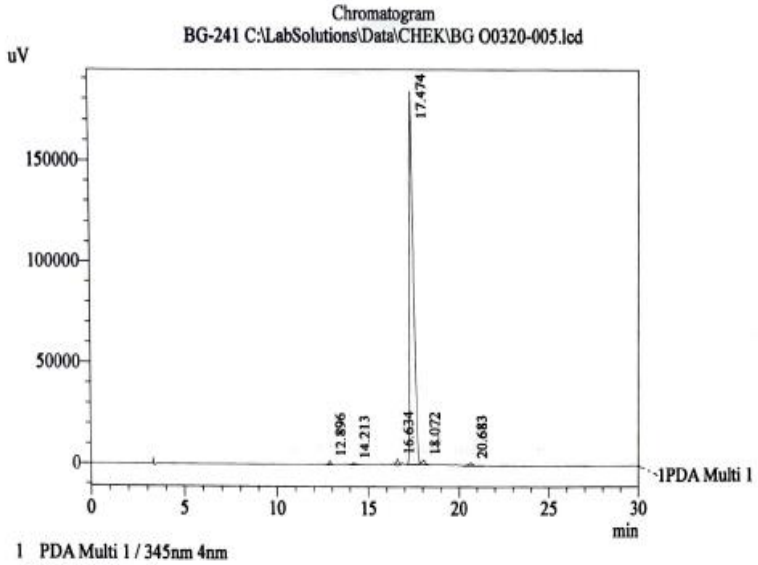

1 PDA Multi 1 / 345nm 4nm

PDA Ch1 345nm 4nm

Peak#	Ret. Time	Area	Height	Area %
1	12.896	12848	1759	0.434
2	14.213	9028	796	0.305
3	16.634	26626	3032	0.899
4	17.474	2864072	183756	96.726
5	18.072	29139	2378	0.984
6	20.683	19310	1454	0.652
Total		2961023	193175	100.000

Method Filename : FOS B.lcm 22.03.2019 13:50:10

Time	Unit	Command	Valu
0.01	Pumps	B.Conc	10
30.00	Pumps	B.Conc	90
33.00	Pumps	B.Conc	10
45.00	Controller	Stop	

Shimadzu LC-20 AD; System - FOS Colon- Kromasil-100-5mkm. C-18, 4,6x250 mm. N 62511
Elution: A - H3PO4 0,01M pH 2,6; B - MeCN, fl - 1.0 ml/min, loop 20 mkl

Figure S100. Copy HPLC analysis of the derivative **15d**.

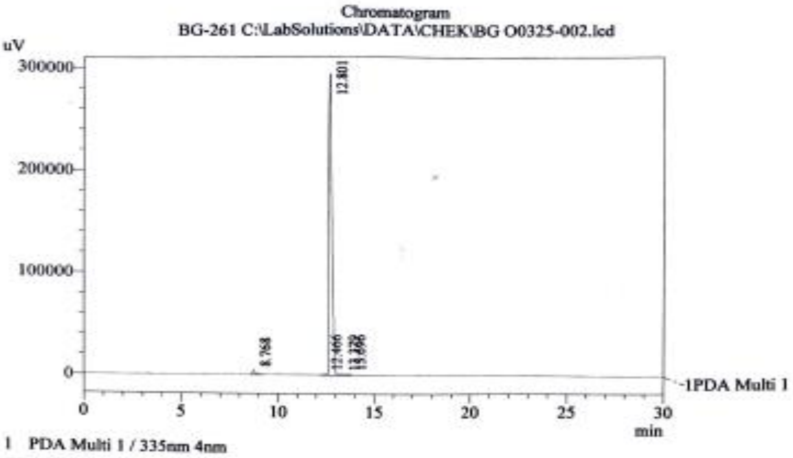

1 PDA Multi 1 / 335nm 4nm

PDA Ch1 335nm 4nm

Peak#	Ret. Time	Area	Height	Area %
1	8.768	37814	4130	1.077
2	12.466	11948	1262	0.340
3	12.801	3436193	293355	97.853
4	13.379	10935	863	0.311
5	13.696	14703	1413	0.419
Total		3511593	301024	100.000

Method Filename : FOS B.lcm 25.03.2019 12:44:40

Time	Unit	Command	Valı
0.01	Pumps	B.Conc	10
30.00	Pumps	B.Conc	90
33.00	Pumps	B.Conc	10
45.00	Controller	Stop	

Shimadzu LC-20 AD; System - FOS Colon- Kromasil-100-5mkm. C-18, 4,6x250 mm. N 62511
Elution: A - H3PO4 0,01M pH 2,6; B - MeCN, fl - 1.0 ml/min, loop 20 mkl

Figure S101. Copy HPLC analysis of the derivative **16a**.

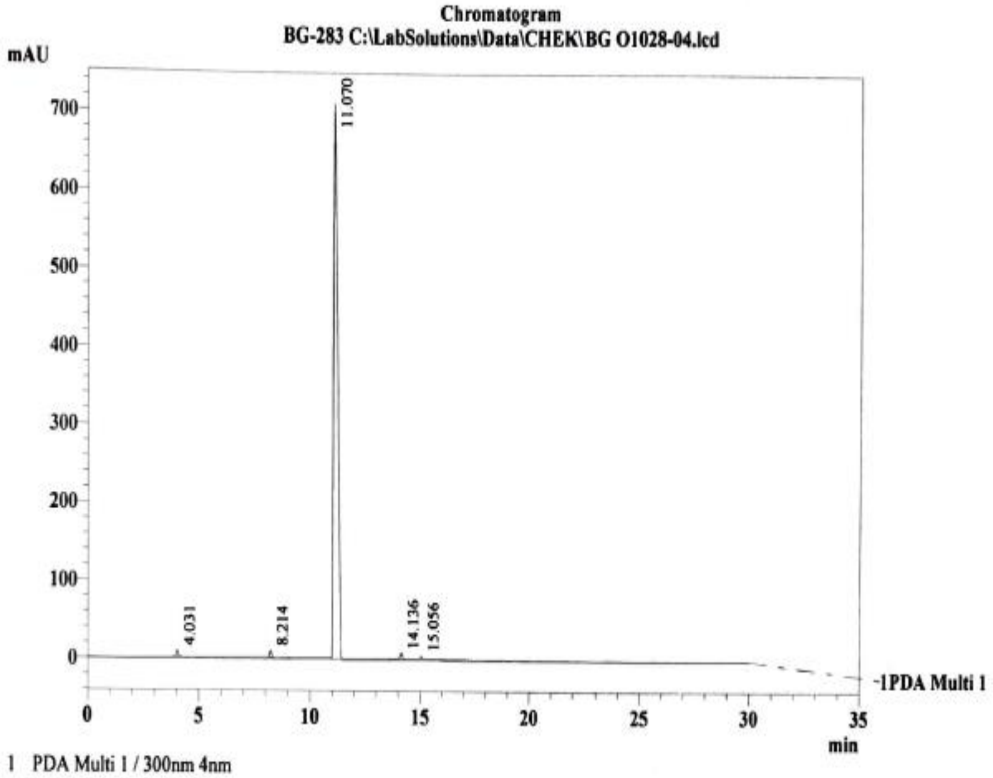

1 PDA Multi 1 / 300nm 4nm

PeakTable

PDA Ch1 300nm 4nm

Peak#	Ret. Time	Area	Height	Area %
1	4.031	54755	9470	0.594
2	8.214	62143	10153	0.675
3	11.070	9015662	710338	97.872
4	14.136	55219	7987	0.599
5	15.056	23880	4778	0.259
Total		9211660	742726	100.000

Method

<<LC Program>>

Time	Unit	Command	Value
0.10	Pumps	B.Conc	20
30.00	Pumps	B.Conc	80
33.00	Pumps	B.Conc	20
45.00	Controller	Stop	

Method Filename : FOS Bv.lcm

Shimadzu LC-20AD; 2-System FOS, Colon Kromasil 100-C18,.size 5mkm, 4,6*250mm, N 86912
Elution: A - H3PO4 0.01M pH 2.6; B - MeCN, fl. 1,0 ml/min, loop 20mkl.

Figure S102. Copy HPLC analysis of the derivative **16b**.

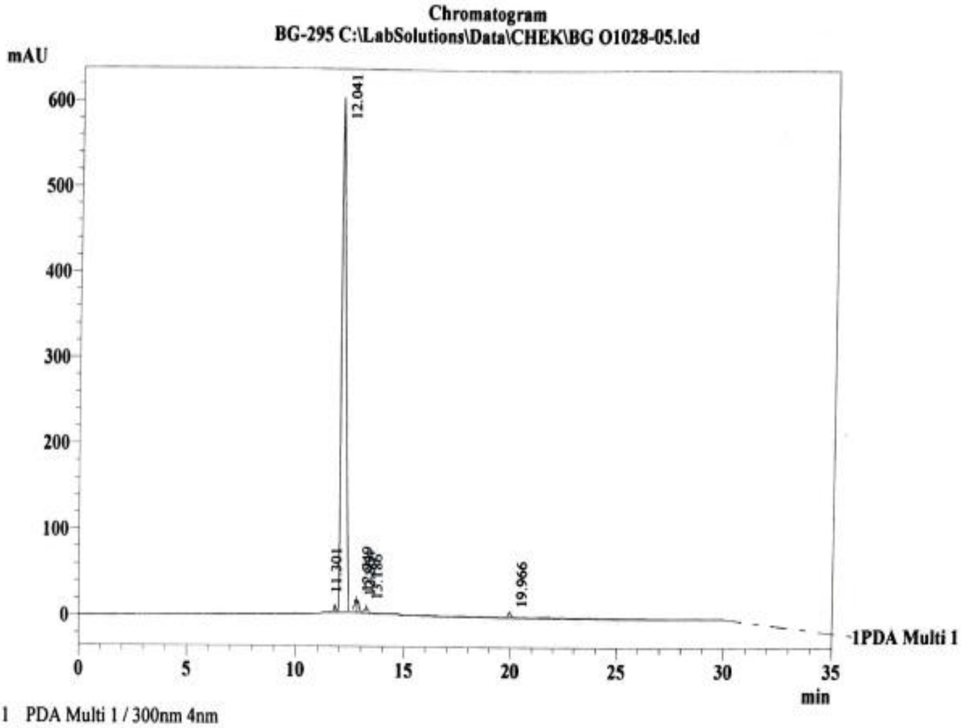

1 PDA Multi 1 / 300nm 4nm

PeakTable

PDA Ch1 300nm 4nm

Peak#	Ret. Time	Area	Height	Area %
1	11.301	70426	15169	0.798
2	12.041	8430647	598641	95.525
3	12.749	139503	19092	1.581
4	12.864	85081	14156	0.964
5	13.186	38471	7836	0.436
6	19.966	61480	7220	0.697
Total		8825608	662114	100.000

Method

<<LC Program>>

Time	Unit	Command	Value
0.10	Pumps	B.Conc	20
30.00	Pumps	B.Conc	80
33.00	Pumps	B.Conc	20
45.00	Controller	Stop	

Method Filename : FOS Bv.lcm

Shimadzu LC-20AD; 2-System FOS, Colon Kromasil 100-C18,.size 5mkm, 4,6*250mm, N 86912
Elution: A - H3PO4 0.01M pH 2.6; B - MeCN, fl. 1,0 ml/min, loop 20mkl.

Figure S103. Copy HPLC analysis of the derivative **16c**.

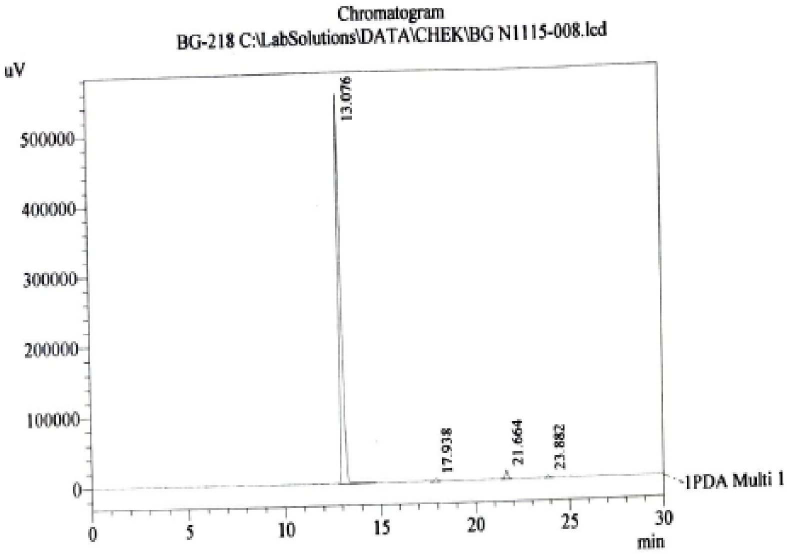

1 PDA Multi 1 / 290nm 4nm

PDA Ch1 290nm 4nm

Peak#	Ret. Time	Area	Height	Area %
1	13.076	5514284	552527	96.385
2	17.938	39610	3897	0.692
3	21.664	130350	12504	2.278
4	23.882	36860	3574	0.644
Total		5721104	572501	100.000

Method Filename : FOS A.lcm 16.11.2018 14:07:52

Time	Unit	Command	Valu
0.01	Pumps	B.Conc	5
30.00	Pumps	B.Conc	20
33.00	Pumps	B.Conc	5
45.00	Controller	Stop	

Shimadzu LC-20 AD; System - FOS Colon- Kromasil-100-5mkm. C-18, 4,6x250 mm. N 62511
Elution: A - H3PO4 0,01M pH 2,6; B - MeCN, fl - 1.0 ml/min, loop 20 mkl

Figure S104. Copy HPLC analysis of the derivative **16d**.

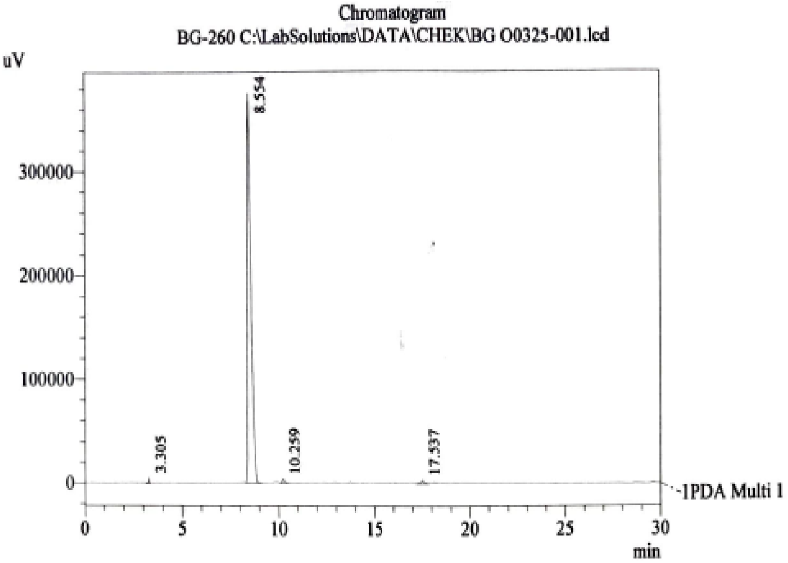

1 PDA Multi 1 / 335nm 4nm

PDA Ch1 335nm 4nm

Peak#	Ret. Time	Area	Height	Area %
1	3.305	21511	5742	0.437
2	8.554	4830693	374893	98.063
3	10.259	33626	4069	0.683
4	17.537	40262	4019	0.817
Total		4926092	388723	100.000

Method Filename : FOS B.lcm 25.03.2019 11:43:21

Time	Unit	Command	Valı
0.01	Pumps	B.Conc	10
30.00	Pumps	B.Conc	90
33.00	Pumps	B.Conc	10
45.00	Controller	Stop	

Shimadzu LC-20 AD; System - FOS Colon- Kromasil-100-5mkm. C-18, 4,6x250 mm. N 62511
Elution: A - H3PO4 0,01M pH 2,6; B - MeCN, fl - 1.0 ml/min, loop 20 mkl

Figure S105. Copy HPLC analysis of the derivative **17a**.

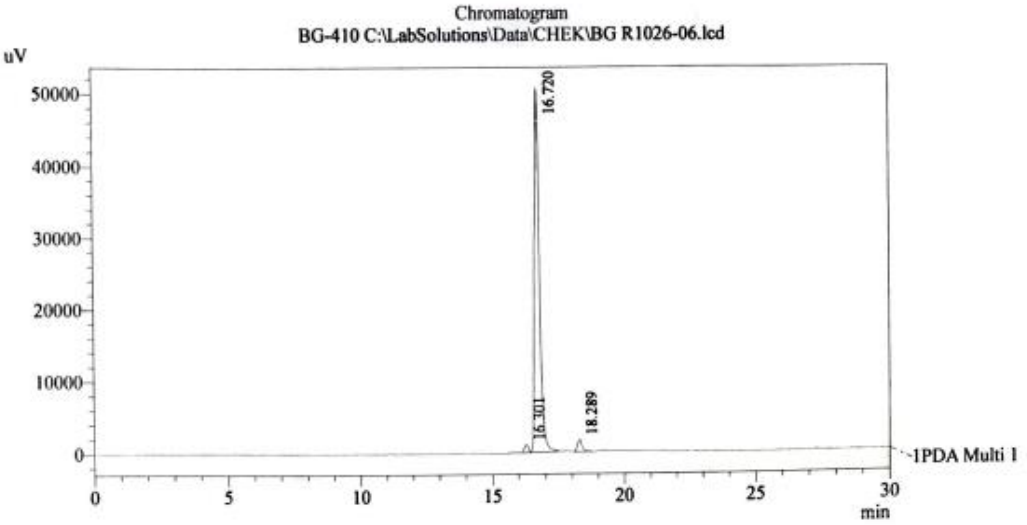

1 PDA Multi 1 / 470nm 4nm

PDA Ch1 470nm 4nm

Peak#	Ret. Time	Area	Height	Area %
1	16.301	12851	1135	1.933
2	16.720	631218	50599	94.967
3	18.289	20603	1692	3.100
Total		664672	53427	100.000

Method Filename : FRA02BvApr.lcm 26.10.2021 15:52:58

Time	Unit	Command	Valu
0.01	Pumps	B.Conc	20
30.00	Pumps	B.Conc	60
40.00	Pumps	B.Conc	70
43.00	Pumps	B.Conc	20
55.00	Controller	Stop	

Shimadzu LC-20 AD; System - FRA 02, Colon- Kromasil-100-5mkm. C-18, 4,6x250 mm. N86915
Elution: A - COOHNH4 0.2% pH 6,4; B - MeCN, fl - 1.0 ml/min, loop 20 mkl

Figure S106. Copy HPLC analysis of the derivative **17b**.

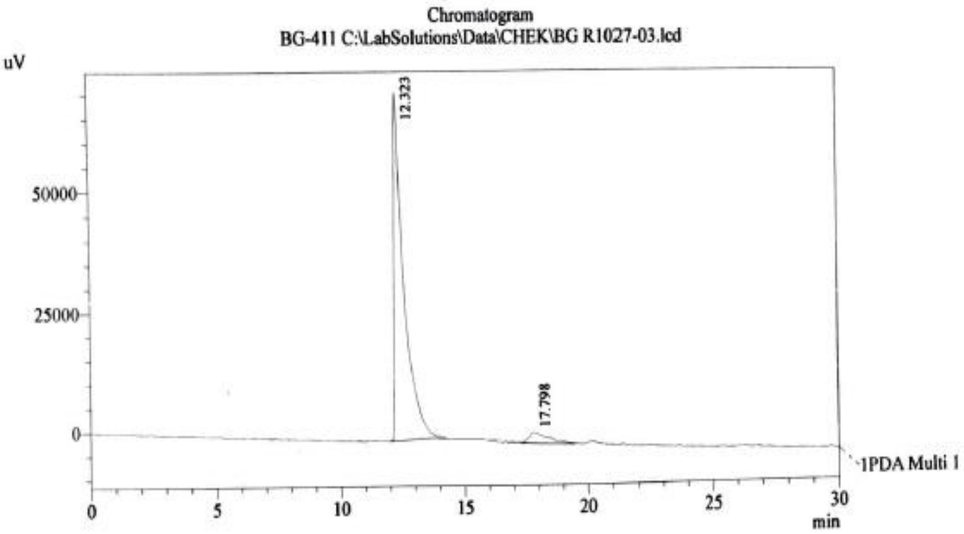

1 PDA Multi 1 / 330nm 4nm

PDA Ch1 330nm 4nm

Peak#	Ret. Time	Area	Height	Area %
1	12.323	2023918	72419	94.401
2	17.798	120044	2294	5.599
Total		2143962	74713	100.000

Method Filename : FRA02BvApr.lcm 27.10.2021 12:16:40

Time	Unit	Command	Valu
0.01	Pumps	B.Conc	20
30.00	Pumps	B.Conc	60
40.00	Pumps	B.Conc	70
43.00	Pumps	B.Conc	20
55.00	Controller	Stop	

Shimadzu LC-20 AD; System - FRA 02, Colon- Kromasil-100-5mkm. C-18, 4,6x250 mm. N86915
Elution: A - COOHNH4 0.2% pH 6,4; B - MeCN, fl - 1.0 ml/min, loop 20 mkl

Figure S107. Copy HPLC analysis of the derivative **17c**.

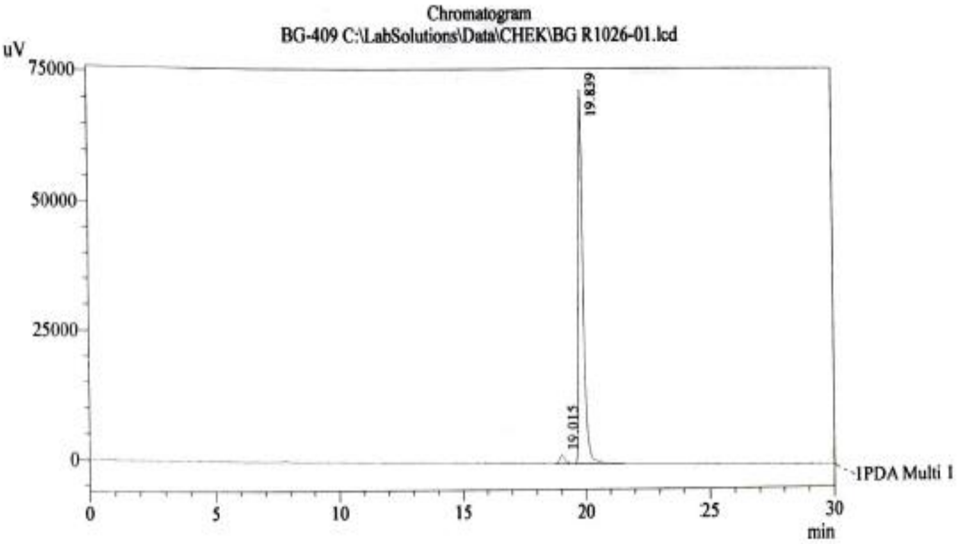

1 PDA Multi 1 / 395nm 4nm

PDA Ch1 395nm 4nm

Peak#	Ret. Time	Area	Height	Area %
1	19.015	23233	1676	2.308
2	19.839	983483	72850	97.692
Total		1006716	74526	100.000

Method Filename : FRA02BvApr.lcm 26.10.2021 11:29:18

Time	Unit	Command	Valu
0.01	Pumps	B.Conc	20
30.00	Pumps	B.Conc	60
33.00	Pumps	B.Conc	20
45.00	Controller	Stop	

Shimadzu LC-20 AD; System - FRA 02, Colon- Kromasil-100-5mkm. C-18, 4,6x250 mm. N86915
Elution: A - COOHNH4 0.2% pH 6,4; B - MeCN, fl - 1.0 ml/min, loop 20 mkl

Figure S108. Copy HPLC analysis of the derivative **17d**.

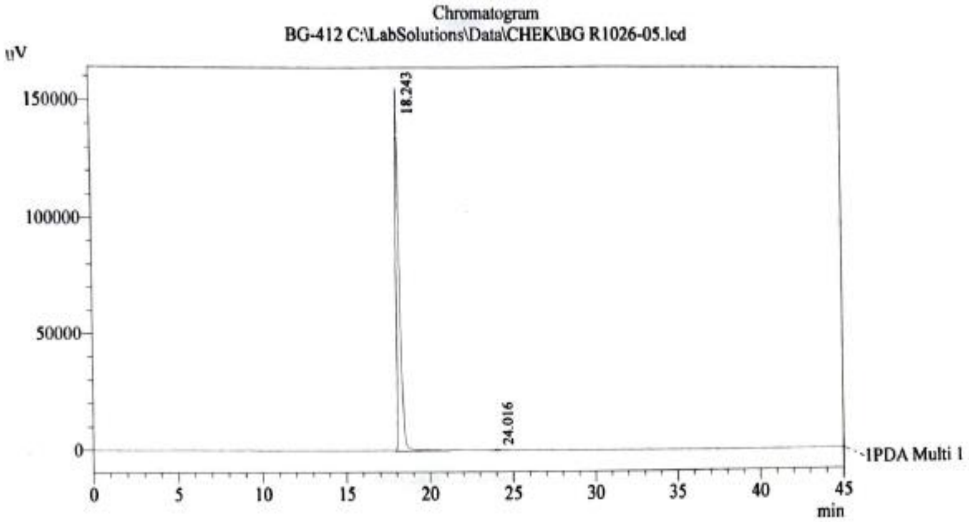

1 PDA Multi 1 / 390nm 4nm

PDA Ch1 390nm 4nm

Peak#	Ret. Time	Area	Height	Area %
1	18.243	2059219	155775	99.653
2	24.016	7162	543	0.347
Total		2066382	156318	100.000

Method Filename : FRA02BvApr.lcm 26.10.2021 14:55:35

Time	Unit	Command	Valu
0.01	Pumps	B.Conc	20
30.00	Pumps	B.Conc	60
40.00	Pumps	B.Conc	70
43.00	Pumps	B.Conc	20
55.00	Controller	Stop	

Shimadzu LC-20 AD; System - FRA 02, Colon- Kromasil-100-5mkm. C-18, 4,6x250 mm. N86915
Elution: A - COOHNH4 0.2% pH 6,4; B - MeCN, fl - 1.0 ml/min, loop 20 mkl

Figure S109. Copy HPLC analysis of the derivative **18a**.

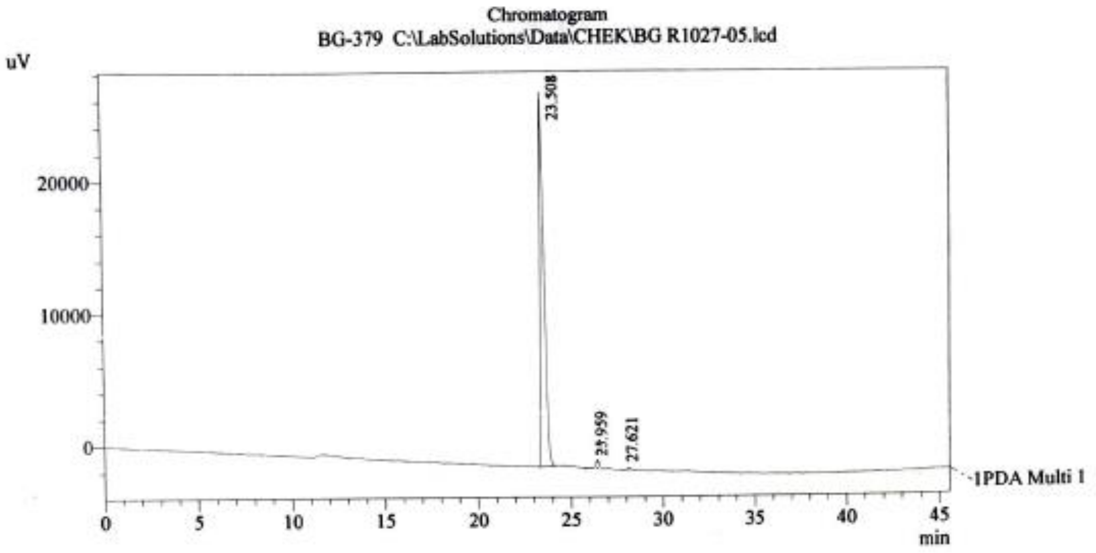

1 PDA Multi 1 / 470nm 4nm

PDA Ch1 470nm 4nm

Peak#	Ret. Time	Area	Height	Area %
1	23.508	432361	28232	97.780
2	25.959	6803	545	1.538
3	27.621	3016	230	0.682
Total		442179	29007	100.000

Method Filename : FOS Av1.lcm 27.10.2021 14:28:14

Time	Unit	Command	Valu
0.0!	Pumps	B.Conc	20
30.00	Pumps	B.Conc	60
40.00	Pumps	B.Conc	70
43.00	Pumps	B.Conc	20
55.00	Controller	Stop	

Shimadzu LC-20 AD; System - FRA 02, Colon- Kromasil-100-5mkm. C-18, 4,6x250 mm. N86915
Elution: A - COOHNH4 0.2% pH 6,4; B - MeCN, fl - 1.0 ml/min, loop 20 mkl

Figure S110. Copy HPLC analysis of the derivative **18b**.

Chromatogram
BG-414 C:\LabSolutions\Data\CHEK\BG R1027-02.lcd

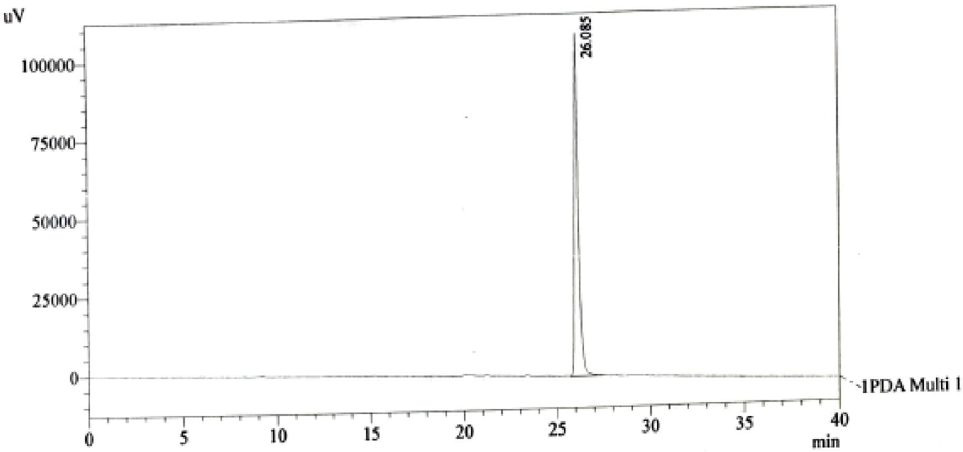

1 PDA Multi 1 / 393nm 4nm

PDA Ch1 393nm 4nm

Peak#	Ret. Time	Area	Height	Area %
1	26.085	1769536	108823	100.000
Total		1769536	108823	100.000

Method Filename : FRA02BvApr.lcm 27.10.2021 11:23:45

Time	Unit	Command	Valu
0.01	Pumps	B.Conc	20
30.00	Pumps	B.Conc	60
40.00	Pumps	B.Conc	70
43.00	Pumps	B.Conc	20
55.00	Controller	Stop	

Shimadzu LC-20 AD; System - FRA 02, Colon- Kromasil-100-5mkm. C-18, 4,6x250 mm. N86915
Elution: A - COOHNH4 0.2% pH 6,4; B - MeCN, fl - 1.0 ml/min, loop 20 mkl

Figure S111. Copy HPLC analysis of the derivative **18c**.

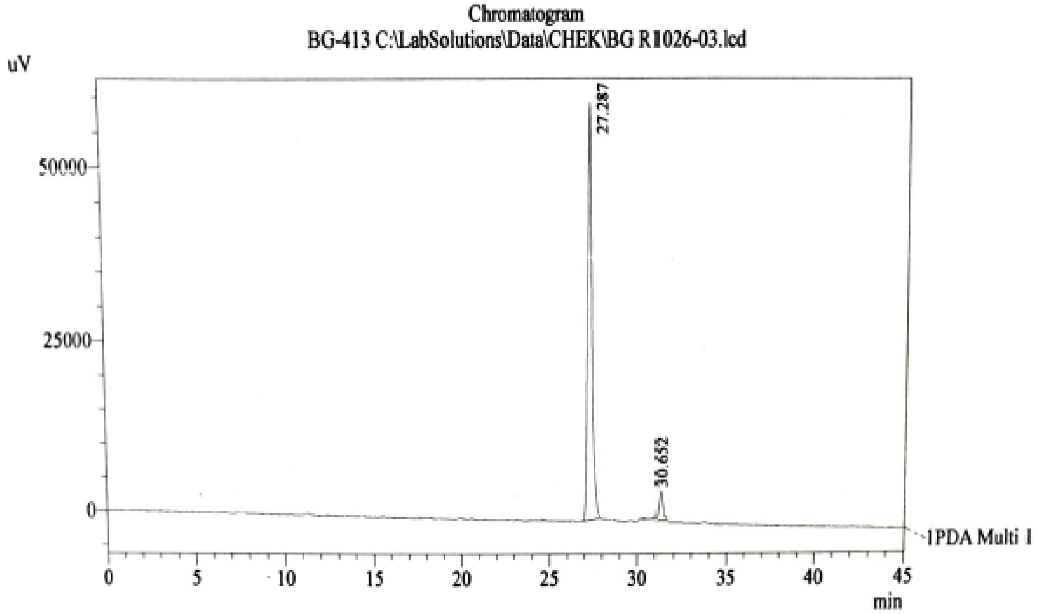

1 PDA Multi 1 / 395nm 4nm

PDA Ch1 395nm 4nm

Peak#	Ret. Time	Area	Height	Area %
1	27.287	1031612	60286	94.625
2	30.652	58602	3924	5.375
Total		1090214	64210	100.000

Method Filename : FRA02BvApr.lcm 26.10.2021 13:01:35

Time	Unit	Command	Valu
0.01	Pumps	B.Conc	20
30.00	Pumps	B.Conc	60
40.00	Pumps	B.Conc	70
43.00	Pumps	B.Conc	20
55.00	Controller	Stop	

Shimadzu LC-20 AD; System - FRA 02, Colon- Kromasil-100-5mkm. C-18, 4,6x250 mm. N86915
Elution: A - COOHNH4 0.2% pH 6,4; B - MeCN, fl - 1.0 ml/min, loop 20 mkl

Figure S112. Copy HPLC analysis of the derivative **18d**.

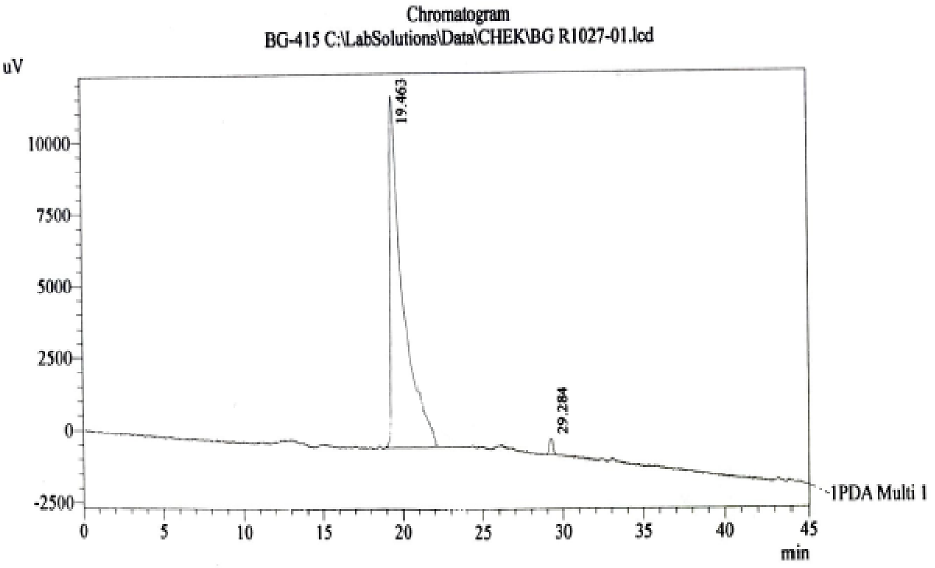

1 PDA Multi 1 / 440nm 4nm

PDA Ch1 440nm 4nm

Peak#	Ret. Time	Area	Height	Area %
1	19.463	740615	12108	98.758
2	29.284	9311	565	1.242
Total		749925	12673	100.000

Method Filename : FRA02BvApr.lcm 27.10.2021 10:27:49

Time	Unit	Command	Valu
0.01	Pumps	B.Conc	20
30.00	Pumps	B.Conc	60
40.00	Pumps	B.Conc	70
43.00	Pumps	B.Conc	20
55.00	Controller	Stop	

Shimadzu LC-20 AD; System - FRA 02, Colon- Kromasil-100-5mkm. C-18, 4,6x250 mm. N86915
Elution: A - COOHNH4 0.2% pH 6,4; B - MeCN, fl - 1.0 ml/min, loop 20 mkl

The whole-genomic sequencing of *M. smegmatis* mutant MSMEG_4883

>MSMEG_4883
ATGAGCATATCGCTGCTGCTCGAGATGGCATCGTCGGGAGATCCCGACCGCACCGCGGTGGTTTCCGACG
ACACCCGGCTCACCGCAGGCGAGTTGAGCACATTGGCCGACGGGGCAGCAGGCGTCATCGCCGGGTCAGG
CGCGGCACACGTCGCCTACGTGGGTACCGGTGGCG**CGCTGCTGCCGCTGCTGC**TGTTCGCCTCGGCACGC
GCGGCGATCCCCTTCACCCCCCTGAACTACCGTCTCAGCGCCGAAGGTCTGCGCGAGCTGGTCGACCGGT
TGCCCACACCGCTGGTGATCGCCGACGGCGAGTACGCCGGCATGCTCGCCGGAGCGGGCAGGCCGGTGCT
CACCTCCGAGGAGTTCCTGTCCCAGGCCCGCACGGCCGATCCGGCCGCAGAGTTCGCCGACCCCGACGCG
GTCGCGGTGGTGCTGTTCACCTCGGGCACCACATCGCGCCCCAAGGCCGTCGAACTCACCCACAACAACC
TCACGAGCTACATCACCGGGACCGTCGAATTCGGTTCGGCCGCACCGGAGGACGCCGCGCTGATCTGTGT
GCCGCCGTACCACATCGCGGGTGTCAGCGCGGCGATGTCCAACCTGTACGCCGGCCGGAAGATGGTGTAC
CTGCGCAACTTCGACGCACACCGCTGGGTCGAGTTGGTGCGCACCGAGGGCGTCACGTCTGCCACCGTGG
TGCCGACCATGCTCGACCGTATCGTCACCGCACTTGAGACCACGCGCGCCGAGCTGCCGACCCTGCGCAA
CCTCGCCTACGGCGGATCCAAGGTCGCGCTGCCCCTGGTCCGCAAGGCGCTGGAGCTGATGCCGAACGTC
GGATTCGTCAACGCCTACGGTCTCACCGAAACCAGCTCCACCATCGCGGTCCTCGGACCCGACGACCACC
GGGCCGCGCTGGCCTCCGACGACCCCGGTGTGACCCGCCGCCTCGGGTCGGTGGGACAGGTGGTGCCCGG
GATCGAGGTGCAGATCCGCGGCGAGGACGGCACCGTGCTGGGGCCCGGCGAGACCGGTGAGCTGTTCGTG
CGCGGCGAGCAGGTGTCCGGCCGGTACACCGAGATCGGGTCGGTGCTCGACGAGGACGGCTGGTTCCCCA
CCAAAGACGTTGCGATGCTTGACCAGGACGGCTATCTGTTCATCGGCGGCCGGTCGGACGACACCATCAT
CCGCGGCGGCGAGAACATCGCTCCCGCCGAGATCGAGGACGTTCTCGTGGAACATCCCGACGTGCGCGAC
GTCGCGGTGGTCGGCCCCGAGGACCCCCAGTGGGGCCAGATCATCGTCGCGGTCGTGGTGCCCGCCGACG
GCGCCGAACCCGACGCCGACGTACTCCGCGAGCACGTCCGCAAACACCTGCGCGGATCCCGCACCCCCGA
CCGCGTGGTCTTCCGCGCCGAACTGCCCACCAACGCCACCGGCAAGGTGCTGCGCCGTCAACTCGTCGAC
GAACTCCAGCCCATCTCGTAG

Figure S113. Nucleotide sequence of MSMEG_4883. The 9 bp repeats are underlined, while the deletion region is highlighted as bold.

Table S2. Unique SNPs in quinoxaline-resistant *M. smegmatis* mutants.

		M. smegmatis tfqR1			
Locus tag	Protein ID	annotation	Codon	SNP	amino acid substitution
MSMEG_0232	YP_884647.1	sugar transporter family protein	codon=170	TGG>GGG	W>G
MSMEG_1914	YP_886280.1	RNA polymerase sigma-70 factor, family protein	codon=30	TTC>TGC	F>C
MSMEG_3954	YP_888243.1	trehalose 6-phosphate phosphorylase		4024145 C->CG (frameshift)	
MSMEG_4043	YP_888329.1	amidohydrolase	codon=208	TTC>CTC	F>L
MSMEG_4495	YP_888767.1	hypothetical protein	codon=312	CTG>CCG	L>P
MSMEG_5893	YP_890119.1	hypothetical protein	codon=100	GTG>GCG	V>A
		M. smegmatis tfqR2			
Locus tag	Protein ID	annotation	Codon	SNP	amino acid substitution
MSMEG_0341	YP_884754.1	F420-dependent LLM class oxidoreductase*	codon=308	GTC>GCC	V>A
MSMEG_0341	YP_884754.1	F420-dependent LLM class oxidoreductase*	codon=229	CGG>CTG	R>L
MSMEG_0342	YP_884755.1	hypothetical protein	codon=221	ATC>AAC	I>N
MSMEG_0363	YP_884776.1	TetR family transcriptional regulator	codon=181	GAG>GAC	E>D
MSMEG_0933	YP_885336.1	hypothetical protein		1013795 A->AC (frameshift)	
MSMEG_1497	YP_885879.1	acyl-CoA dehydrogenase	codon=330	GAC>AAC	D>N
MSMEG_4272	YP_888549.1	HesB/YadR/YfhF family protein	codon=94	ACC>CCC	T>P
MSMEG_4323	YP_888598.1	pyruvate dehydrogenase subunit E1	codon=100	CCC>TCC	P>S
MSMEG_4778	YP_889035.1	thiolase	codon=198	ATG>ATC	M>I
MSMEG_5159	YP_889405.1	DNA-binding response regulator	codon=40	GCG>ACG	A>T
		M. smegmatis tfqR4			
Locus tag	Protein ID	annotation	Codon	SNP	amino acid substitution
MSMEG_2172	YP_886526.1	dicarboxylate-carrier protein	codon=181	TTC>GTC	F>V
MSMEG_3864	YP_888155.1	cobaltochelatase subunit CobN	codon=1109	TGG>TTG	W>L
MSMEG_3886	YP_888177.1	twin arginine-targeting protein translocase TatC	codon=206	CTG>CAG	L>Q
MSMEG_4702	YP_888963.1	ABC transporter permease	codon=80	GAA>GAC	E>D
MSMEG_4863	YP_889119.1	LLM class flavin-dependent oxidoreductase*	codon=224	GCG>GGG	A>G
MSMEG_5943	YP_890168.1	peroxisomal multifunctional enzyme type 2	codon=106	TCG>TAG	S>*
MSMEG_6294	YP_890512.1	caib/baif family protein	codon=114	CCG>CTG	P>L
MSMEG_6801	YP_891008.1	kinase, pfkB family protein	codon=153	CAC>CAG	H>Q
		M. smegmatis tfqR5			
Locus tag	Protein ID	annotation	Codon	SNP	amino acid substitution
MSMEG_4189	YP_888468.1	cysteinyl-tRNA synthetase	codon=52	CAT>AAT	H>N
MSMEG_6392	YP_890605.1	polyketide synthase	codon=229	GAT>GGT	D>G
		M. smegmatis tfqR6			
Locus tag	Protein ID	annotation	Codon	SNP	amino acid substitution
MSMEG_0889	YP_885292.1	succinate-semialdehyde dehydrogenase	codon=306	AAG>GAG	K>E
MSMEG_1515	YP_885897.1	two-component sensor histidine kinase	codon=291	GGG>AGG	G>R
MSMEG_1518	YP_885900.1	hypothetical protein	codon=144	TCC>TAC	S>Y
		M. smegmatis tfqR7			
Locus tag	Protein ID	annotation	Codon	SNP	amino acid substitution
MSMEG_0529	YP_884940.1	serine/threonine protein kinase	codon=1013	ATG>GTG	M>V
MSMEG_1380	YP_885766.1	transcriptional regulator	codon=15	GCG>TCG	A>S
MSMEG_6440	YP_890653.1	monooxygenase, flavin-binding family protein	codon=132	TCG>TGG	S>W
		All mutants			
Locus tag	Protein ID	annotation	Codon	SNP	amino acid substitution
MSMEG_4883	YP_889139.1	AMP-dependent synthetase/ligase		4979295 GCGCTGCTGC->G	

* Functionally annotated by BLAST search.
